# Bimodal gene expression patterns in breast cancer

**DOI:** 10.1186/1471-2164-11-S1-S8

**Published:** 2010-02-10

**Authors:** Marina Bessarabova, Eugene Kirillov, Weiwei Shi, Andrej Bugrim, Yuri Nikolsky, Tatiana Nikolskaya

**Affiliations:** 1 Russian Academy of Sciences, Vavilov Institute for General Genetics, ul. Gubkina, 3, Moscow, Russia; 2 Genego, Inc., 500 Renaissance Drive, St. Joseph, MI 49085, USA

## Abstract

We identified a set of genes with an unexpected bimodal distribution among breast cancer patients in multiple studies. The property of bimodality seems to be common, as these genes were found on multiple microarray platforms and in studies with different end-points and patient cohorts. Bimodal genes tend to cluster into small groups of four to six genes with synchronised expression within the group (but not between the groups), which makes them good candidates for robust conditional descriptors. The groups tend to form concise network modules underlying their function in cancerogenesis of breast neoplasms.

## Background

Whole-genome gene expression studies primarily aim to identify conditional descriptors, i.e. subsets of genes or functional groups whose expression profiles distinguish between different biological states. Different biological conditions might include: disease state vs. normal state, good prognosis vs. bad, drug treated vs. untreated tissues, etc. Differential expression descriptors can be calculated in two ways. The traditional method consists of selecting a set of descriptor genes (gene signatures) using a variety of statistical methods [1-5]. Using this approach, a number of gene signatures were deduced for breast cancer phenotypes, including an "intrinsic" set for clustering of breast cancers [6], an "Amsterdam" signature consisting of 70 genes [7], a 76-gene "Rotterdam" signature [8] for metastasis, and a set of 21 genes associated with disease outcomes for ER+ tumors [9]. Some of these sets are commercialized as multivariant diagnostics by Genomic Health http://www.genomichealth.com and Agendia http://www.agendia.com. Although important, gene signatures have many issues as descriptors - for instance, loss of specificity in validation studies with an increased number of samples [10], generally poor cross-platform compatibility (Amsterdam and Rotterdam signatures virtually do not overlap in gene content), lack of mechanistic (functional) correlation with phenotype, etc.

The second, more recent, approach deals with so-called "functional descriptors," such as pathways, signaling networks, enrichment distribution in ontologies, etc., which are differentially perturbed in the conditions being compared [11-14]. In good accordance with the original concept of "modularity" of biological functions systems [15], functional entities seem to be more robust descriptors than gene lists [16,17]. In addition, functional descriptors provide strong mechanistic linkages with clinical phenotypes and, in the case of cancer, may explain important aspects of cancerogenesis.

However, in both cases, the genes composing gene signatures or functional categories are selected regardless of their individual patterns of expression among the samples in the study. In general, gene expression distribution in a population is assumed to be normal, as for any quantitative trait [18]. However, it is not. As we have recently shown, distributions of expression signals of certain genes feature two distinct peaks among the samples in breast cancer [19]. The phenomenon of expression "bimodality" was reported for other cancers as well [20-22], where «bimodality» was calculated by selection of hypervariable (HV) genes using F-statistics [20] and a combination of mixture modelling and kurtosis [21,22].

Here we report a meta-analysis of bimodally expressed genes from five previously published independent breast cancer studies. We show that "bimodality" is a general phenomenon (at least for breast cancer), independent of a microarray platform and clinical phenotype (patient cohort). Bimodality is intrinsically associated with physiological states of the system, such as cancer vs. normal. Moreover, bimodally-expressed genes tend to cluster into groups with synchronised expression within a group. Consequently, bimodal group expression can be effectively used as an efficient and robust conditional descriptor, applicable for a variety of studies.

We also demonstrate the platform-independence of bimodality in three different microarrays used in the studies. Although compatibility between arrays can be high for certain end-points in limited size studies, as shown in the MAQCII project [23], in general, gene signatures are not robust and cannot be directly compared across platforms. There are several statistical methods of meta-analysis which enable direct comparison between gene expression levels in multiple experiments and allow for identification of genes with consistent signal values across the studies [20-27]. Here, we offer an approach to normalization of expression signal values into a binary mode corresponding to different conditions, which makes expression profiles on different arrays directly compatible.

## Results

### The phenomenon of bimodality of gene expression

Originally, we identified a set of bimodally expressed genes within the previously published dataset of 295 early breast cancer samples run on two custom cDNA array platforms [19,28]. In the validation study, we confirmed the phenomenon of bimodality and the ability of bimodal genes to form co-expressed clusters using four datasets carried out on standard Affymetrix and Agilent array platforms: GSE1456 [29], GSE7390 [30], GSE4922 [31], and an Agilent data set (Table 1). The Agilent dataset was formed as a non-redundant set of 193 samples from four studies: GSE1992 [32], GSE2740 [33], GSE2741 [34], and GSE6130 [35]. The robustness of the original bimodal clusters was tested both across-platform and across-study (same array type) (see additional file 1).

**Table 1:** Gene expression datasets used for identification of genes with bimodal expression patterns. In all five datasets, bimodality was defined by *τ *= 2.64 and standard deviation over 25th percentile of the distribution. ^a ^Recognized genes for each platform.

	Sorlie295	GSE1456	GSE7390	GSE4922	Agilent set
**Platform**	cDNA	Affymetrix	Affymetrix	Affymetrix	Agilent
**Bimodal genes**	2476 (10604^a^)	5075 (12017^a^)	5440 (12017^a^)	4874 (12017^a^)	4983 (13379^a^)

**Figure 1:**
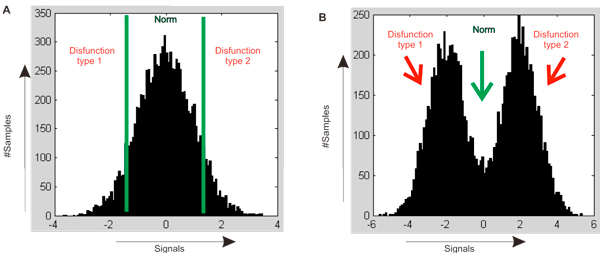
**Signal distribution of normal and "bimodal" genes in patient cohort**. (A) Theoretical normal gene signal distribution for quantitative traits [18]. (B) Theoretical bimodal gene signal distribution

First, we compared the distribution of expression values throughout the set of 295 primary tumor samples of invasive breast cancers [28] for each gene and noticed that certain genes tended to have two different levels of expression, or modes, among the samples. In other words, the expression function seemed to feature two distinct peaks, rather than to be a continuous function with close to normal distribution, as is expected for any quantitative trait [18] (Figure 1A).

**Figure 2:**
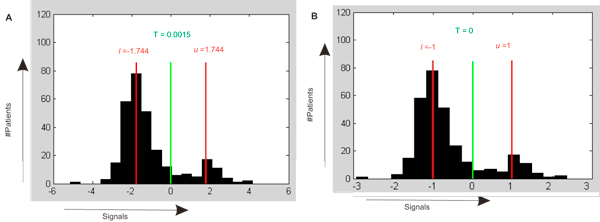
**Bimodal genes**. (A) Distribution of GRB7 expression among 295 patients (Sorlie295 dataset). The green line marks the threshold which separates the average of signals below threshold TGRB7≈0.0015. Red lines mark lGRB7≈1.74 and uGRB7≈1.77. (B) Distribution of GRB7 expression among 295 patients after normalization. The green line marks the threshold which separates the average of signals below threshold TGRB7 = 0. Red lines mark lGRB7≈-1 and uGRB7 = 1.

In order to calculate a "bimodality" function for each gene in the 295 patients' set, we introduced a t-test like statistic *τ*, which is a partition function that describes the relative difference between average of signals between each peak. In brief, the larger the t, the larger the difference between the two peaks (i.e. modes) in the distribution of a certain gene signal profile within the cohort. (Calculations and assumptions are described in "Methods.") For a normal distribution of normalized expression signals for a certain gene, *τ *≈ 2.64. We assume that the wider (potentially bi-nomal or "multi-nomal") distribution is characterized by *τ *> 2.64. At this step, we applied *τ *statistics to "filter" the profiles of all genes to identify the most likely candidates for bimodal distribution and selected the genes with the furthest possible difference between the peaks. Thus, a typical bimodal gene GRB7 has *τ *= 4.81 and a distribution between samples shown in Figure 2A. In total, we identified 2476 bimodal genes out of the array of 10604 genes [28]. Using these parameters, we calculated sets of bimodal genes using the validation datasets of 5075, 5440, 4872, and 4983 genes from the independent datasets GSE1456, GSE7390, GSE4922, and the Agilent data set respectively (Table 1).

Binary intersections of the pairs of bimodal genes from different datasets are large and statistically significant (Table 2). The largest intersection was for the datasets GSE7390 and GSE1456 at 3587 common bimodal genes - 66% of all bimodal genes for GSE7390 and 70% of all bimodal genes for GSE1456. The datasets Sorlie295 and GSE4922 had the smallest intersection of 1121 common bimodal genes - 45% of all bimodal genes for Sorlie295 and 23% of all bimodal genes for GSE4922. In total, we considered 866 genes as «commonly bimodal» in all platforms and studies (see additional file 1). We considered a gene as "commonly" bimodal if its expression pattern was bimodal at at least three independent datasets

**Table 2:** Pair-wise intersections of the sets of bimodal genes in five studies. Fisher exact tests were used to estimate p-values.

SetA	SetB	All genes intersection	Bimodal genes intersection	**Bimodal genes for set A**^a^	**Bimodal genes for set B**^a^	p-value
**Agilent**	**Sorlie295**	9433	1237	3661	2219	**8.81E-77**
**Agilent**	**GSE1456**	10301	1830	3961	4307	**5.86E-13**
**Agilent**	**GSE4922**	10301	1799	3961	4099	**2.14E-20**
**Agilent**	**GSE7390**	10301	1839	3961	4551	**0.000154**
**Sorlie295**	**GSE1456**	9367	1173	2223	3851	**3.49E-37**
**Sorlie295**	**GSE4922**	9367	1121	2223	3720	**5.53E-32**
**Sorlie295**	**GSE7390**	9367	1237	2223	4048	**1.13E-41**
**GSE1456**	**GSE4922**	12017	3501	5076	4876	**0**
**GSE1456**	**GSE7390**	12017	3587	5076	5440	**0**
**GSE4922**	**GSE7390**	12017	3431	4876	5440	**0**

Therefore, we conclude that bimodality of gene expression is a phenomenon not limited to a specific microarray platform, a study/endpoint or a dataset/patient cohort. Bimodality of individual genes is confirmed for at least three different studies, and in some cases in four or five studies.

### "Bimodality" is conditional (disease-related)

We believe that "bimodality" is a conditional expression property of a gene and each «mode» corresponds to a certain physiological condition, for example, a normal and a disease state. It is also possible that the two modes could correspond to different disease subtypes.

**Figure 3:**
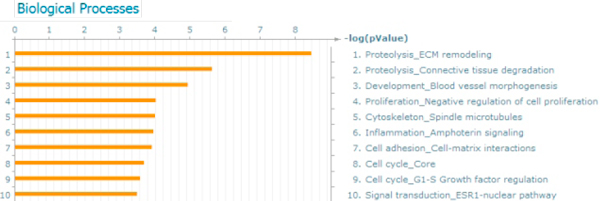
**Ontology enrichment for the set of 866 bimodal genes**.

The bimodal genes are relevant for disease development; in the case of breast cancer, functional analysis of bimodal genes in the data mining platform MetaCore (GeneGo, Inc.) reveals a role in cancerogenesis processes and pathways. First, 207 of 866 common bimodal genes have been described in literature as associated with breast cancer (Fisher test p-value = 1.499e-112 for the intersection) (see additional file 1). In total, there are 1393 breast cancer associated genes in MetaCore, within a total background of 40599 human genes (Entrez Gene statistics, http://www.ncbi.nlm.nih.gov/gene). These genes belong to many cancerogenesis processes and pathway maps including "Proteolysis: ECM remodeling," "Proteolysis: Connective tissue degradation," "Development: Blood vessel morphogenesis," "Proliferation: Negative regulation of cell proliferation," "Cytoskeleton: Spindle microtubules," "Inflammation: Amphoterin signaling," "Cell adhesion: Cell-matrix interactions," "Cell cycle: Core," "Cell cycle: G1-S Growth factor regulation," "Signal transduction: ESR1-nuclear pathway" (Figure 3). Four processes - "Proteolysis: ECM remodeling," "Development: Blood vessel morphogenesis," "Proteolysis: Connective tissue degradation," and "Cell adhesion: Cell-matrix interactions" - are prevalent in the later stages of invasive cancerogenesis when the tumor is large in size. By late stages, the tumor has a limited supply of oxygen and nutrients accompanied by acidosis by CO_2 _and accumulation of un-processed metabolites. These events trigger angiogenesis, lymphogenesis, cell matrix remodeling, and chemotaxis, often followed by metastasis. The process "Proliferation: Negative regulation of cell proliferation" is directly linked with these events, as the organism tries to regulate cell proliferation in the tumor. The process "Cell adhesion: Platelet-endothelium-leucocyte interactions" is associated with the tumor's capacity to metastasize. The activated processes "Cell cycle: Core," "Cytoskeleton: Spindle microtubules," and "Cell cycle: G1-S Growth factor regulation" reflect different aspects of the normal cell cycle in which perturbations can lead to cancer. The process "Reproduction: Progesterone signaling" is a breast cancer-specific process. Moreover, the set of bimodal genes is enriched with drug targets - 69 targets among 866 genes (Fisher test p-value = 1.169e-29 for the intersection, as there are 609 human protein drug targets (MetaBase statistics, http://www.genego.com), background list - 40599 human genes (Entrez Gene statistics, http://www.ncbi.nlm.nih.gov/gene) (see additional file 1). Therefore, we summarize that the set of 866 bimodal genes is cancer-specific and comprised of good putative markers for breast cancer.

### Normalization of expression for bimodal genes

In order to clearly separate the patient samples by bimodal gene expression, we normalized the signals, so the signals could be presented in a binary manner, with one peak designated as -1 and another as 1. The original expression signals varied significantly between the genes in the same sample, and individual bimodal genes could be both over- and under-expressed in different samples. Therefore, the step of normalization was neccessary for minimizing the difference in amplitude of the expression of the genes in order to profile separate experiments in a uniform way. There can be two cases: 1. one gene from different experiments in which the intensities of its expression are different, and 2. different genes have similar intensity within one experiment. In the former case, normalization makes comparable the profiles of genes with different original intensities of expression. In the latter case, it allows one to identify truly similar genes within one set, with synchronised expression profiles for a physiological condition. The process of normalization is described in detail in "Methods," and an example of normalization of GRB7 expression from the Sorlie295 dataset is shown in Figure 2B.

**Figure 4:**
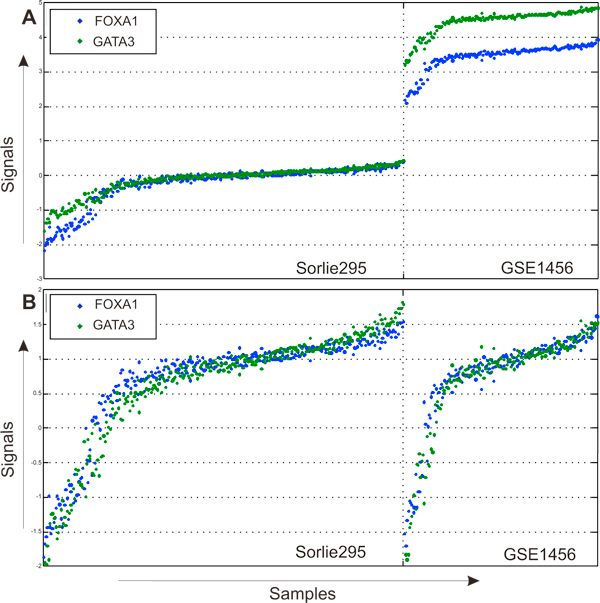
**Signal normalization for bimodal genes**. (A) Expression profiles for genes FOXA1 and GATA3 in Sorlie295 and GSE1456 data sets before normalization. (B) Expression profiles for genes FOXA1 and GATA3 in Sorlie295 and GSE1456 data sets before normalization and after normalization.

Importantly, some bimodal genes were observed to be expressed synchronously among samples in different studies when the normalized (not the original) signals were compared. An example for two genes - FOXA1 and GATA3 - is shown in Figure 4A. Prior to normalization, these genes had similar expression profiles, but had differences in intensity amplitude. After normalization, their gene expression profiles look identical (Figure 4B). Therefore, normalization helped to separate a subset of bimodal genes with synchronised expression in accordance with physiological conditions.

Signal normalization also helped to reduce the platform-dependency of expression signals. The normalized expression of the same two genes, FOXA1 and GATA3, was compared between experiments run on two array platforms: cDNA array, Sorlie 295 [28] and Affymetrix (Affymetrix Human Genome U133A Array) GSE1456 [29]. The original expression profiles of the two genes had different intensity intervals (Figure 4A), while the normalized expression values ranged between -1 and 1. (Figure 4B). We generated expression profiles for all bimodal genes (Table 3) in five datasets using original signal values (see additional file 2, additional file 3) and normalized values (see additional file 4, additional file 5). Unlike the original signals, the normalized values were not dependent on the array platform.

**Table 3:** The "close neighbors" groups of synchronously expressed bimodal genes for Sorlie295 data set.^a ^*Italics *- breast cancer-associated genes.

Group 1	Group 2	Group 3	Group 4	Group 5
**ERBB2**	**ESR1**	**PLAUR**	**FN1**	**STAT1**
*GRB7*^*a*^	*ESR1*	COL11A1	*FN1*	*STAT1*
*ERBB2*	*GATA3*	*PLAUR*	COL5A2	ISG15
*PSMD3*	*FOXA1*	*GABRP*	COL1A2	MX1
*TCAP*	*AR*	*TMEM158*		*CXCL10*
	DNALI1	TGBI		PLSCR1
		*ADM*		

### "Close neighbors" - groups of synchronously-expressed bimodal genes

**Figure 5:**
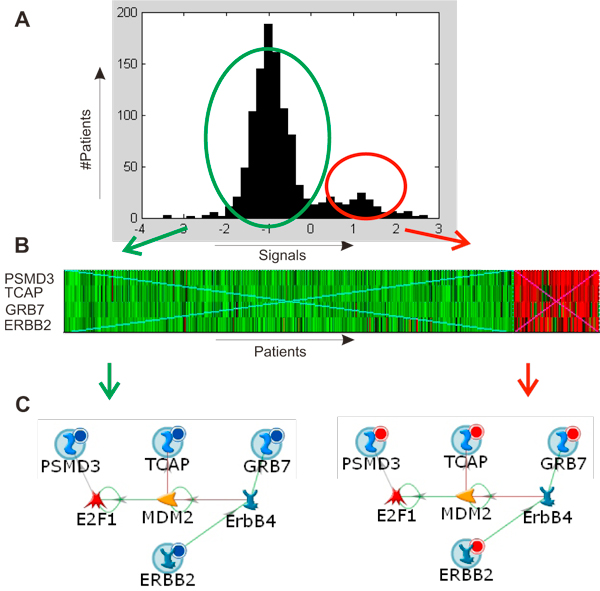
**Identification of "Close neighbours" co-expression groups**. (A) Average ERBB2 group expression profile. (B) Average ERBB2 group expression profile divides cohort of breast cancer patients into two groups. (C) "Close neighbours" expression group ERBB2 forms a network, functional module.

Following the theory of modularity of biological processes [15], we attempted to identify co-expressed modules (functional modules), assuming that the gene members of the module should be co-expressed among all samples in the cohort. We took as «baits» five bimodal genes reported as important breast cancer genetic markers - ERBB2, ESR1, PLAUR, FN1, and STAT1, and calculated the "close neighbor" gene groups that were synchronously expressed with each of them in the Sorlie295 set. Normalized expression profiles were considered as the measure of «closeness». In order to identify a group of synchronously expressed genes for a given gene, we calculated the cosine distance between the "query" gene with all other genes on a given array with proper expression values. The outliers to "0" were added to the list of candidate genes. This method allowed us to identify groups of genes with similar normalized expression profiles within the group that were also sufficiently different from other genes. In total, we identified 5 groups with 23 synchronously-expressed genes (Table 3). Importantly, all 23 genes happened to be bimodal, and 15 out of 23 were reported to be genetically associated with breast cancer (breast cancer "causal" genes) (Table 3). Expression profiles for the genes from the ERBB2 group are shown in Figure 5B. The fact that normalized expression of all 23 genes was synchronised within a group (but not between the groups) for all 5 groups with no exception, regardless of the set, clinical end-point and array platform is remarkable, as expression experiments are notoriously known as poorly comparable between studies and platforms, and breast cancers are extremely heterogenous. Thus, without normalization, we have not been able to identify a single gene commonly expressed in breast cancer samples among the studies using standard statistical procedures (t-test for DEGs, FDR, ANOVA).

**Figure 6:**
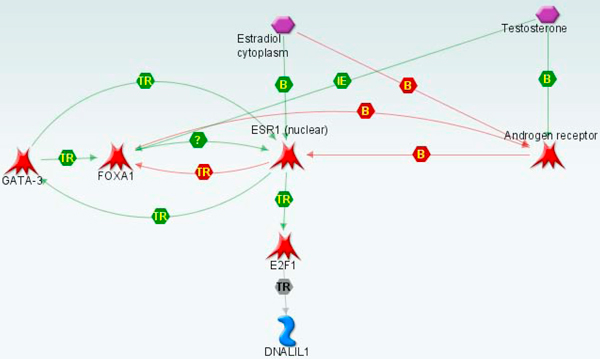
**Co-expression of bimodal genes in ESR1 group**. Genes from ESR1 group are regulated by an estradiol/testosterone regulation system

The genes within the groups were closely functionally connected. Every group forms a compact network with physical protein interactions connecting most group members in one or two steps. The network for the ERBB2 group is shown in Figure 5C. In addition, the genes TCAP, PSMD3, GRB7, and ERBB2 from the ERBB2 group are derived from the same well known breast cancer amplicon [36]. Transcription of MX1, CXCL10, PLSCR1 and ISG15 from the STAT1 group is directly regulated by STAT1 [37,38]. Similarly, the genes from ESR1 group are united by a common regulation system (Figure 6).

### "Close neighbors" expression groups as potential descriptors for breast cancer end-points

As every gene in the group is bimodal, and the expression profiles of genes in each group are synchronised, each group can be used as an effective descriptor dividing patients into two clusters corresponding to the two expression modes. An average expression value for all genes in the group was used as the measure of the group's expression. For instance, ERBB2 group expression is downregulated in some patients and up-regulated in another part of the cohort (Figure 5C). It was shown that the expression group profiles are more robust descriptors than individual genes [39].

The expression of the "ñlose neighbors" groups is a remarkably robust descriptor between microarray platforms. "Robustness" can be defined as retained performance on larger validation datasets and «across platforms», i.e. the descriptor genes have to be synchronously expressed on different types of arrays. It is particularly important in the cases when the descriptors are deduced using a training set on one array platform and validation sets on a different platform, and especially when descriptor genes are present on the training array but are missing on the validation array [40-44,23]. Using groups of genes (instead of individual genes in «gene signatures») and their summarized «group» expression instead of individual gene expression allows one to reduce or eliminate this problem. Thus, the gene TMIM158 from the PLAUR group is missing on Agilent arrays, but the group itself can still be used effectively as the descriptor with one gene missing. The average or summarized expression of the remaining genes in the group can be used as the group expression metric in this case.

Importantly, the pattern of group expression (i.e. an average of gene expression within a group) is remarkably stable between different studies and unique for the group, group expression profiles are essentially different and among the samples in all studies, i.e. the groups are expressed independently from each other. Therefore, the groups can be applied as robust descriptors for dividing samples (patients in the cohort) into sub-clusters (see additional file 6). The group descriptors can be applied consequently: Group 1 divided patients into two clusters, then Group 2 sub-categorizes each part into two and so on. Eventulally, every sample will be "barcoded" with 5 numbers reflecting the Group's expression mode as "1" or "2", for instance 1-1-2-1-2 (see additional file 6), and samples can be grouped together based on the matching "barcodes".

## Discussion

Here we described a fundamental property of certain genes to be expressed in two «modes» or expression levels depending on physiological condition/disease state. We studied this phenomenon in invasive breast cancer in five different studies using different array platforms, including cDNA arrays, Affymetrix and Agilent [28-35]. We have shown that bimodal genes are present on all arrays, and that the sets of bimodal genes statistically significantly overlap among the platforms. Therefore, we assume that bimodality is a common property of gene expression, dependent on physiological or disease states and independent of the end-points of the study or the microarray platform. In total, we identfied 866 bimodal genes shared among all platforms.

We developed and applied a computationally efficient algorithm to estimate bimodality of expression intensity distributions of genes based on maximization of the *t-statistic*-like measure *τ *(see Methods). Gene expression distribution is often modeled by a mixture of Gaussians with model parameters fit through expectation maximization (see e.g., [21,22]). Bimodality of expression can then be deduced from testing log-likelihood ratios of two component mixture distribution versus a single component normal distribution as in [21], or through calculation of Bayesian information criterion as in [22]. These approaches are computationally demanding and do not offer clear advantages over *t-statistics*. Also, characterization of a gene's bimodality via excess kurtosis as in [22] disregards bimodal distributions with unbalanced sizes of peaks, while a t-statistic still captures such unbalanced bimodality. A different approach for characterizing "hypervariable" genes was applied in [20], where authors searched for genes with higher variability than in a majority of genes. The F-test was used to select the genes with variances significantly higher than the variance of genes in a 'reference group'.

Conditional bimodality is an unexpected and non-trivial property of gene expression. The expected distribution of any quantitative character in biological systems is expected to be normal [18]. Moreover, most genes in the studied datasets are not «bimodal» (Table 1). Distribution with two distinct peaks means that transcriptional regulation of some genes is conditional - breast cancer-dependent in our case. Alternatively, one could expect expression of two different conditionally prevailing splice variants for certain transcripts - a phenomenon shown for some cancers [45]. However, observation of this case is not likely, as we see the same genes on three different platforms, and the array set does not allow us to separate different splice variants, at least for the original cDNA array.

Bimodal expression is conditional and, in our case, is linked to the complex condition known as breast cancer. The set of 866 common bimodal genes is heavily enriched in breast cancer-associated genes, participating in many pathways and processes of cancerogenesis. Thus, the «group query» genes ERBB2, ESR1, CEACAM5 and AR are well known markers of breast cancer [46-49].

According to the theory of modularity of biological processes [15], bimodal genes tend to cluster into synchronously expressed functional groups of «close neighbors». We described an approach for identification of such groups based on normalized gene expression, which makes it platform-independent and comparable between different arrays. We have selected 23 genes divided into 5 groups which were co-expressed within groups in all five studies on three different platforms (but the group expression was independent from one to another). The genes within the groups are functionally close. Thus, genes in each group form statistically significant protein interaction networks. Some groups, such as TCAP, PSMD3, GRB7, and ERBB2, belong to a well known amplicon [36]. Transcription of MX1, CXCL10, PLSCR1, and ISG15 from the STAT1 group is regulated by STAT1 [37,38]. 15 out of 23 bimodal genes in groups are known in the literature as breast cancer-associated genes, which suggests breast cancer specificity of these functional modules.

As gene expression within a group is synchronised through many studies, «group expression» can be applied as a «binary» conditional descriptor separating a patient cohort into sub-groups with «-1» and «1» expression. Consecutive application of different groups can be appled for further sub-division of the patient cohort into patient clusters, with "1s" and "2s" for each group used as a bar-code for the patent cluster. The advantage of using group expression instead of individual gene expression is in high robustness: an average per group expression fluctuates at a lower scale than dispersed expression of individual genes. The «close neighbors» gene groups can be used as prognostic descriptors for clinical end-points such as patient survival, metastases development, response to therapy, etc. Sub-categorization of cancer patients is a non-trivial problem due to high heterogeneity of expression profiles. Thus, in a well-known sub-categorisation scheme which divided invasive breast cancers into five clusters based on expression of certain "centroid" genes [28], over 1/3 of samples could not be categorized into any cluster and expression heterogeneity within clusters was still high, especially in validation studies with more samples, despite running the studies on essentially the same cDNA array [50,51]. Importantly, when we applied normalized group expression as the clustering metrics, we saw not a single outlier among over 1000 samples in five studies on three different microarray platform. The heterogeneity was also much lower within the clusters (data not shown). Such high robustness makes the "close neighbors" groups potentially very promising biomarkers for clinical end-points in breast cancer and, likely, other types of cancers.

Functional grouping of genes as descriptors also deals with an important issue of reduction of dimensionality in meta-analysis. Meta-analysis can be defined as a cross-study analysis of different patient cohorts united by a clinical end-point or any other parameter [24,52]. This type of analysis is broadly applied, for example, during comparison of a study of interest with the expression data accumulated in GEO http://www.ncbi.nlm.nih.gov/geo/ or other expression databases (ArrayExpress http://www.ebi.ac.uk/microarray-as/ae/, Stanford Microarray Database http://smd.stanford.edu/), Yale Microarray Database http://www.med.yale.edu/microarray/, etc). Platform compatibility and minimization of «dimensionality» are two major problems in meta-analysis, where «gene signatures» consisting of individual genes are notorious for poor reproducibility [40-44,23]. Here, we offer a general solution for the problem, consisting of identification of bimodal genes, normalization of their expression and grouping of the normalized expression into synchronised clusters of «close neighbors». Normalization consists of transformation of expression signals into a binary system of «-1» and «1», and it enables comparison of otherwise incomparable expression data between platforms and studies [53]. Lack of individual genes on a certain array platform does not prevent using the group as the descriptor.

## Conclusion

We described the phenomenon of bimodality of gene expression in breast cancer and grouping of the bimodal genes into «close neighbor» groups. The sets of bimodal genes are non-random; they are enriched in disease markers and targets and tend to form functionally related groups with synchronised expression. These groups of «close neighbors» can be used as robust descriptors for certain sub-groups of patients and associated with clinically important phenotypes (end-points). Application of functional descriptors consisting of bimodal genes is important in the area of meta-analysis of gene expression experiments across platforms and across studies.

## Methods

### Identfication of bimodal genes

In a set of expression experiments (for instance, a patient cohort), each gene has a distribution of expression signals across the set. Bimodal genes feature a distribution with two distinct peaks (maximal signals) (Fig 1B). For each gene, we can set up a distinguishing expression value such that the signals lower than this value correspond to the lower peak in the bimodal distribution, and the signals higher than this value correspond to the higher peak. The characteristic value was chosen as follows: all expression values for a gene were randomly divided onto two groups, and average and sum of squared deviations were calculated for each group. The lower the sum of deviations, the better the partition.

In calculation of bimodality, we assume that distribution of expression within the cohort for a bimodal gene is a sum of two normal distributions. Let us consider  - an expression value of *i*-s gene in the *j*-s experiment; *L*_*i*_, *U*_*i *_- partition of the set of all experiments onto subsets depending on *i*-s gene); #*L*_*i*_, #*U*_*i *_- the number of experiments in each subset;  - average signal for *i*-s gene in each subset *L*_*i*_, *U*_*i*_. We need to find a partition with the minimum *γ*(*L*_*i*_, *U*_*i*_): . We need to look at only the subsets with  - the values in subset *L*_*i *_are lower than in subset *U*_*i*_. The number of possible partitions with such a property is larger by 1 than the number of experiments (including two cases with empty subsets). For the «optimal» partition, *l*_*i *_= ⟨*L*_*i*_⟩, *u*_*i *_= ⟨*U*_*i*_⟩ and *γ*_*i *_= *γ*(*L*_*i*_, *U*_*i*_). In this case, the characteristic signal *T*_*i *_will be calculated as follows:

. In other words, the characteristic signal *T *is the border with two optimal divisions, i.e.  (Figure 2A).

We can consider as the level of bimodality a relative dicrepancy in the values in sub-sets (measure of signal isolation) *τ*_*i*_, which is calculated as , where *M *- is the total number of experiments.

Finding the peaks is carried out by the following procedure:

1. The list of values for *i-*s gene is entered into an algorithm

2. All signals are sorted by value: the number of signals is *n*, where *j *is the number of the signal

3. For all *n *- 1 possible partitions of the sorted list of values onto two groups (partition is defined by the number of the highest signal in the smaller by value group), Partition is defined as *k *∈ [1, *n *- 1]):

1. The average for each sub-set is  for the sub-set with lower signals  - for the sub-set with higher signals

2. The sum of squares of deviations for each sub-set: 

4. We choose *K*_*i*_, for which  is ()

5. The algorithm results in *K*_*i*_, , .

where  divides the signals for the given partition according to . This property allows us to clearly divide signals for each peak (mode).

### Outliers

One of the drawbacks of the method described above is its sensitivity to outliers. For instance, if in three experiments out of 100 the expression values are significantly higher than the others, the three signals will be assigned to one peak, and the remaining 97 to another peak. This situation can be avoided if all values for a group will be considered as outliers if its relative size is small, for instance, less than 5%.

### Bimodal normalization

We consider as normalization a linear transformation of signals  so that:

This transformation allows us to reduce all signals *l*_*i *_and *u*_*i *_to -1 and 1, correspondingly (Figure 2B). If the set contains a certain number of control experiments (for instance, normal samples among the disease samples), we can consider the expression values for the group with normal samples as 1, and the other group as -1. This allows us to compare expression profiles which are synchronised among the patients but in different directions. Also, the genes with control values belonging to different modes can be excluded.

• The mean for normal patients was calculated , where *N *- samples from normal patients, and #*N *is their number

• In the case of *ν*_*i *_<*T*_*i*_, the gene expression values were transformed by ; otherwise . Therefore,  always.

### Selection of the groups of synchronously expressed genes

**Figure 7:**
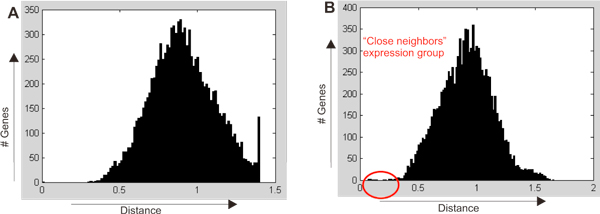
**Identification of the "close" groups of genes in the space of 295 samples (Sorlie295 data set)**. (A) No close group is found for HMGA1 as query gene. OX: relative distances from the query gene to all 10604 array genes. OY: the number of genes. (B) Clear close group around ERBB2/GRB7 (encircled). OX: relative distances from the query gene to all 10604 array genes. OY: the number of genes.

For all the bimodal genes with normalized expression, we can search for genes expressed in a similar manner. For each gene, we calculated the cosine distance to all other genes as:

The outliers to 0 were added to the candidate genes (Figure 7). This method allows us to identify groups of genes with similar expression profiles within the group and sufficiently different from other genes.

The genes with similar signal profiling constitute a group of «close neighbors»

## Competing interests

The authors declare that they have no competing interests.

## Authors' contributions

Marina Bessarabova - writing the manuscript, data analysis; Eugine Kirillov - calculation of bimodal genes, groups, normalization; Weiwei She - statistical analysis; Andrej Bugrim - functional analysis; Yuri Nikolsky - data analysis, editing manuscript; Tatiana Nikolskaya - scientific leader; original study design.

## Supplementary Material

Additional file 1 **The list of bimodal genes (866)**. Worksheet 1. Data pre-processing. Describes pre-processing workflow for each data set. Worksheet 2. Bimodal genes. Includes list of 866 bimodal genes (see Results). Click here for file

Additional file 2 **Raw data graphs**. Expression profiles for bimodal genes in 5 data sets before normalization. Graphs. Click here for file

Additional file 3 **Raw data box plots**. Expression profiles for bimodal genes in 5 data sets before normalization. Box Plots Click here for file

Additional file 4 **Normalized data graphs**. Expression profiles for bimodal genes in 5 data sets after normalization. Graphs Click here for file

Additional file 5 **Normalized data box plots**. Expression profiles for bimodal genes in 5 data sets after normalization. Box Plots Click here for file

Additional file 6 **"Close neighbors" expression groups of bimodal genes splits patients into groups**. Click here for file
