# Supplementary material for: Bimodal gene expression patterns in breast cancer
Source: BMC Genomics. 2010 Feb 10;11(Suppl 1):S8. doi: 10.1186/1471-2164-11-S1-S8 (PMC2822536; doi:10.1186/1471-2164-11-S1-S8)
Supplement: Additional file 2 — Raw data graphs. Expression profiles for bimodal genes in 5 data sets before normalization. Graphs. [file 1471-2164-11-S1-S8-S2.pdf]

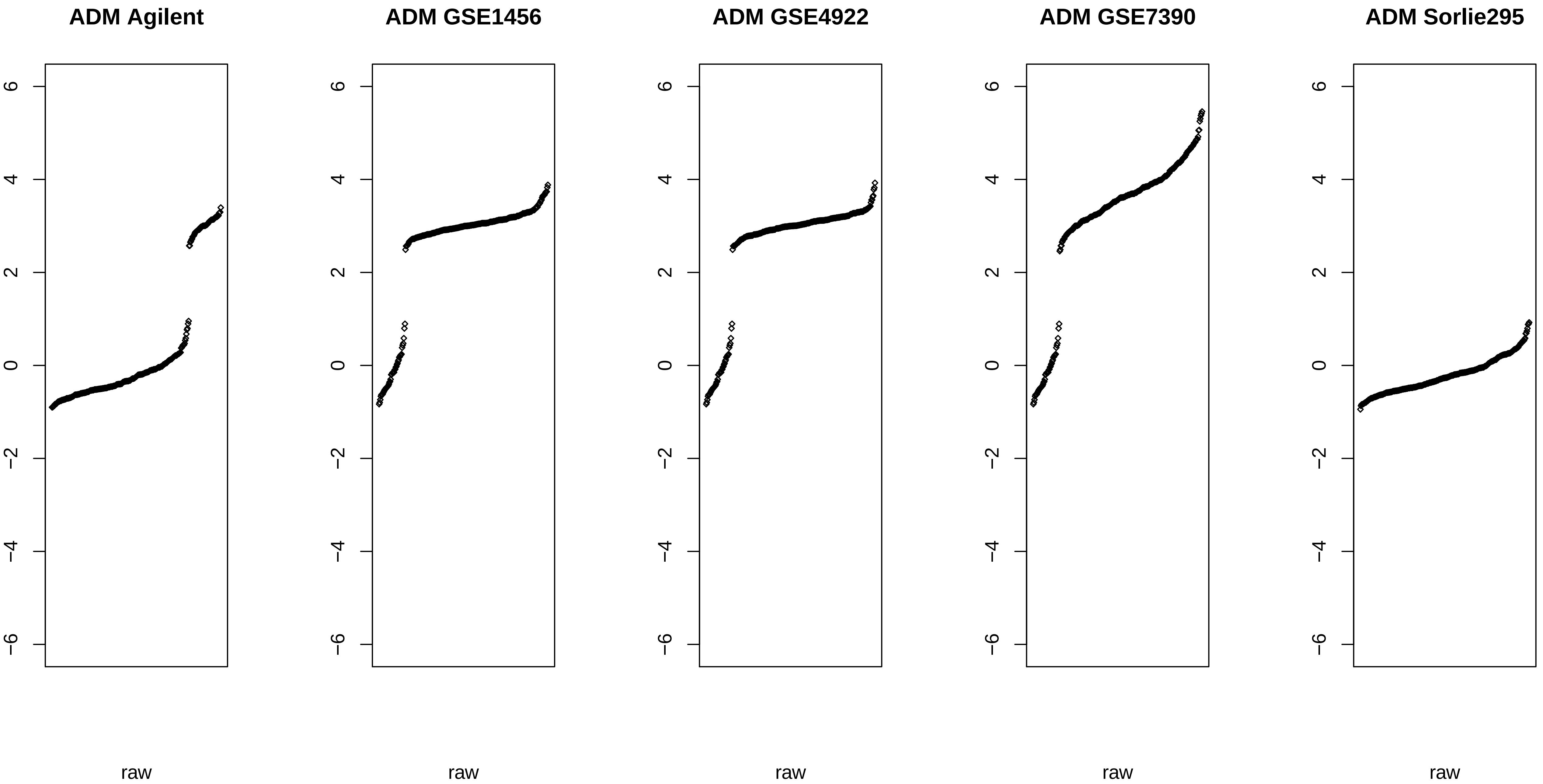
ADM Agilent
ADM GSE1456
ADM GSE4922
ADM GSE7390
ADM Sorlie295
6
4
2
0
−2
−4
−6
raw

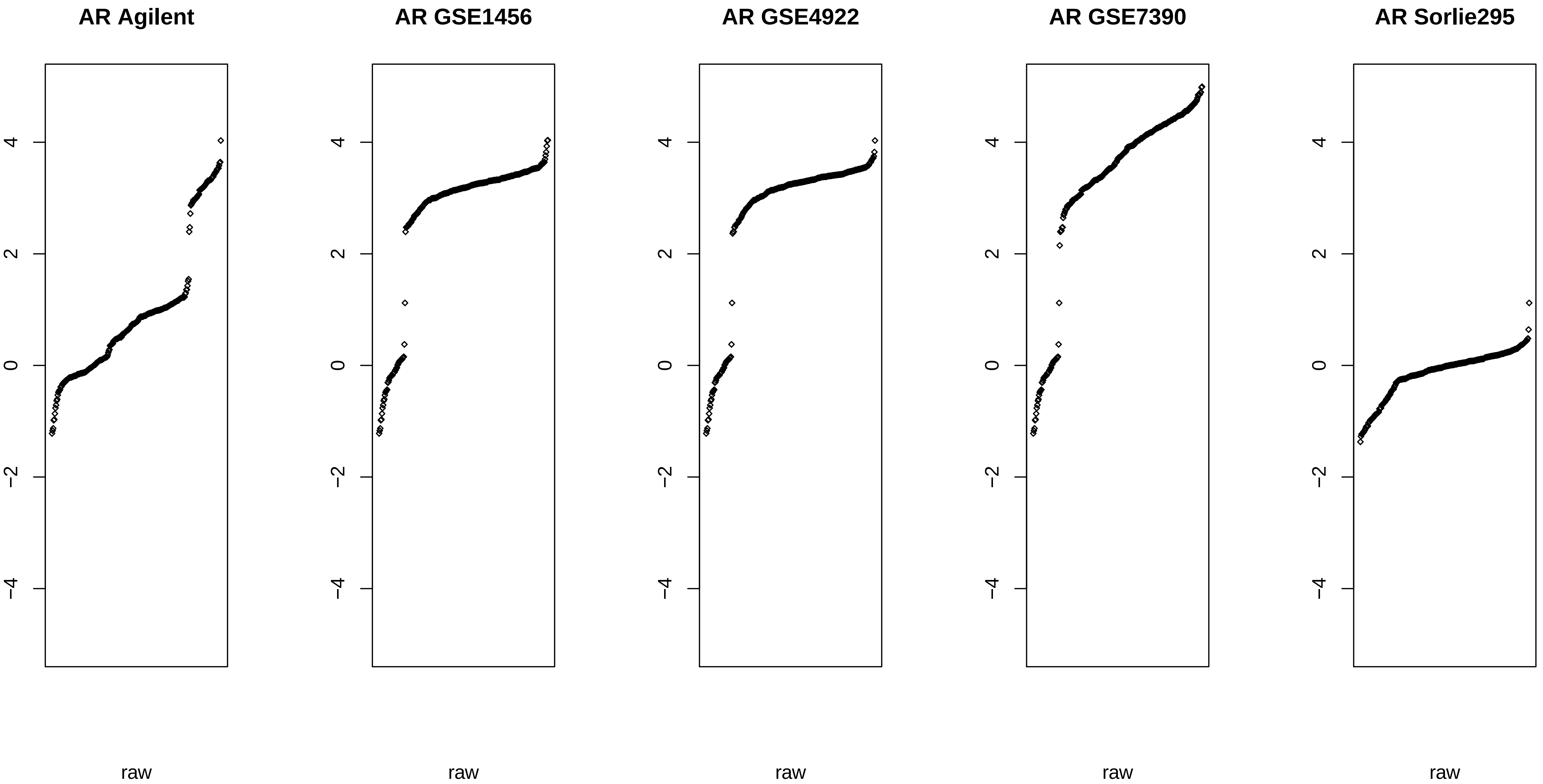

AR Agilent
AR GSE1456
AR GSE4922
AR GSE7390
AR Sorlie295
4
2
0
−2
−4
raw

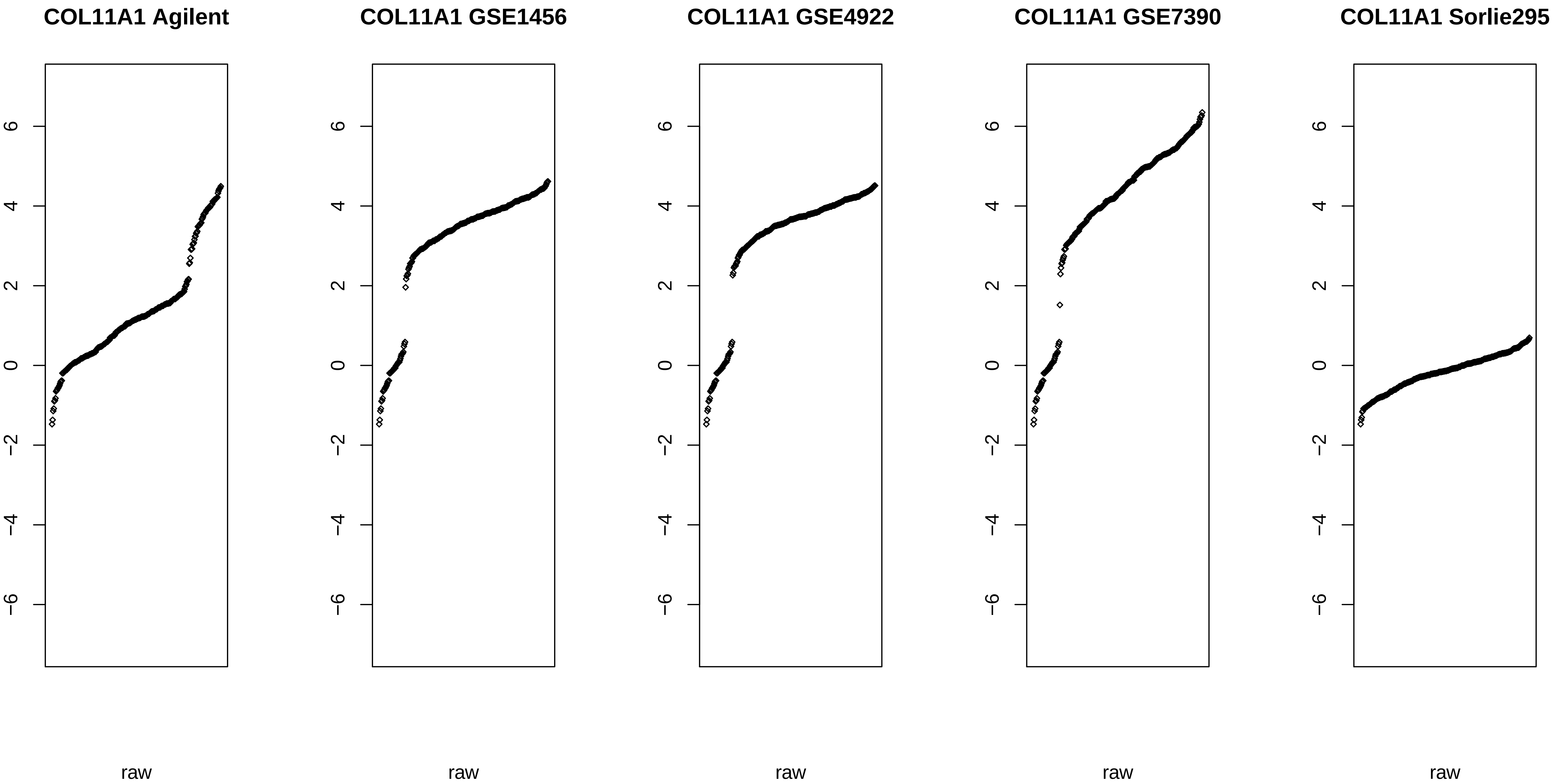

COL11A1 Agilent
COL11A1 GSE1456
COL11A1 GSE4922
COL11A1 GSE7390
COL11A1 Sorlie295
6
4
2
0
−2
−4
−6
raw

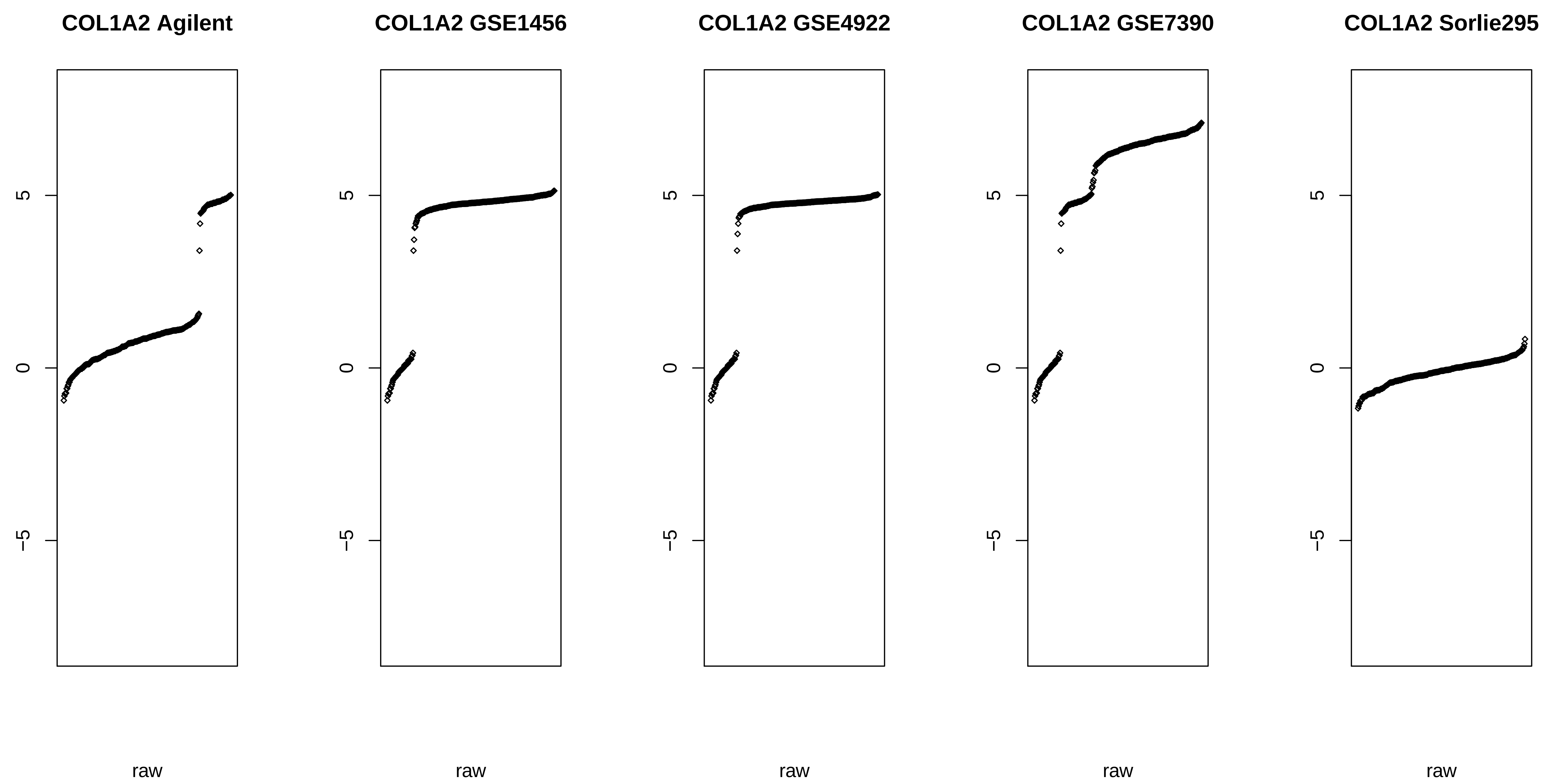
COL1A2 Agilent
COL1A2 GSE1456
COL1A2 GSE4922
COL1A2 GSE7390
COL1A2 Sorlie295
5
0
−5
raw

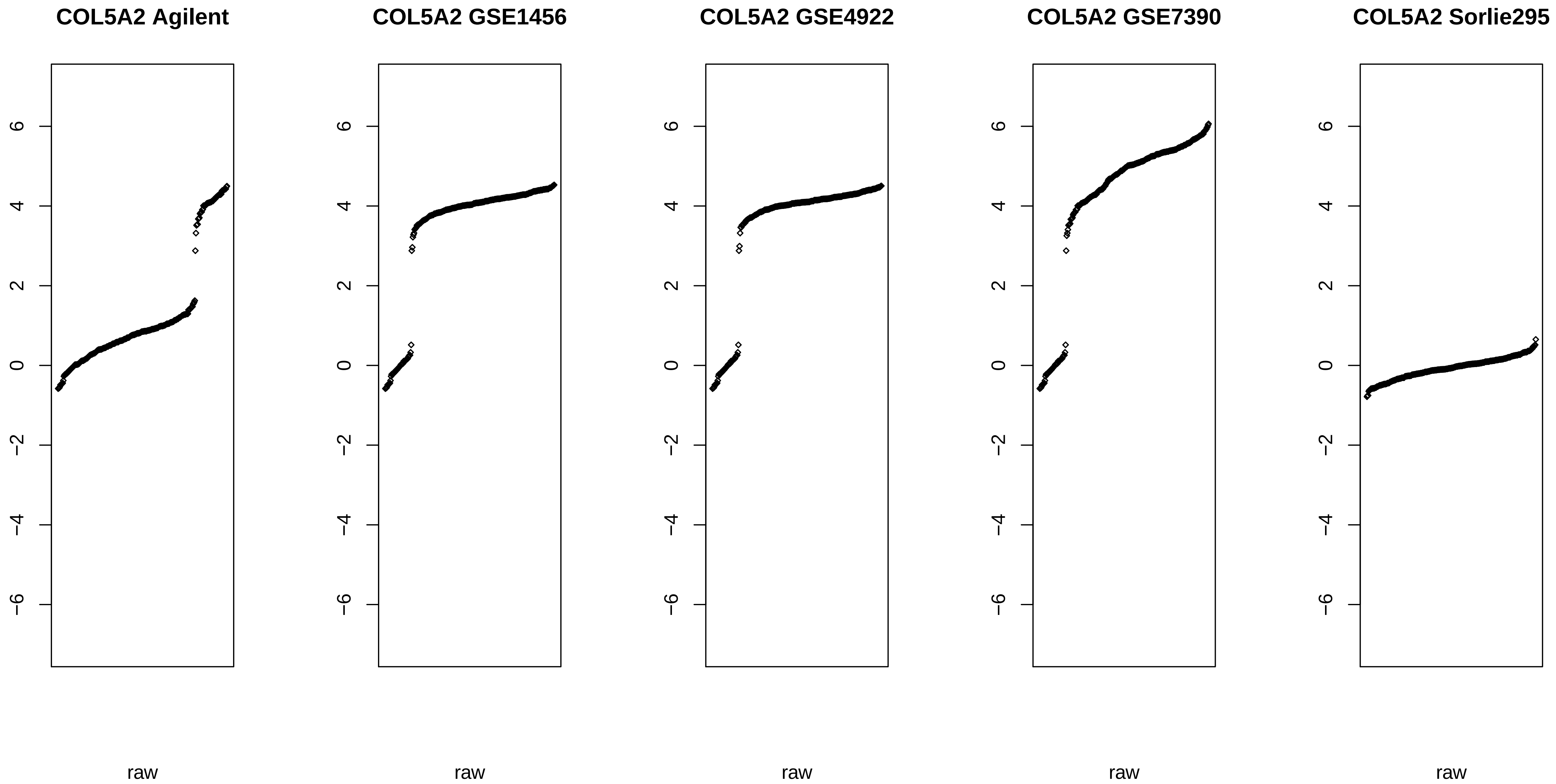
COL5A2 Agilent
COL5A2 GSE1456
COL5A2 GSE4922
COL5A2 GSE7390
COL5A2 Sorlie295
6
4
2
0
−2
−4
−6
raw
raw
raw
raw
raw

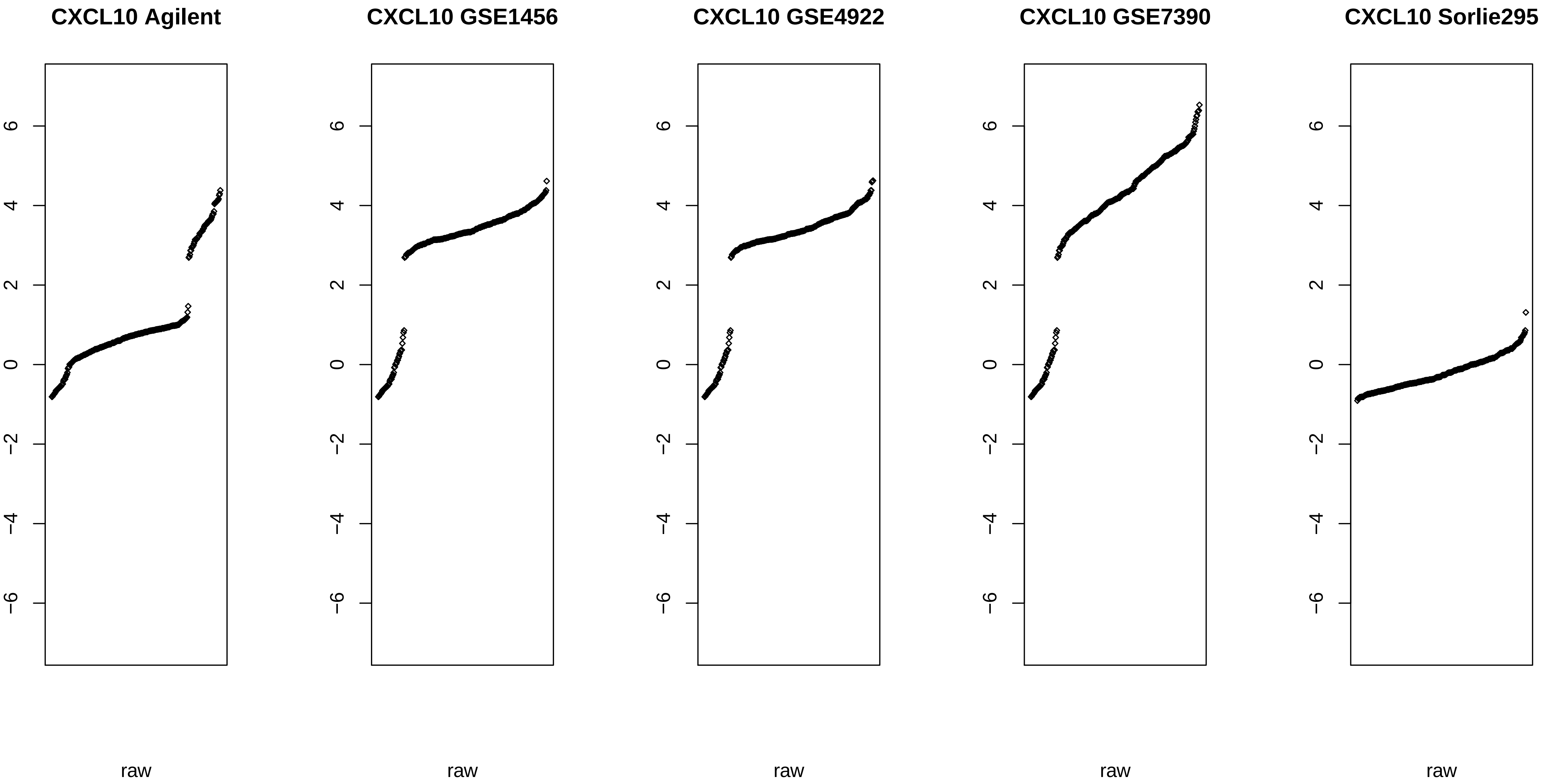
CXCL10 Agilent
CXCL10 GSE1456
CXCL10 GSE4922
CXCL10 GSE7390
CXCL10 Sorlie295
6
4
2
0
−2
−4
−6
raw

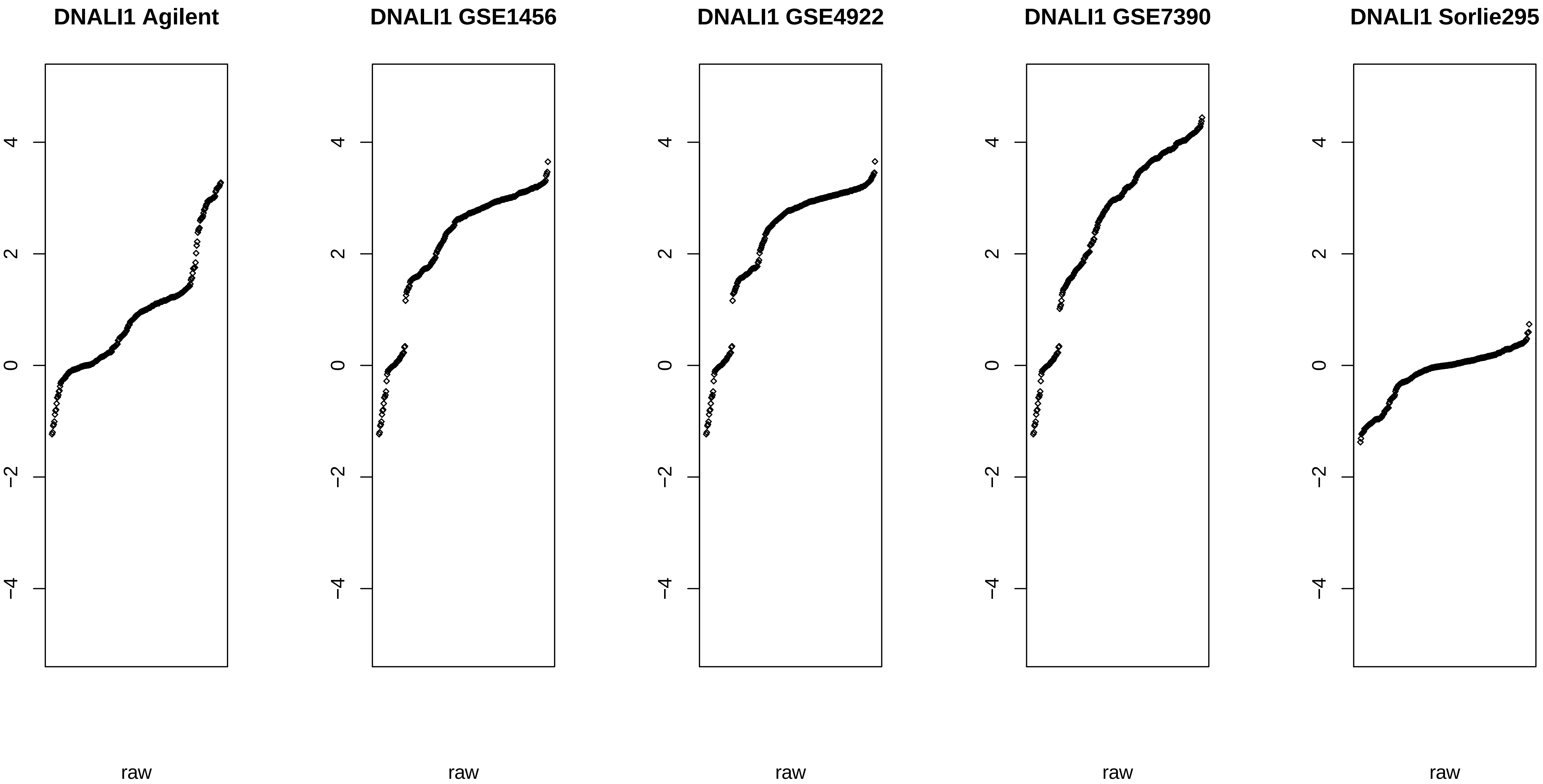

DNALI1 Agilent
DNALI1 GSE1456
DNALI1 GSE4922
DNALI1 GSE7390
DNALI1 Sorlie295
4
2
0
−2
−4
raw
raw
raw
raw
raw

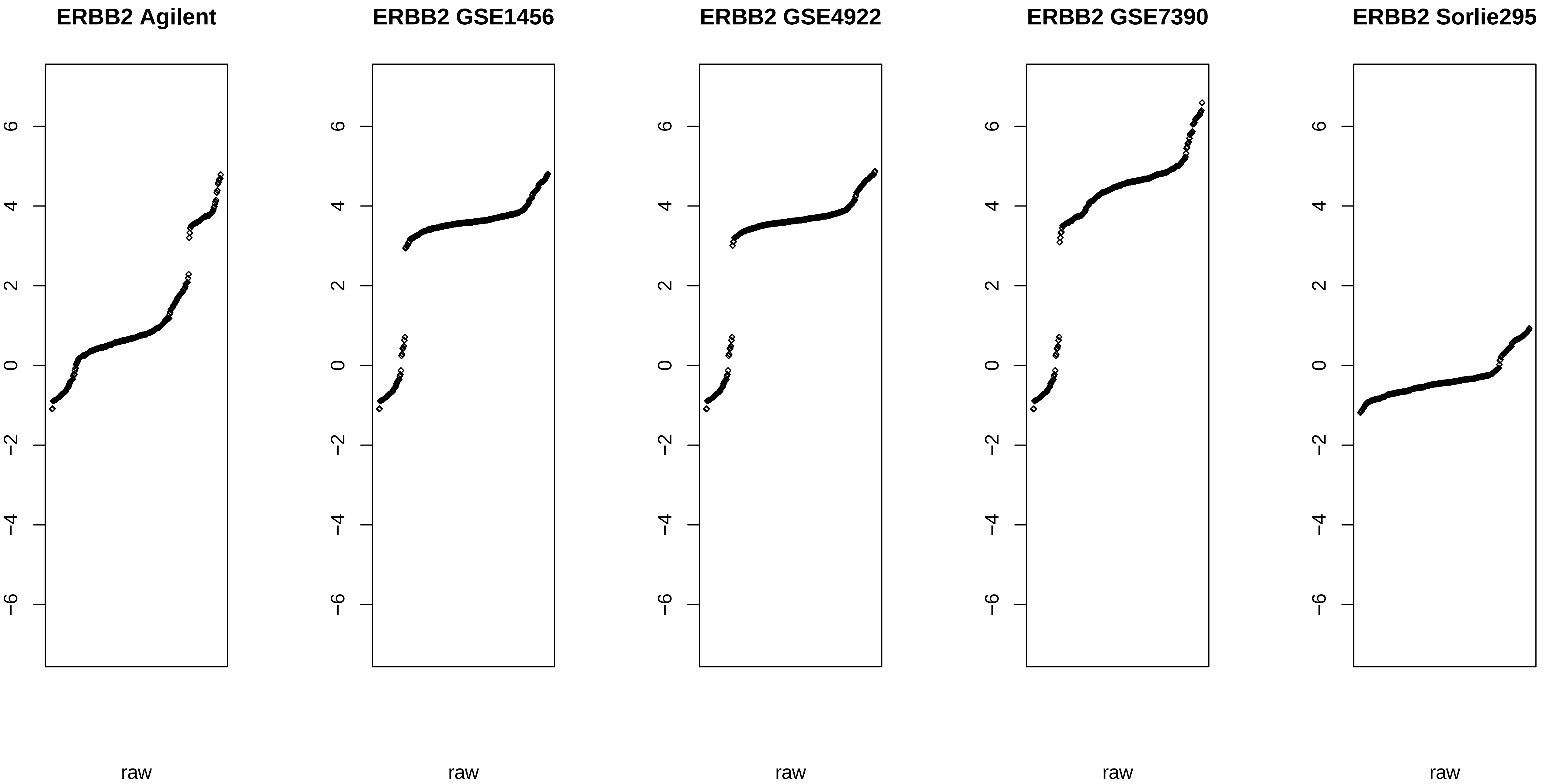
ERBB2 Agilent
ERBB2 GSE1456
ERBB2 GSE4922
ERBB2 GSE7390
ERBB2 Sorlie295
6
4
2
0
−2
−4
−6
raw

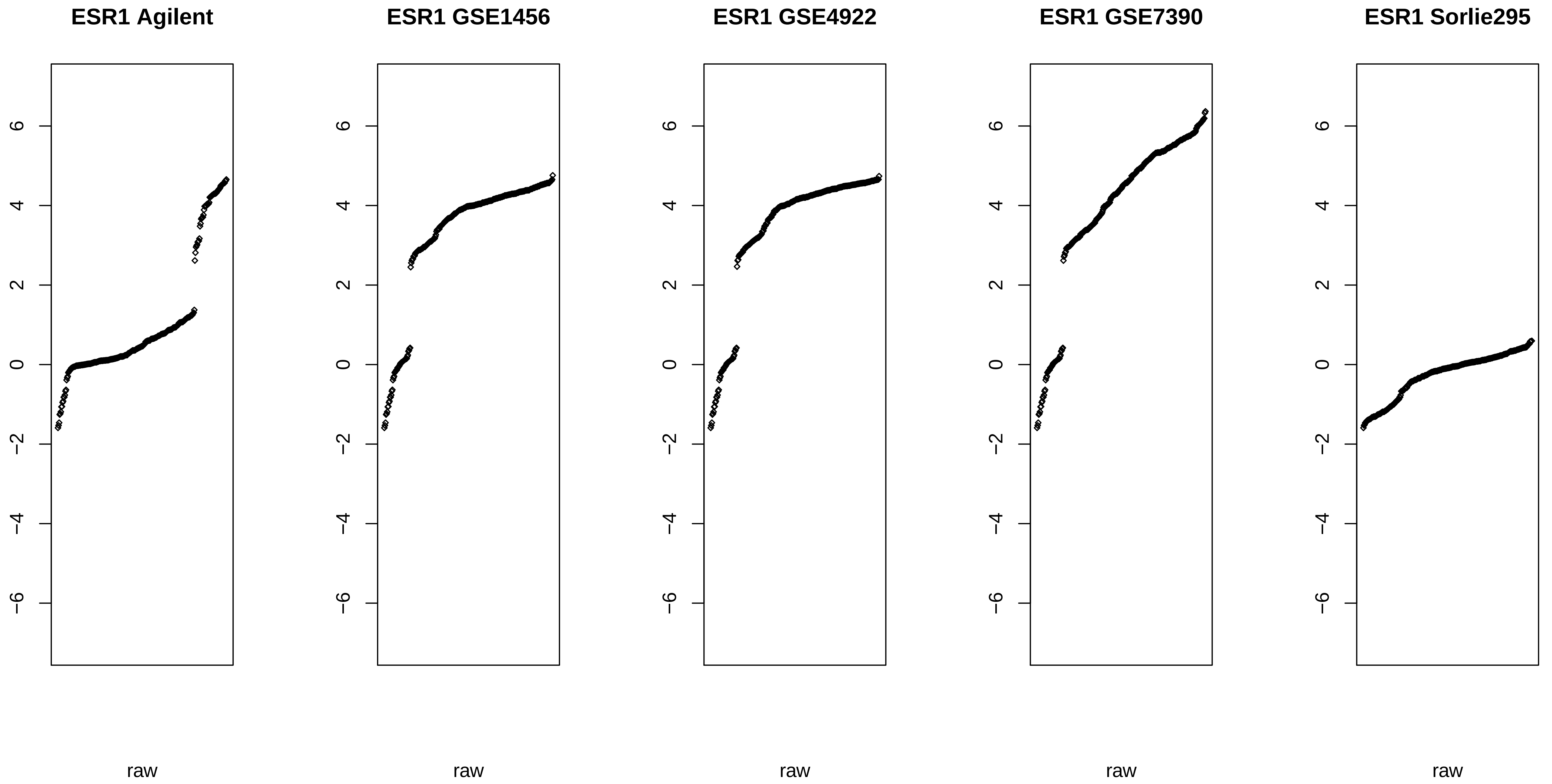
ESR1 Agilent
ESR1 GSE1456
ESR1 GSE4922
ESR1 GSE7390
ESR1 Sorlie295
6
4
2
0
−2
−4
−6
raw

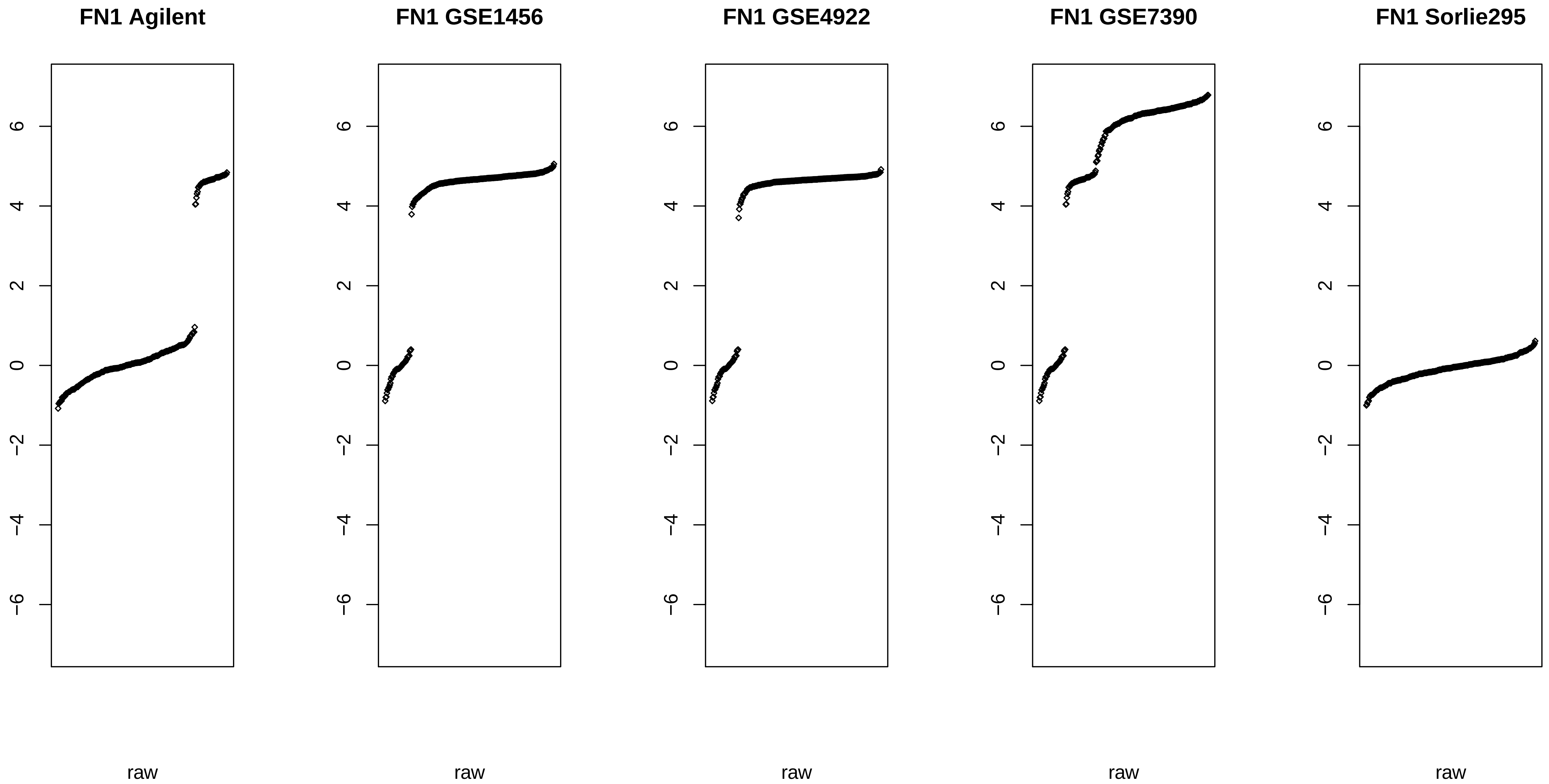
FN1 Agilent
FN1 GSE1456
FN1 GSE4922
FN1 GSE7390
FN1 Sorlie295
6
4
2
0
−2
−4
−6
raw

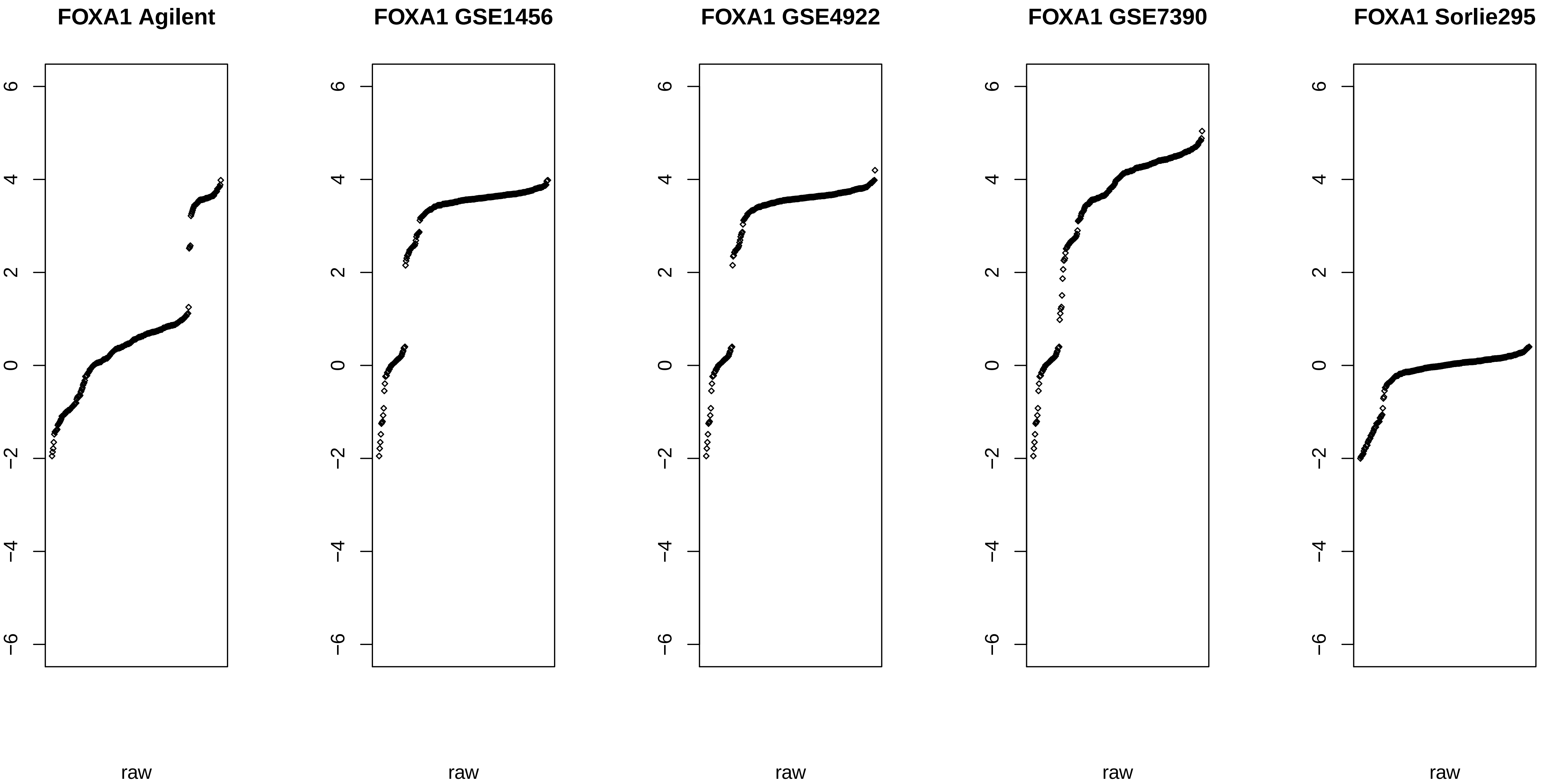

FOXA1 Agilent
FOXA1 GSE1456
FOXA1 GSE4922
FOXA1 GSE7390
FOXA1 Sorlie295
6
4
2
0
−2
−4
−6
raw
raw
raw
raw
raw

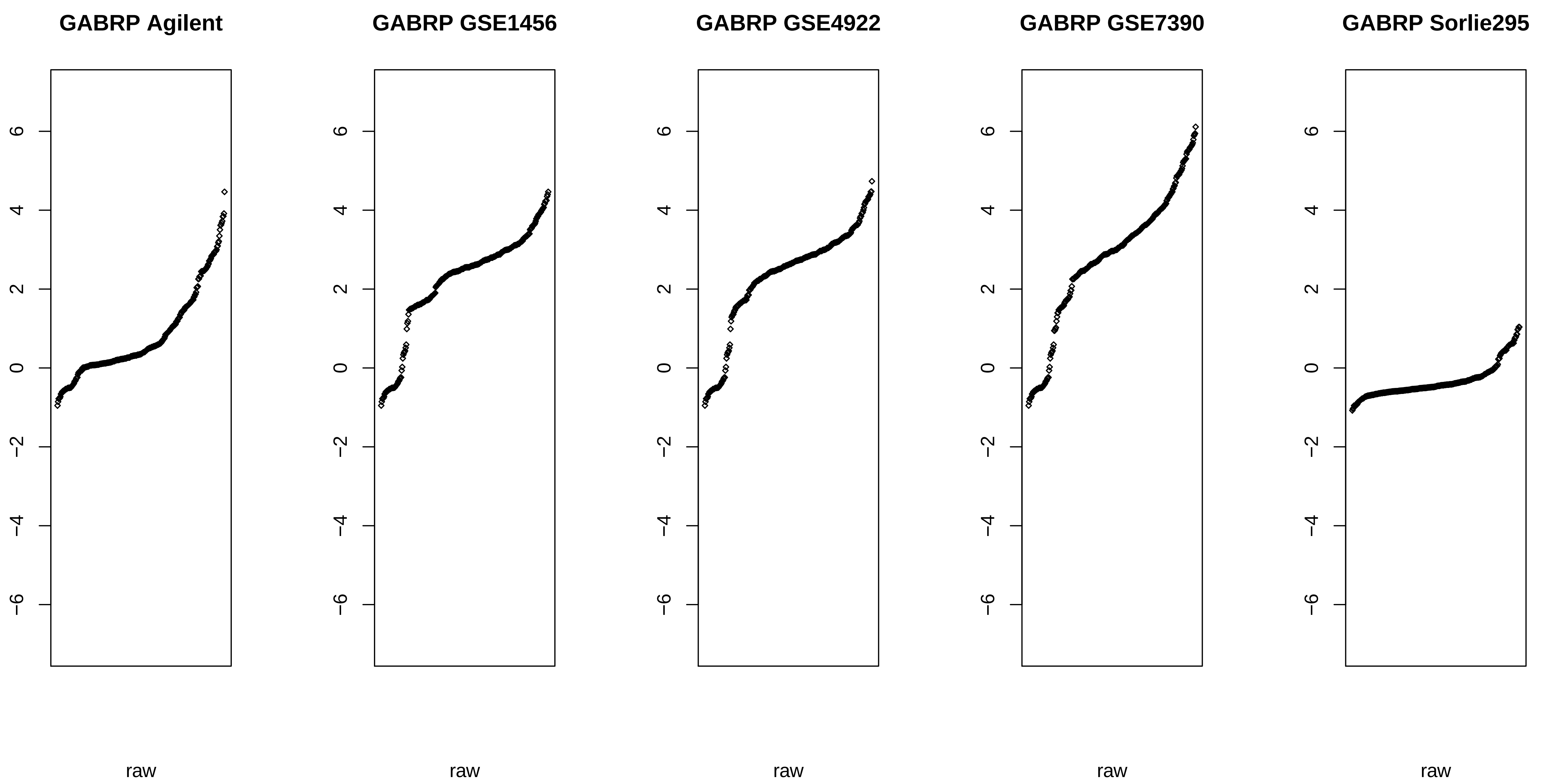
GABRP Agilent
GABRP GSE1456
GABRP GSE4922
GABRP GSE7390
GABRP Sorlie295
6
4
2
0
−2
−4
−6
raw
raw
raw
raw
raw

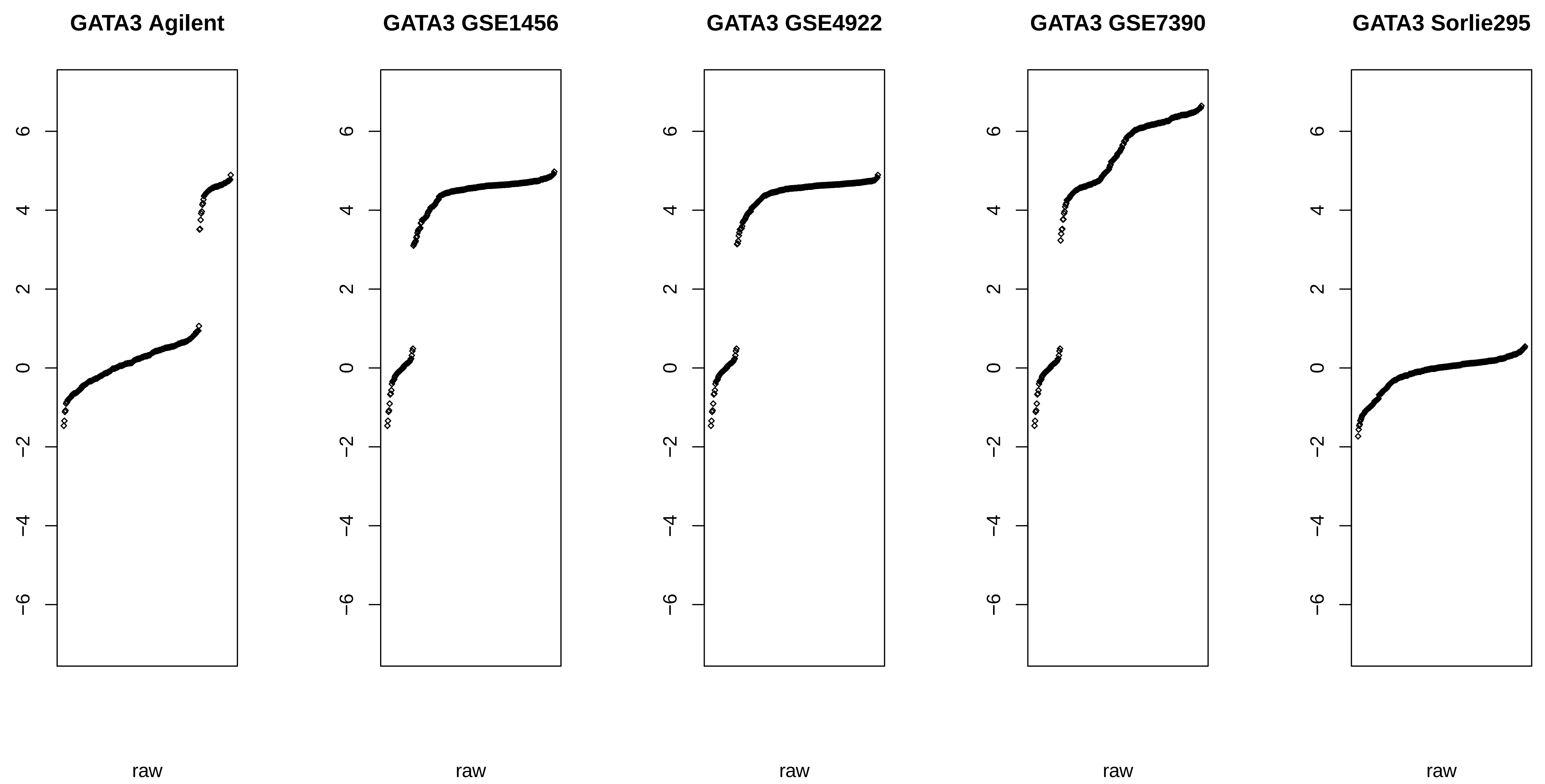
GATA3 Agilent
GATA3 GSE1456
GATA3 GSE4922
GATA3 GSE7390
GATA3 Sorlie295
6
4
2
0
−2
−4
−6
raw
raw
raw
raw
raw

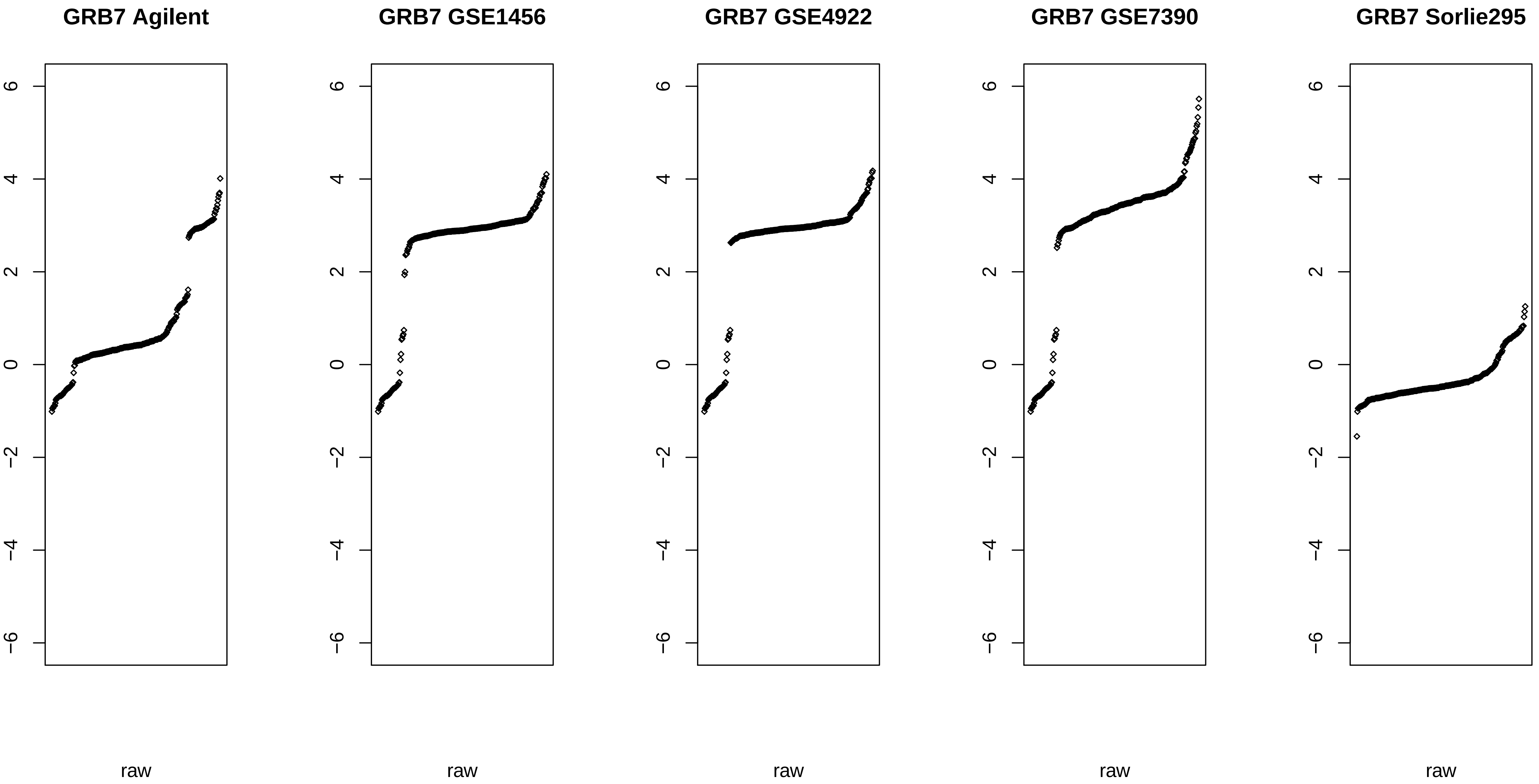
GRB7 Agilent
GRB7 GSE1456
GRB7 GSE4922
GRB7 GSE7390
GRB7 Sorlie295
6
4
2
0
−2
−4
−6
raw

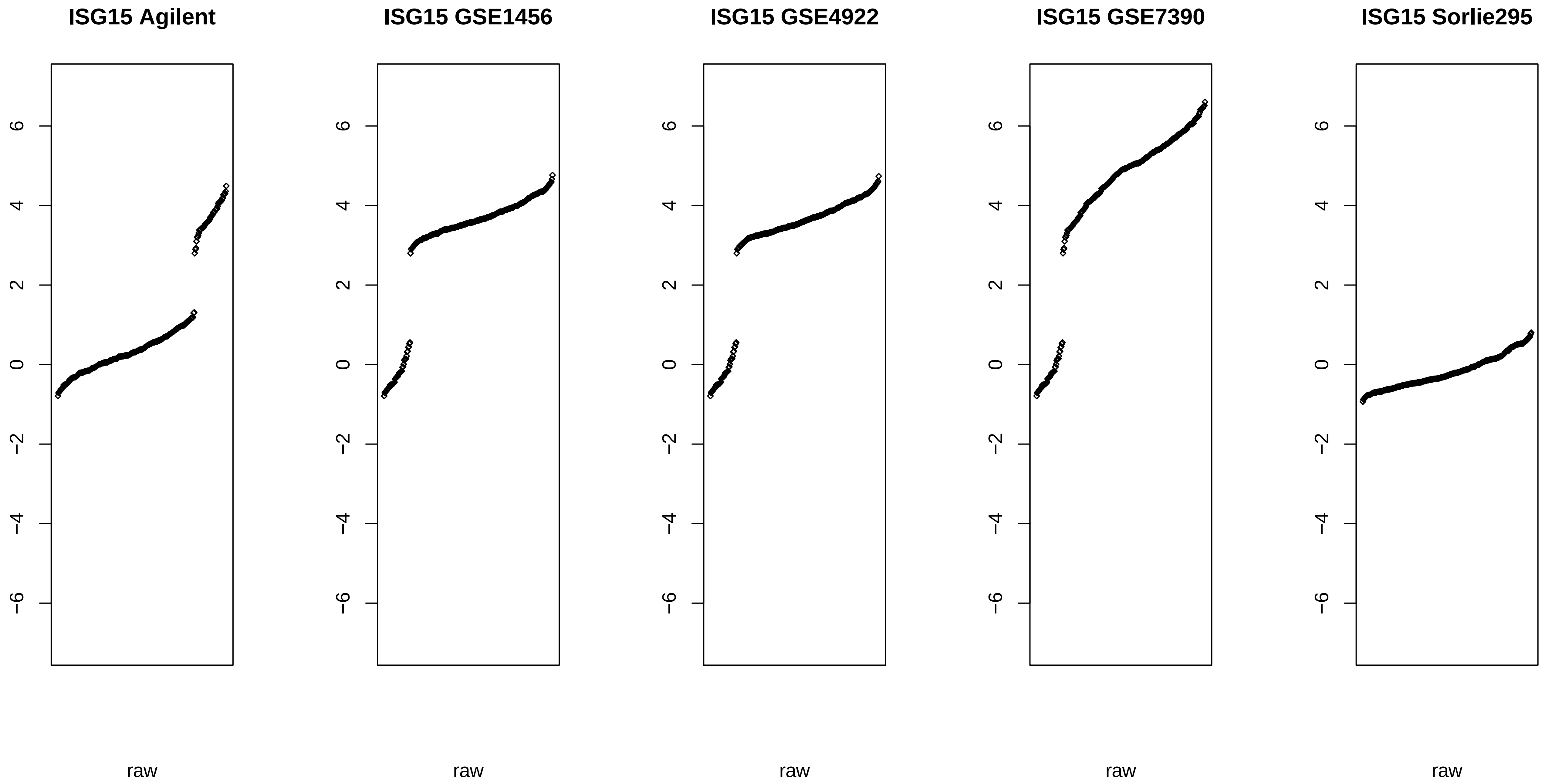
ISG15 Agilent
ISG15 GSE1456
ISG15 GSE4922
ISG15 GSE7390
ISG15 Sorlie295
6
4
2
0
−2
−4
−6
raw

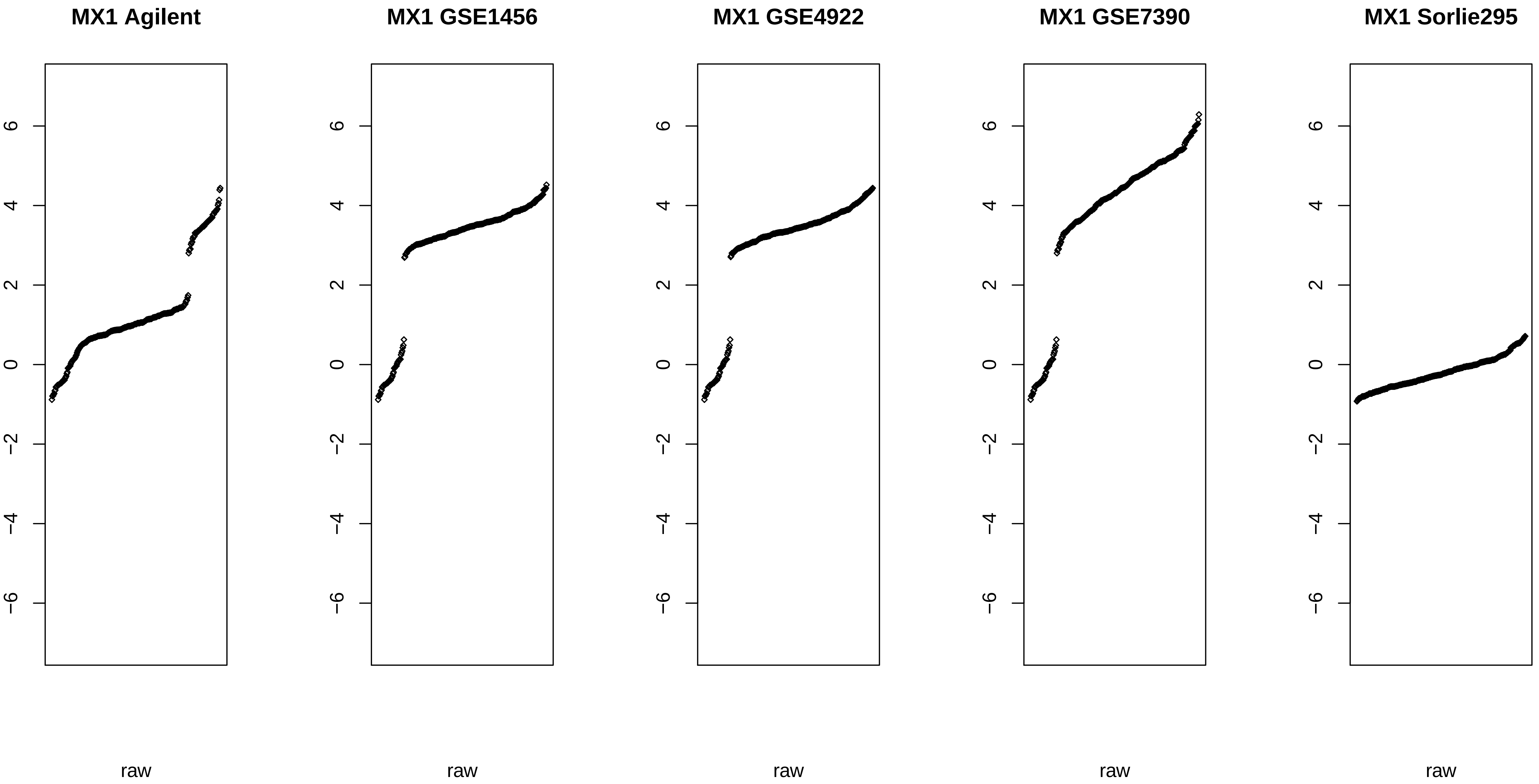
MX1 Agilent
MX1 GSE1456
MX1 GSE4922
MX1 GSE7390
MX1 Sorlie295
6
4
2
0
−2
−4
−6
raw

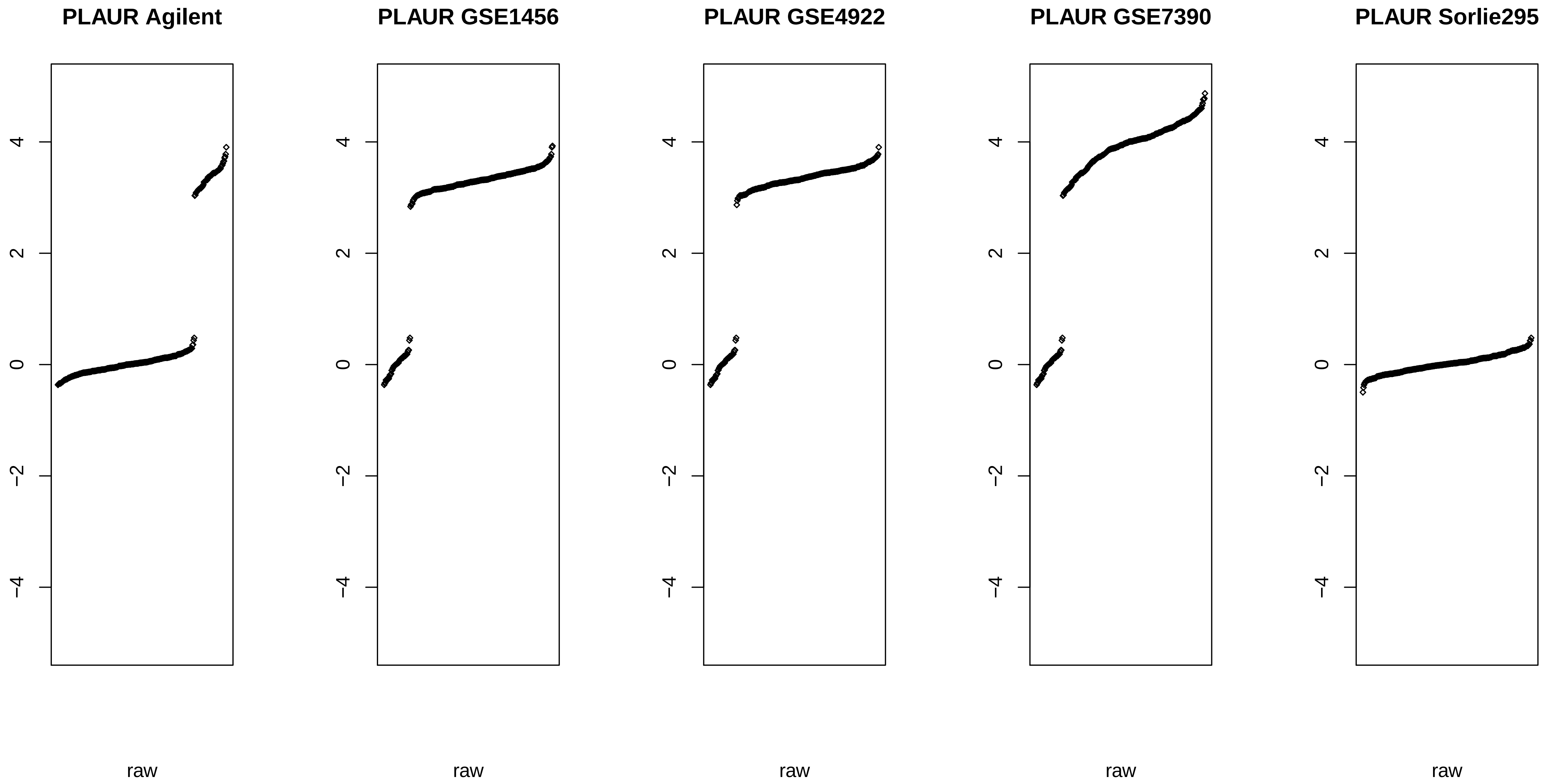
PLAUR Agilent
PLAUR GSE1456
PLAUR GSE4922
PLAUR GSE7390
PLAUR Sorlie295
4
2
0
−2
−4
raw

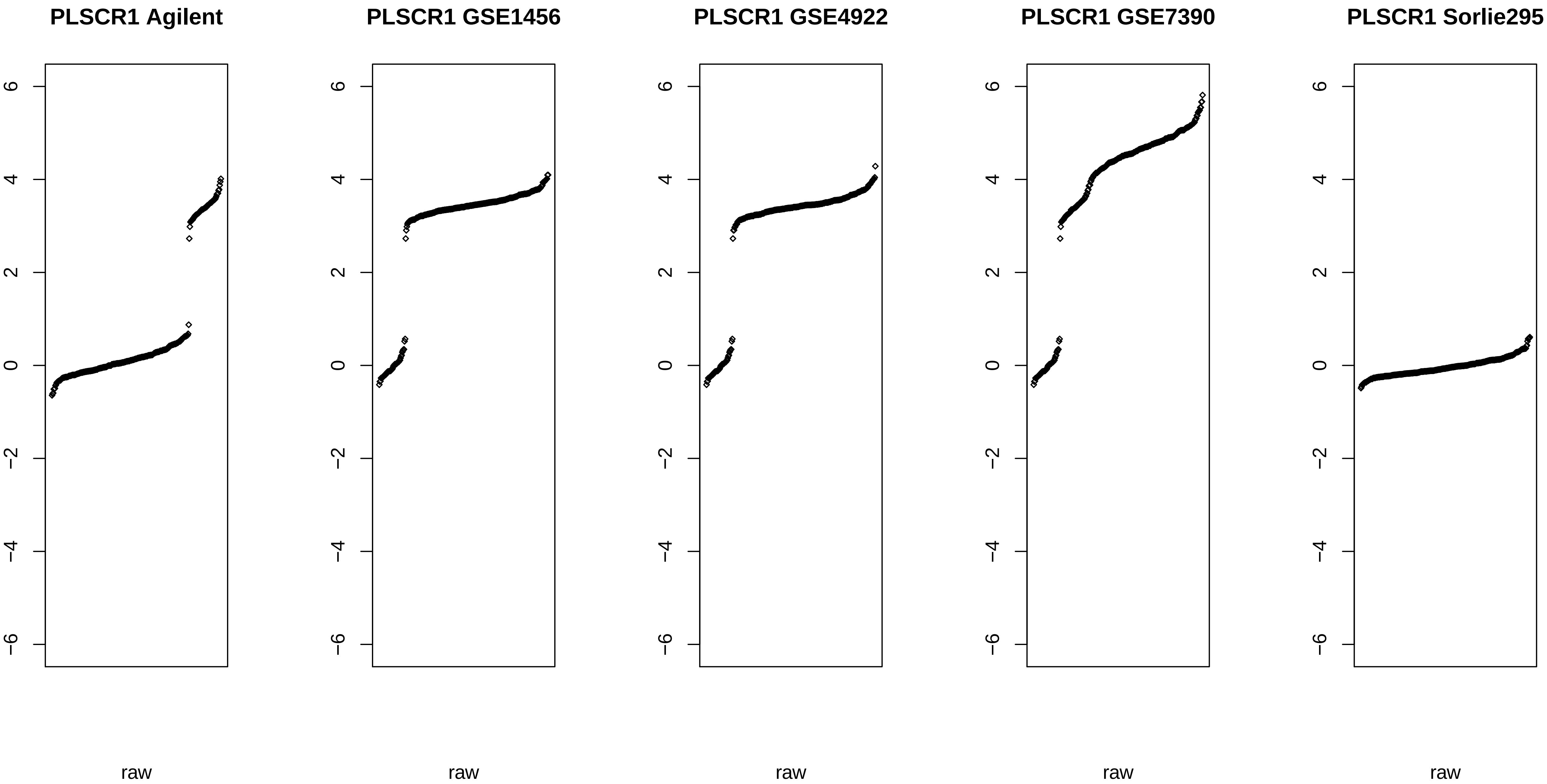
PLSCR1 Agilent
PLSCR1 GSE1456
PLSCR1 GSE4922
PLSCR1 GSE7390
PLSCR1 Sorlie295
6
4
2
0
−2
−4
−6
raw

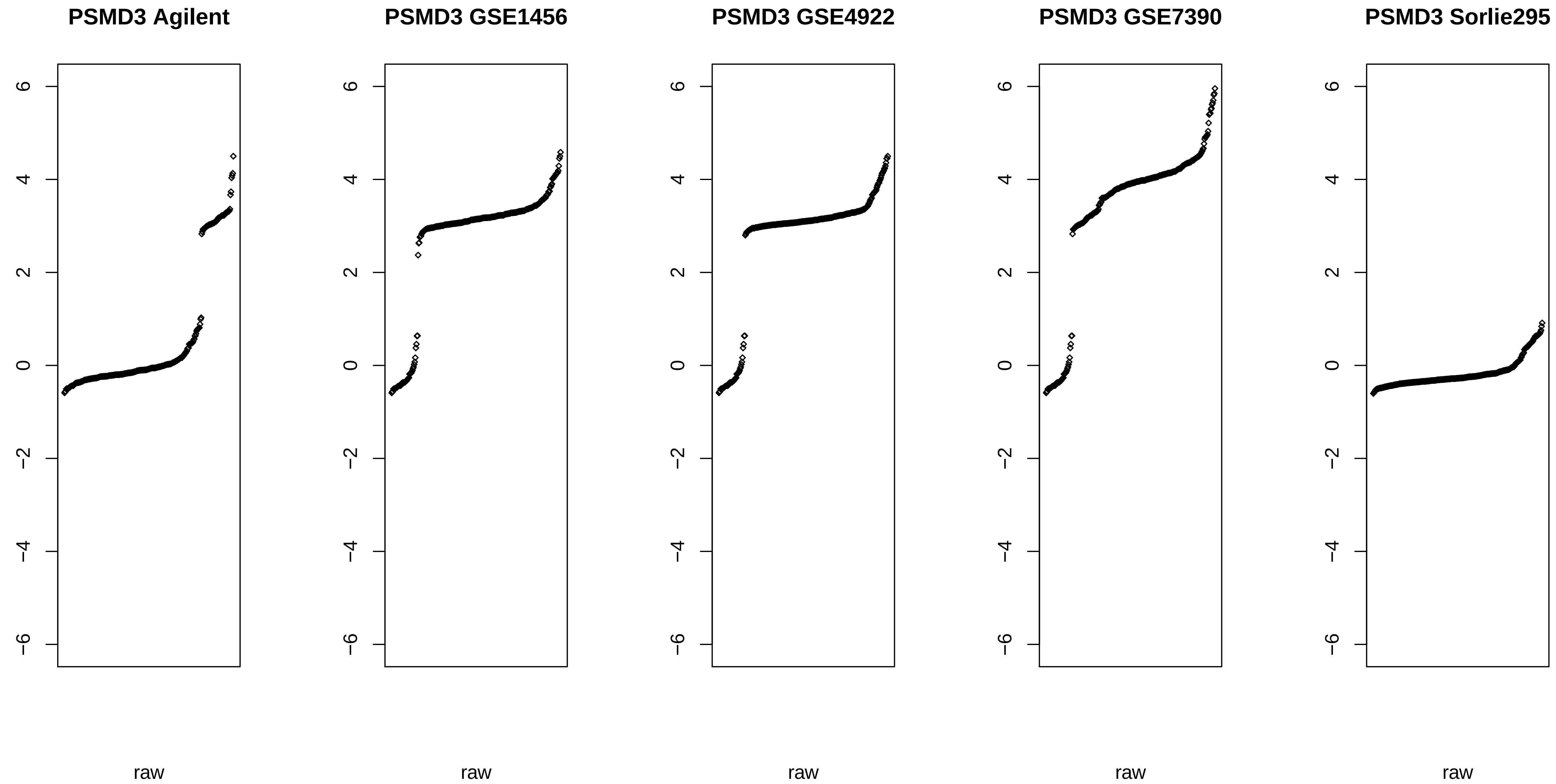
PSMD3 Agilent
PSMD3 GSE1456
PSMD3 GSE4922
PSMD3 GSE7390
PSMD3 Sorlie295
6
4
2
0
−2
−4
−6
raw

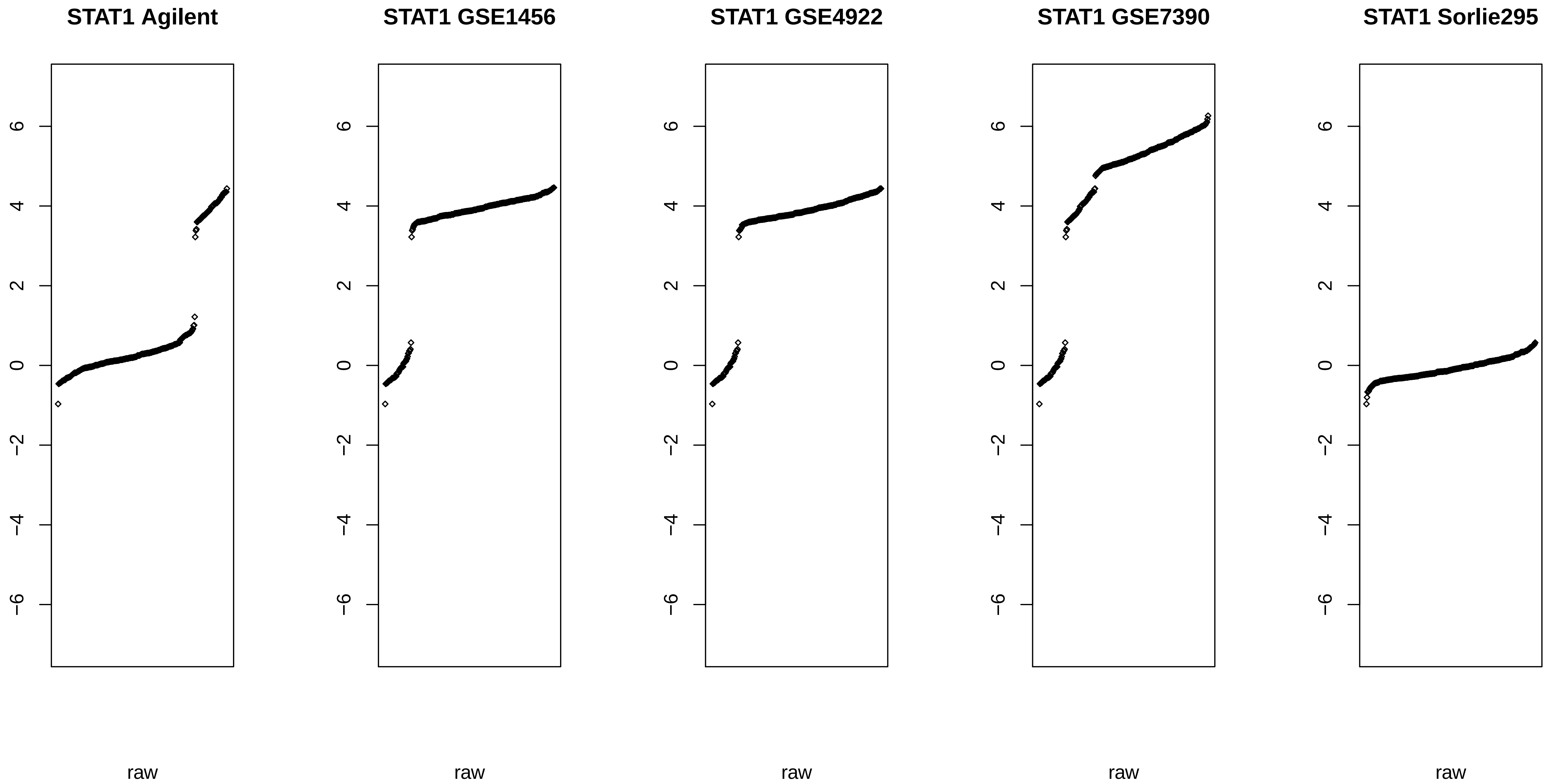
STAT1 Agilent
STAT1 GSE1456
STAT1 GSE4922
STAT1 GSE7390
STAT1 Sorlie295
6
4
2
0
−2
−4
−6
raw
raw
raw
raw
raw

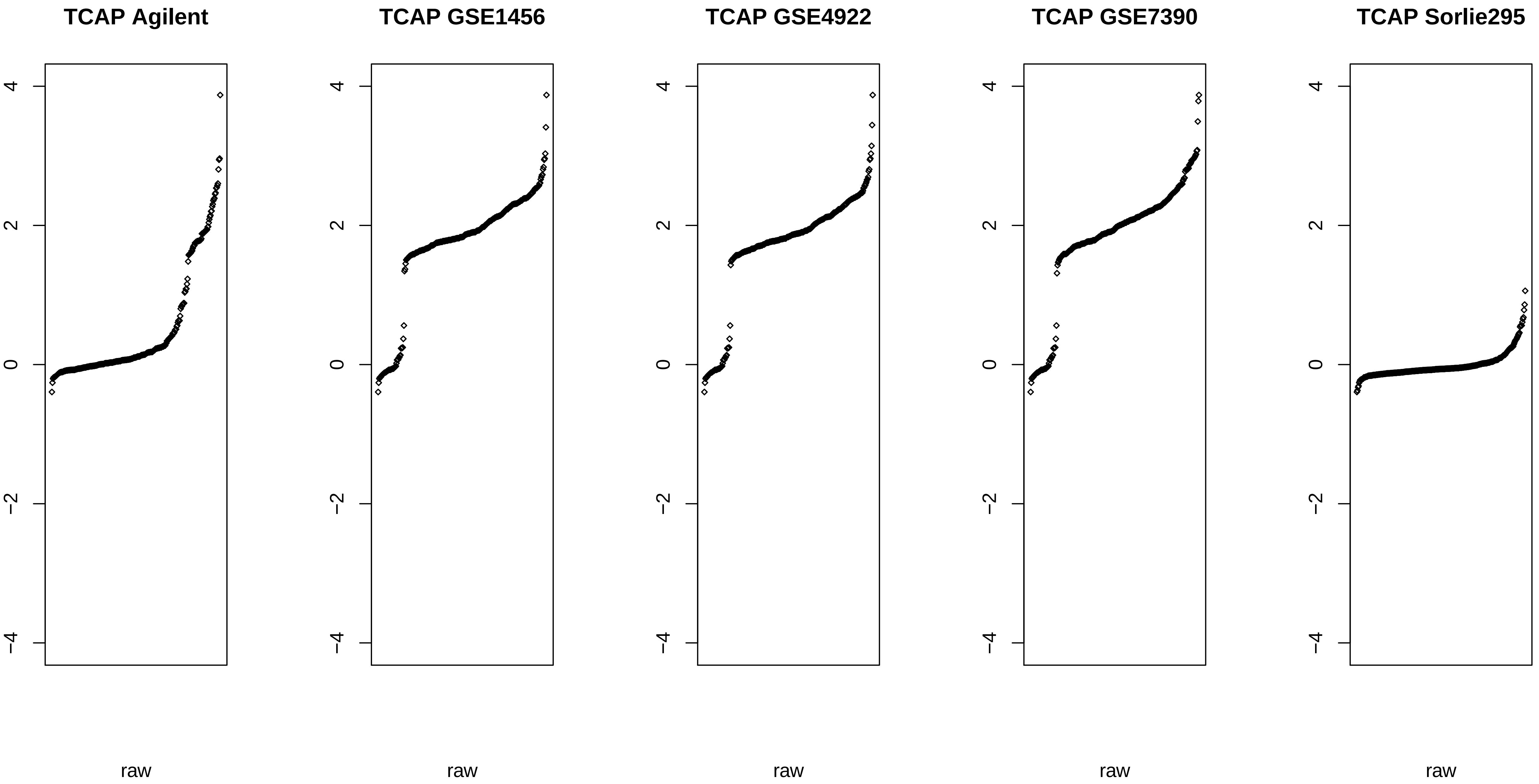

TCAP Agilent
TCAP GSE1456
TCAP GSE4922
TCAP GSE7390
TCAP Sorlie295
4
2
0
−2
−4
raw
