# Supplementary material for: Bimodal gene expression patterns in breast cancer
Source: BMC Genomics. 2010 Feb 10;11(Suppl 1):S8. doi: 10.1186/1471-2164-11-S1-S8 (PMC2822536; doi:10.1186/1471-2164-11-S1-S8)
Supplement: Additional file 3 — Raw data box plots. Expression profiles for bimodal genes in 5 data sets before normalization. Box Plots [file 1471-2164-11-S1-S8-S3.pdf]

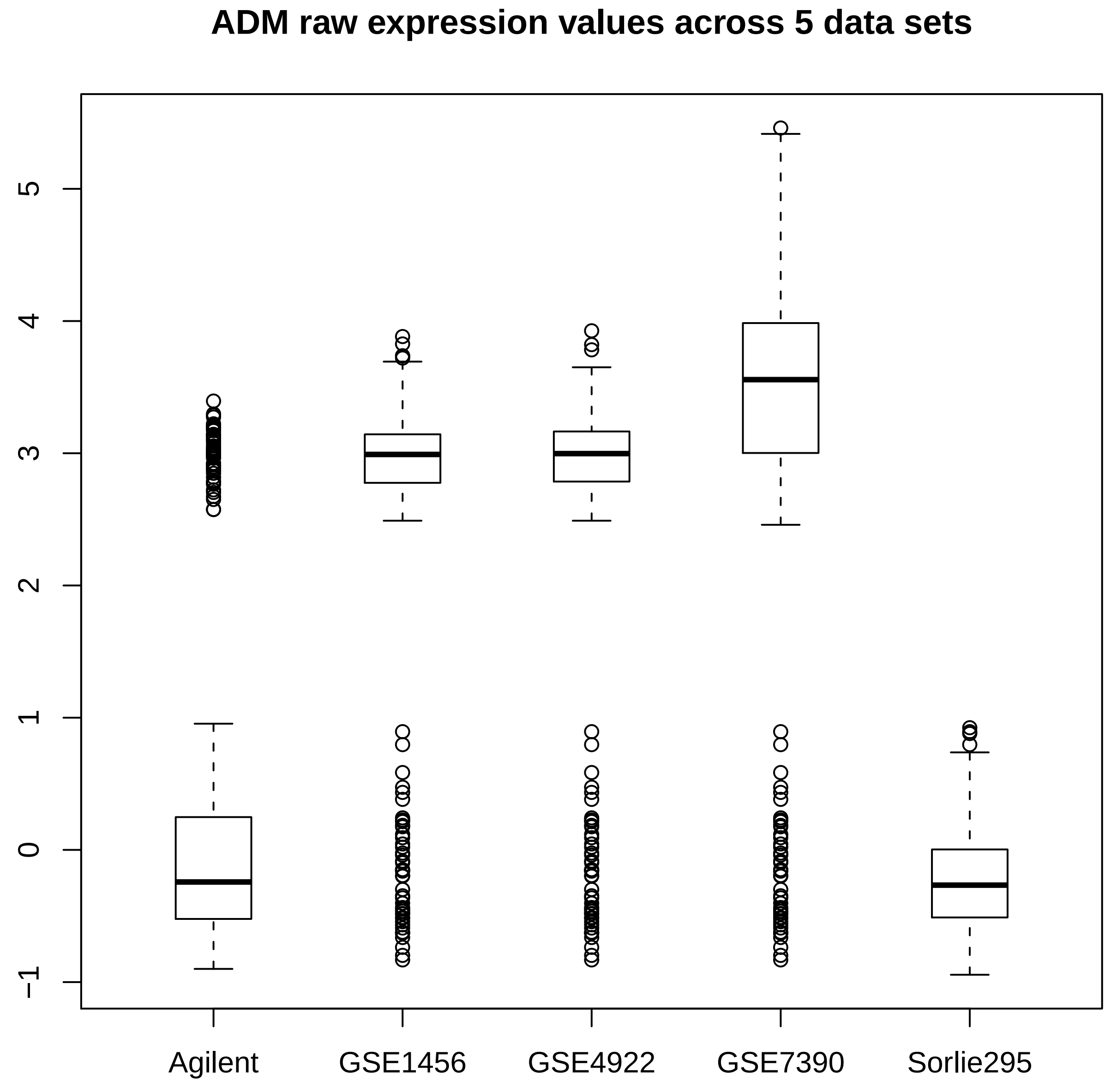

ADM raw expression values across 5 data sets
5
4
3
2
1
0
−1
Agilent
GSE1456
GSE4922
GSE7390
Sorlie295

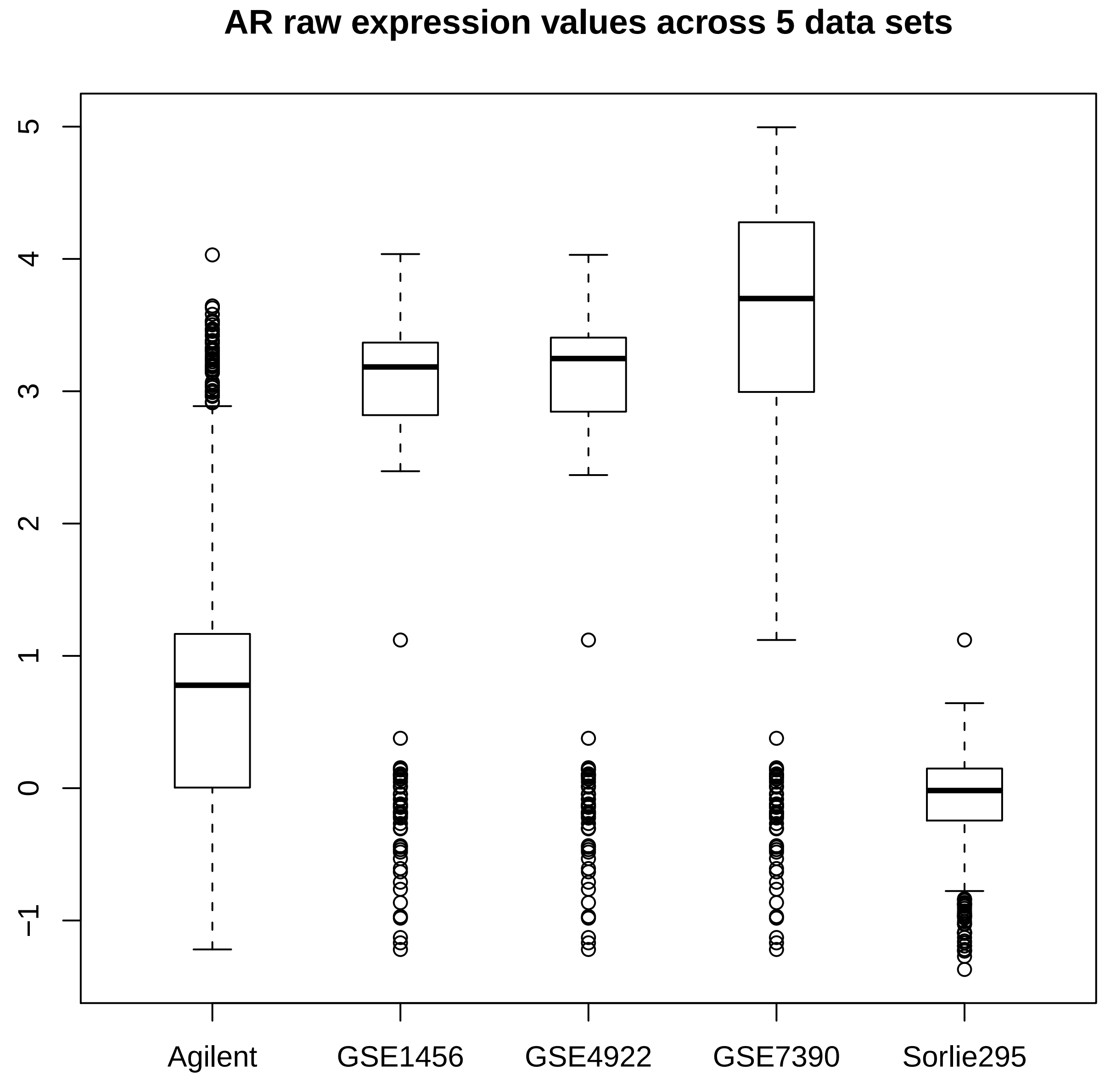
AR raw expression values across 5 data sets
5
4
3
2
1
0
−1
Agilent
GSE1456
GSE4922
GSE7390
Sorlie295

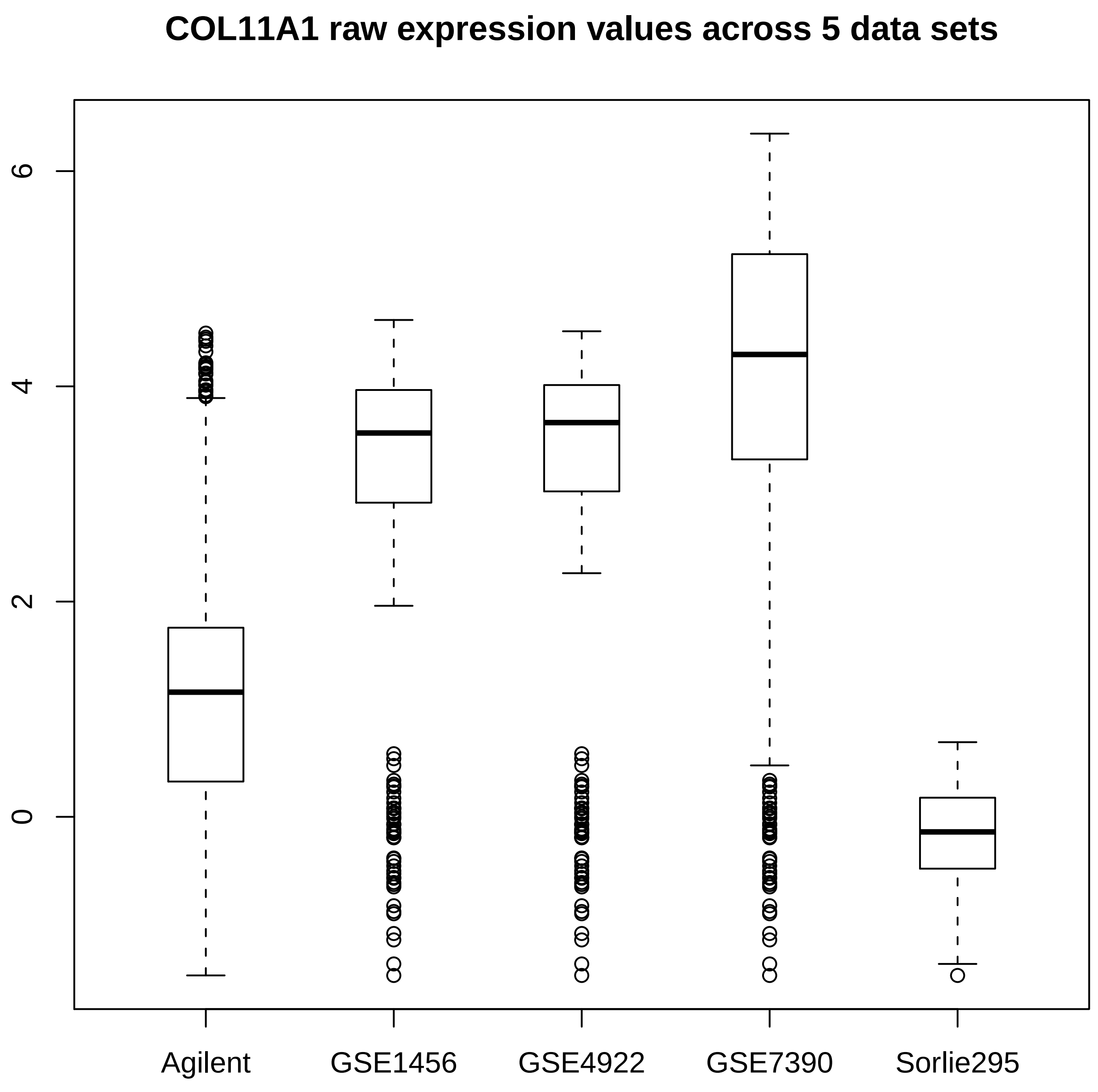

COL11A1 raw expression values across 5 data sets
0
2
4
6
Agilent
GSE1456
GSE4922
GSE7390
Sorlie295

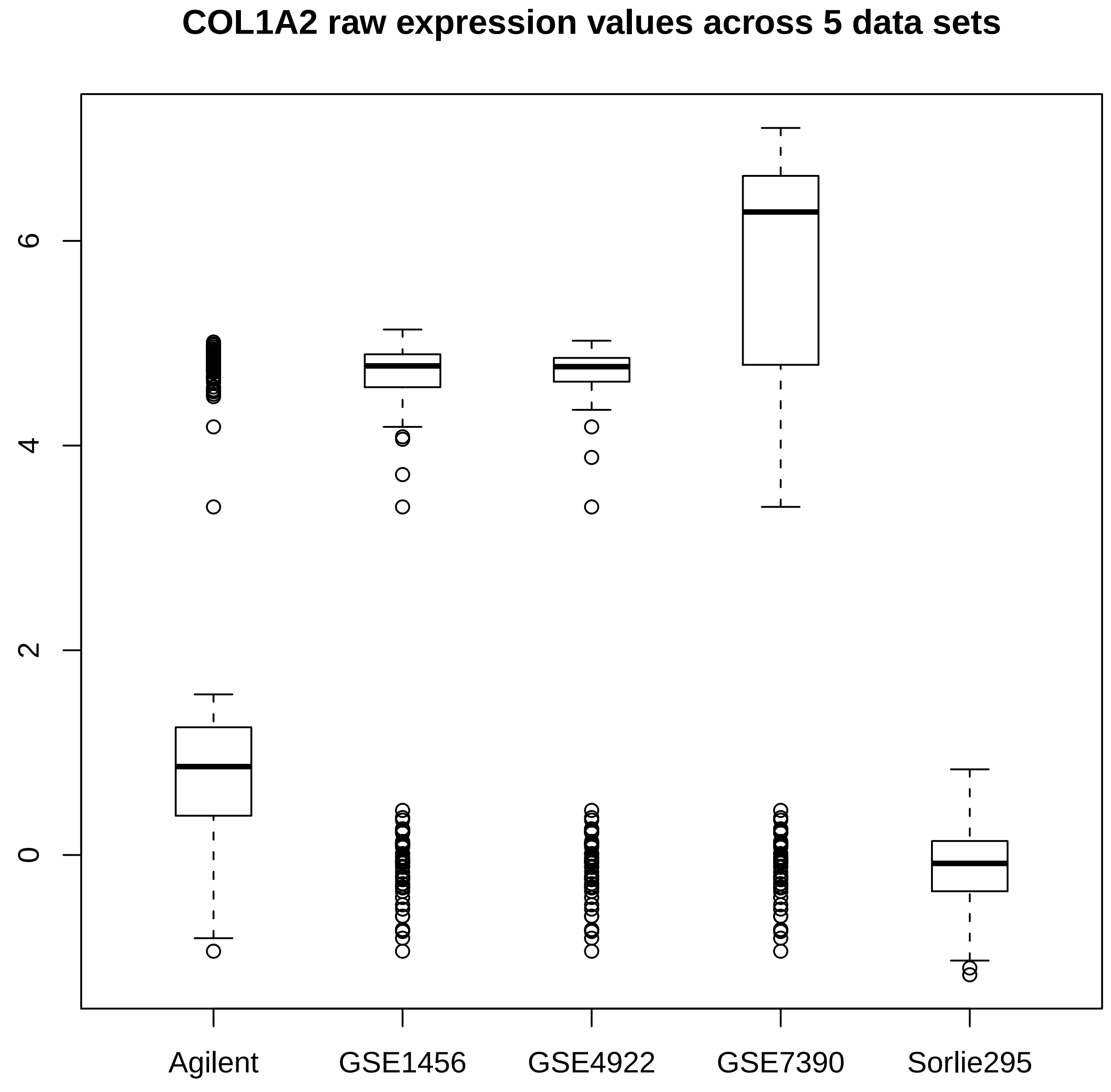
COL1A2 raw expression values across 5 data sets
0
2
4
6
Agilent
GSE1456
GSE4922
GSE7390
Sorlie295

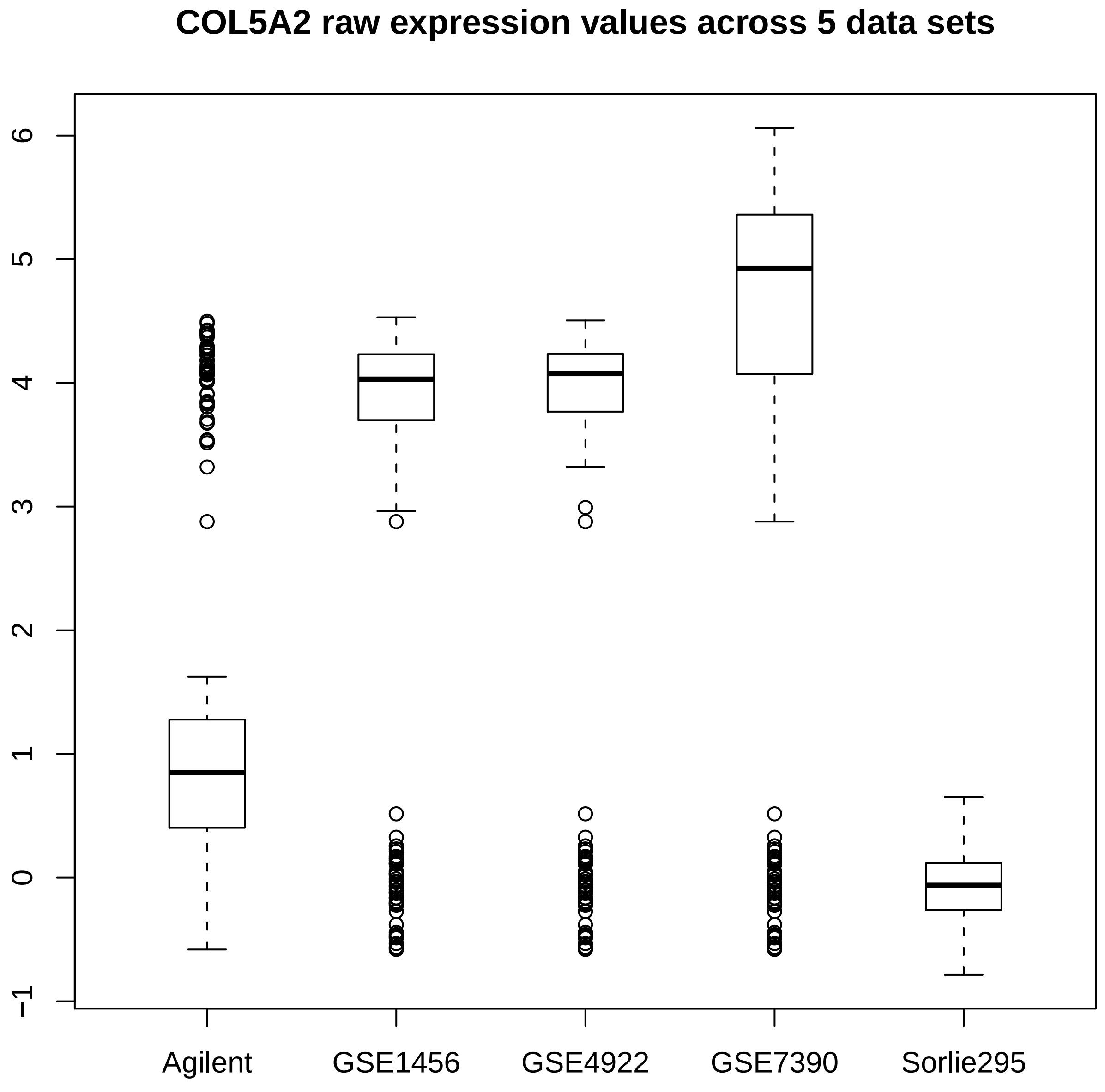

COL5A2 raw expression values across 5 data sets
6
5
4
3
2
1
0
−1
Agilent
GSE1456
GSE4922
GSE7390
Sorlie295

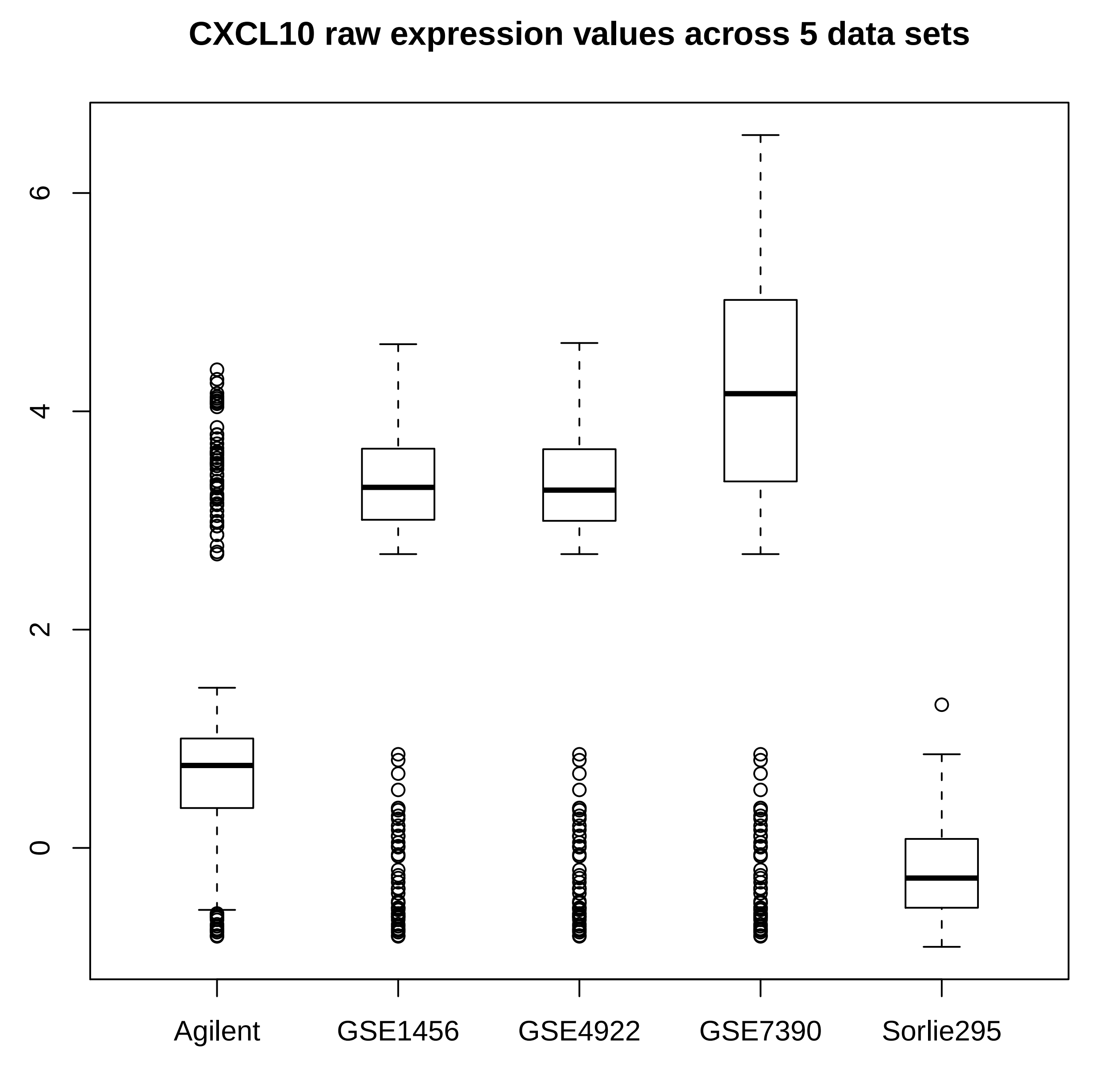

CXCL10 raw expression values across 5 data sets
0
2
4
6
Agilent
GSE1456
GSE4922
GSE7390
Sorlie295

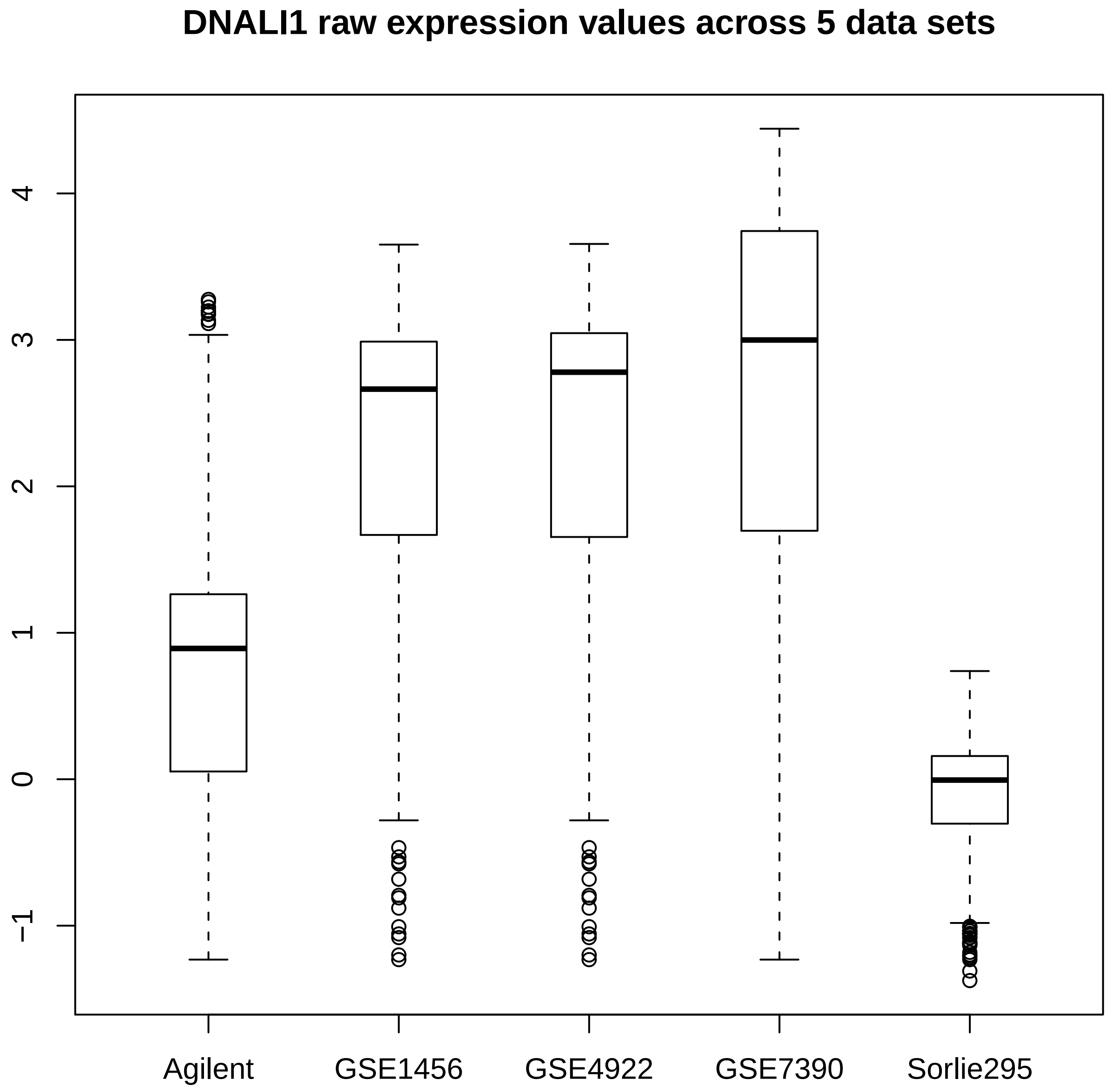
DNALI1 raw expression values across 5 data sets
4
3
2
1
0
−1
Agilent
GSE1456
GSE4922
GSE7390
Sorlie295

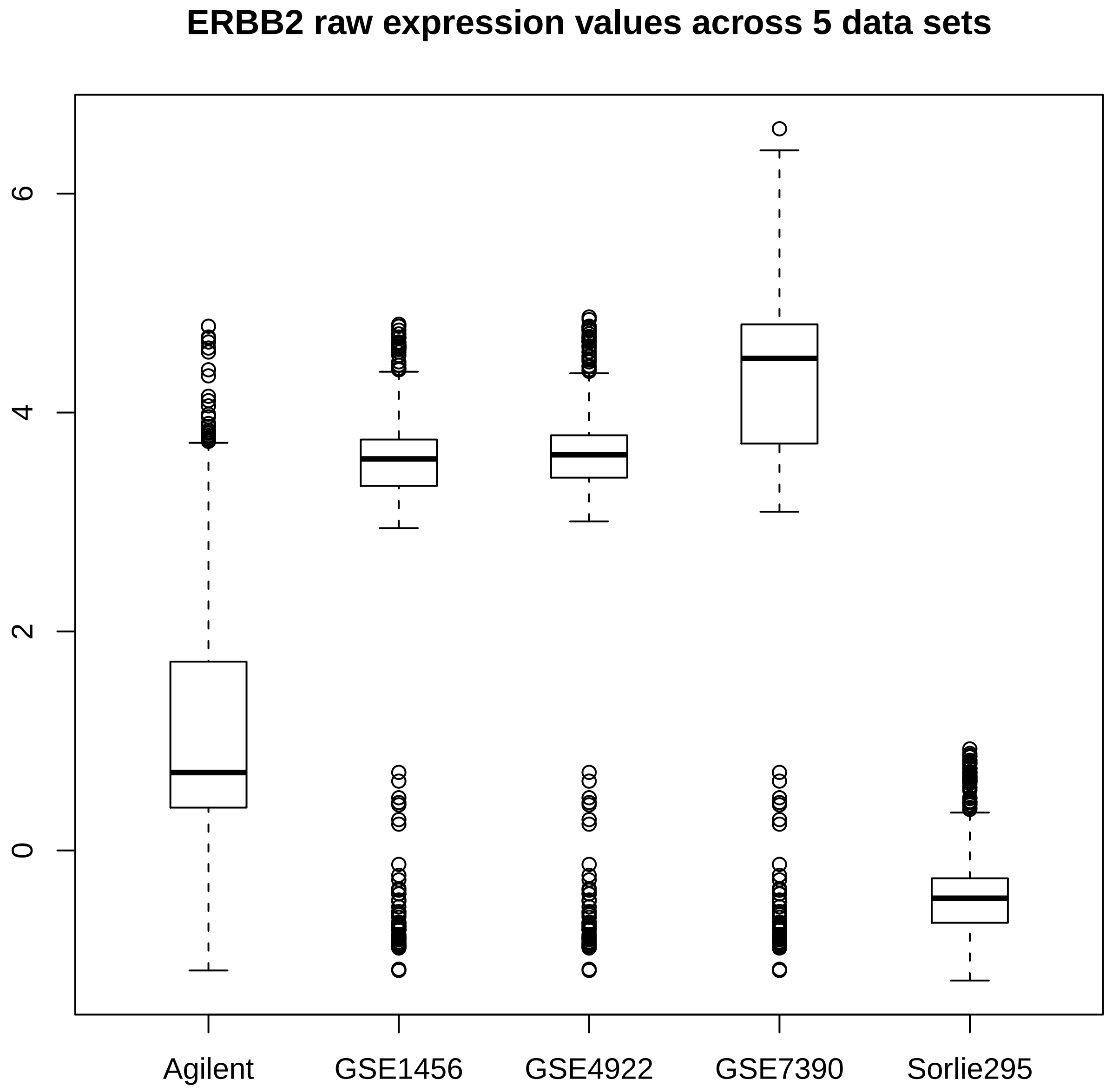

ERBB2 raw expression values across 5 data sets
0
2
4
6
Agilent
GSE1456
GSE4922
GSE7390
Sorlie295

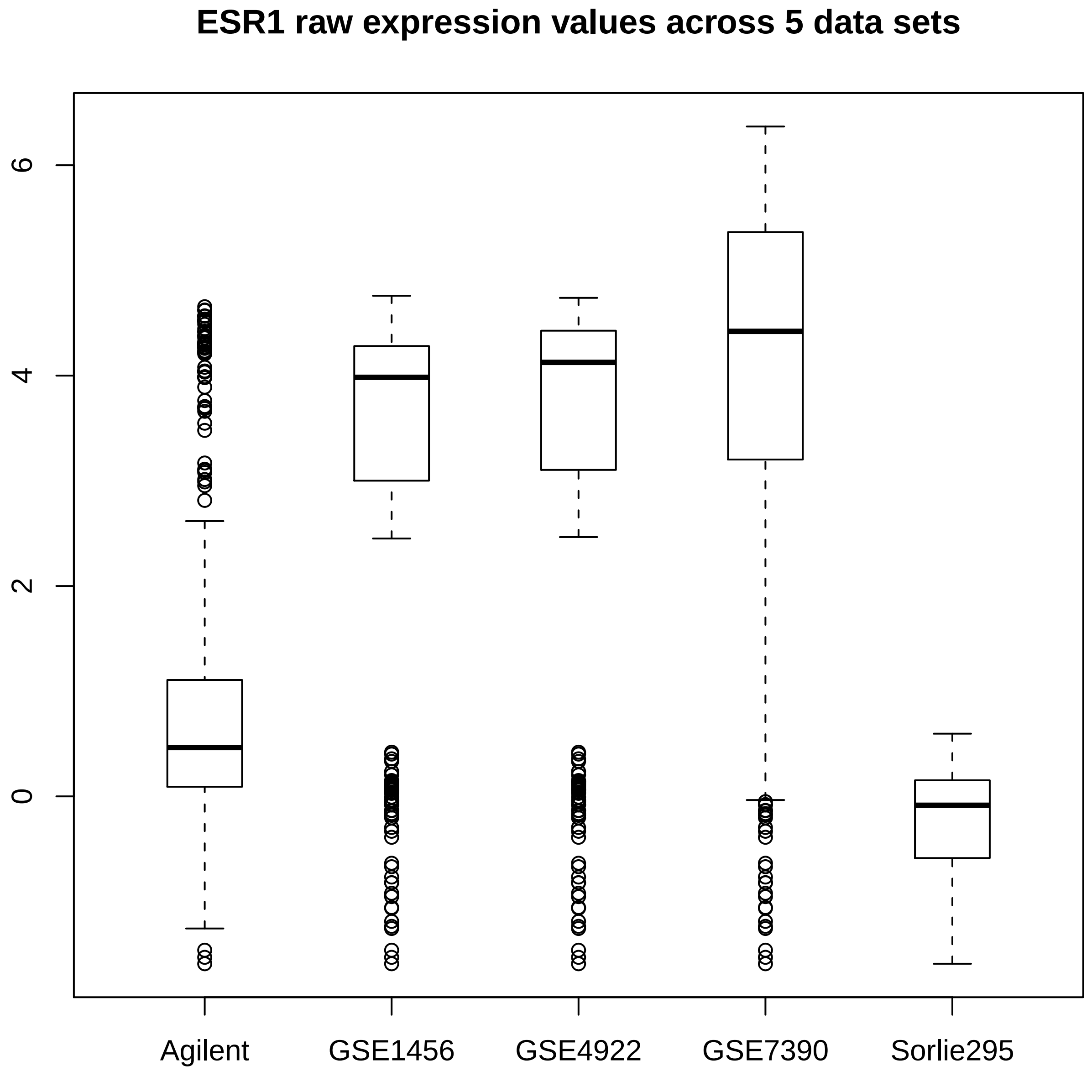
ESR1 raw expression values across 5 data sets
6
4
2
0
Agilent
GSE1456
GSE4922
GSE7390
Sorlie295

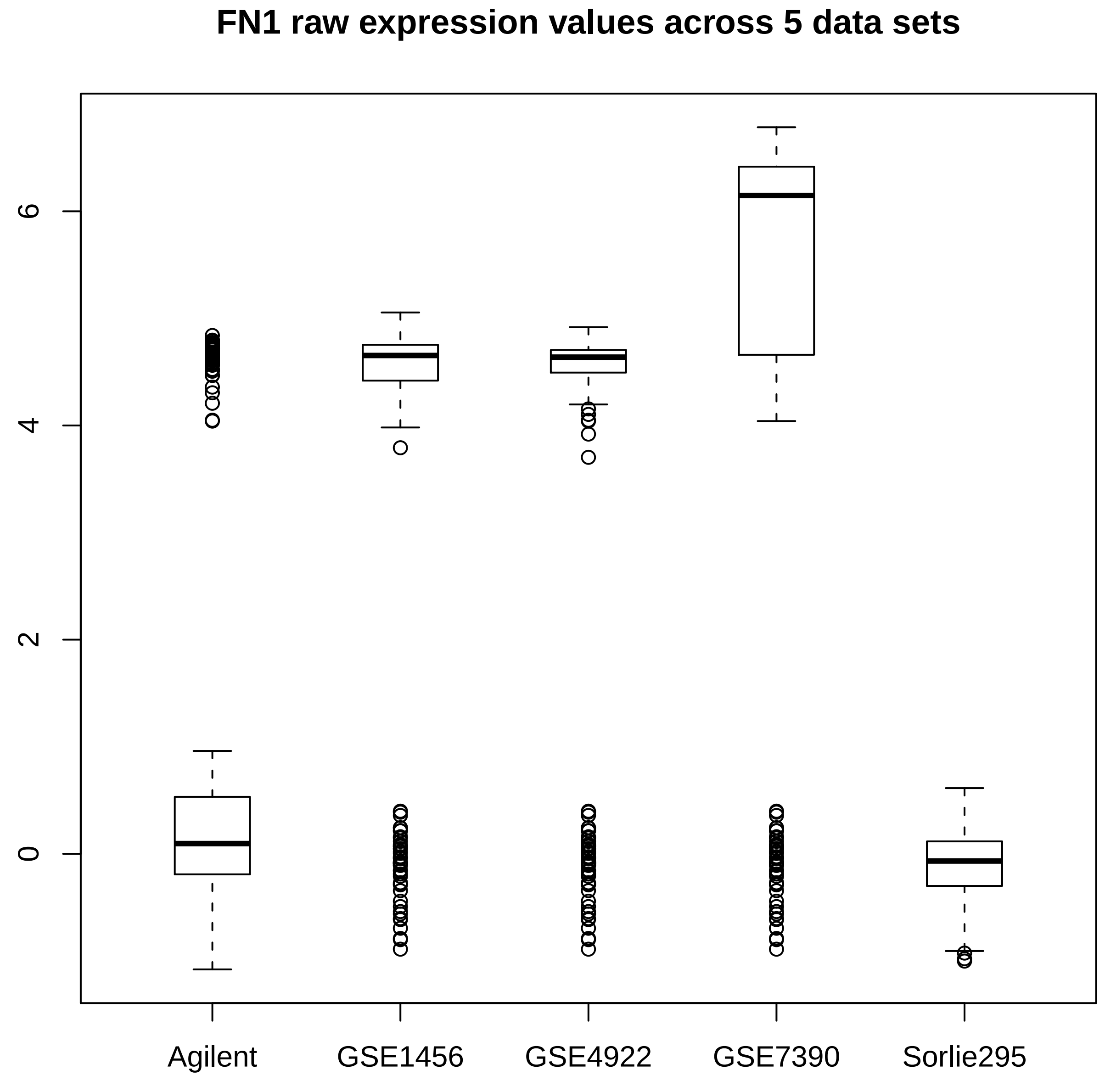
FN1 raw expression values across 5 data sets
0
2
4
6
Agilent
GSE1456
GSE4922
GSE7390
Sorlie295

FOXA1 raw expression values across 5 data sets

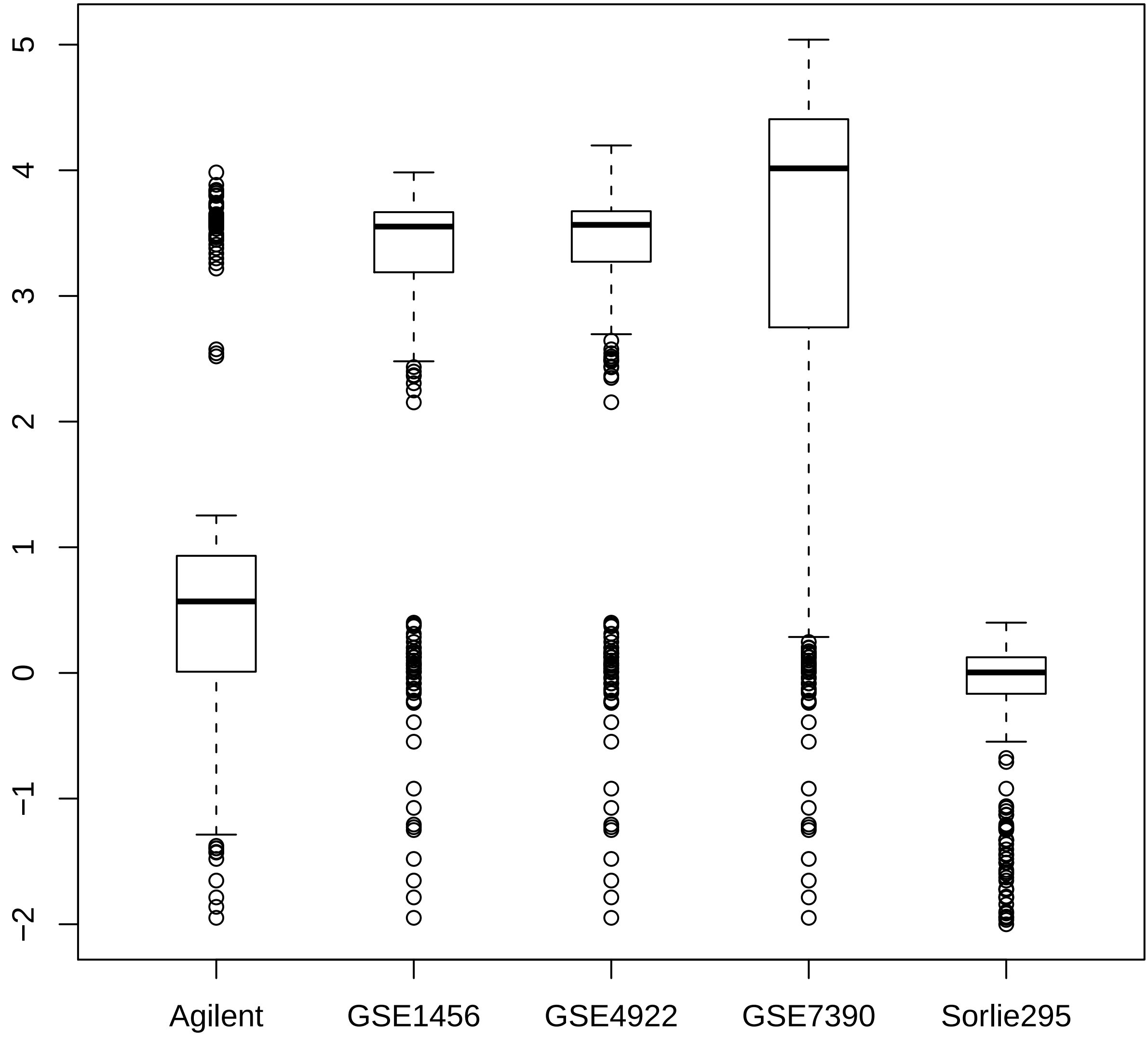

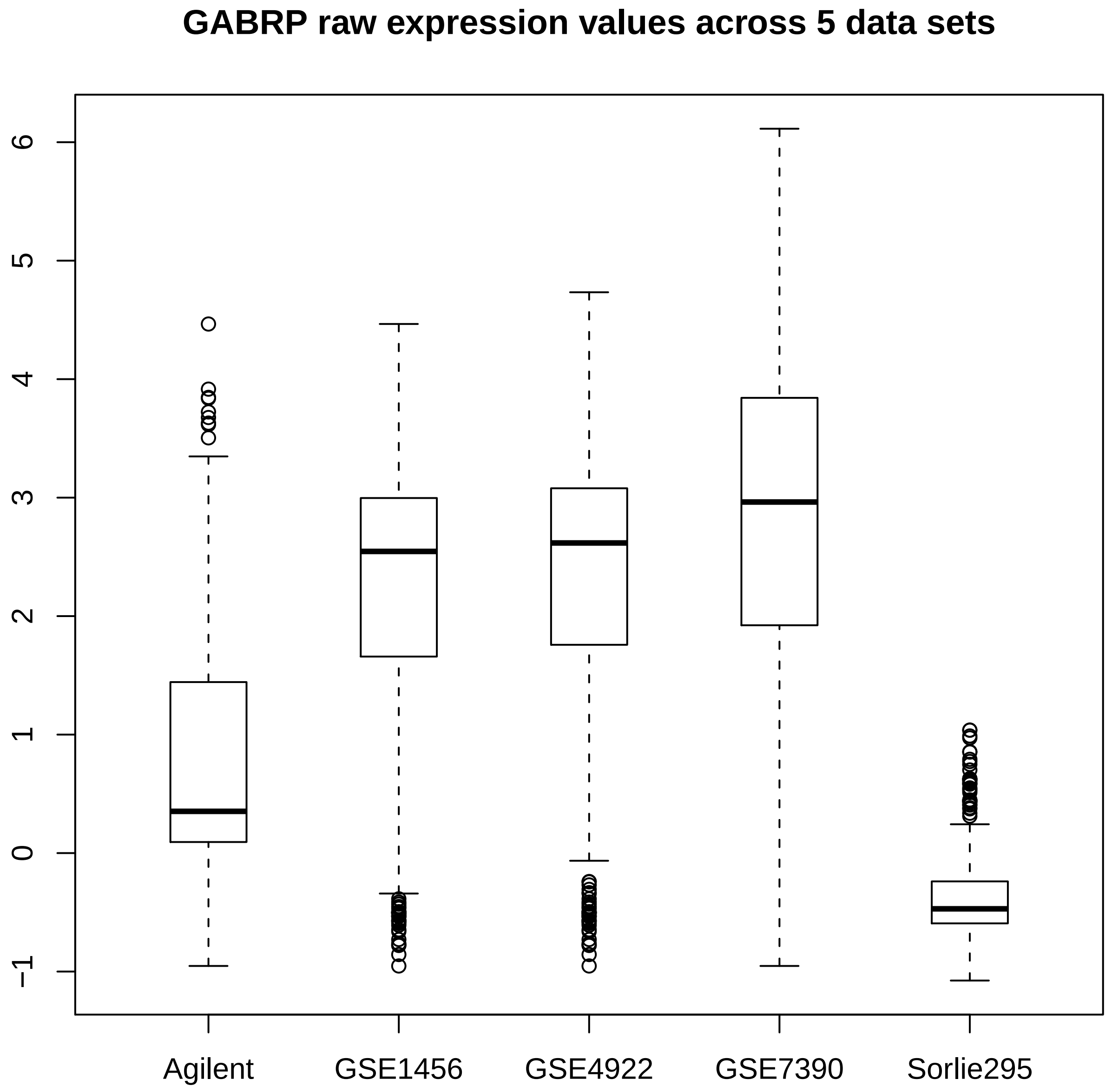
GABRP raw expression values across 5 data sets
−1
0
1
2
3
4
5
6
Agilent
GSE1456
GSE4922
GSE7390
Sorlie295

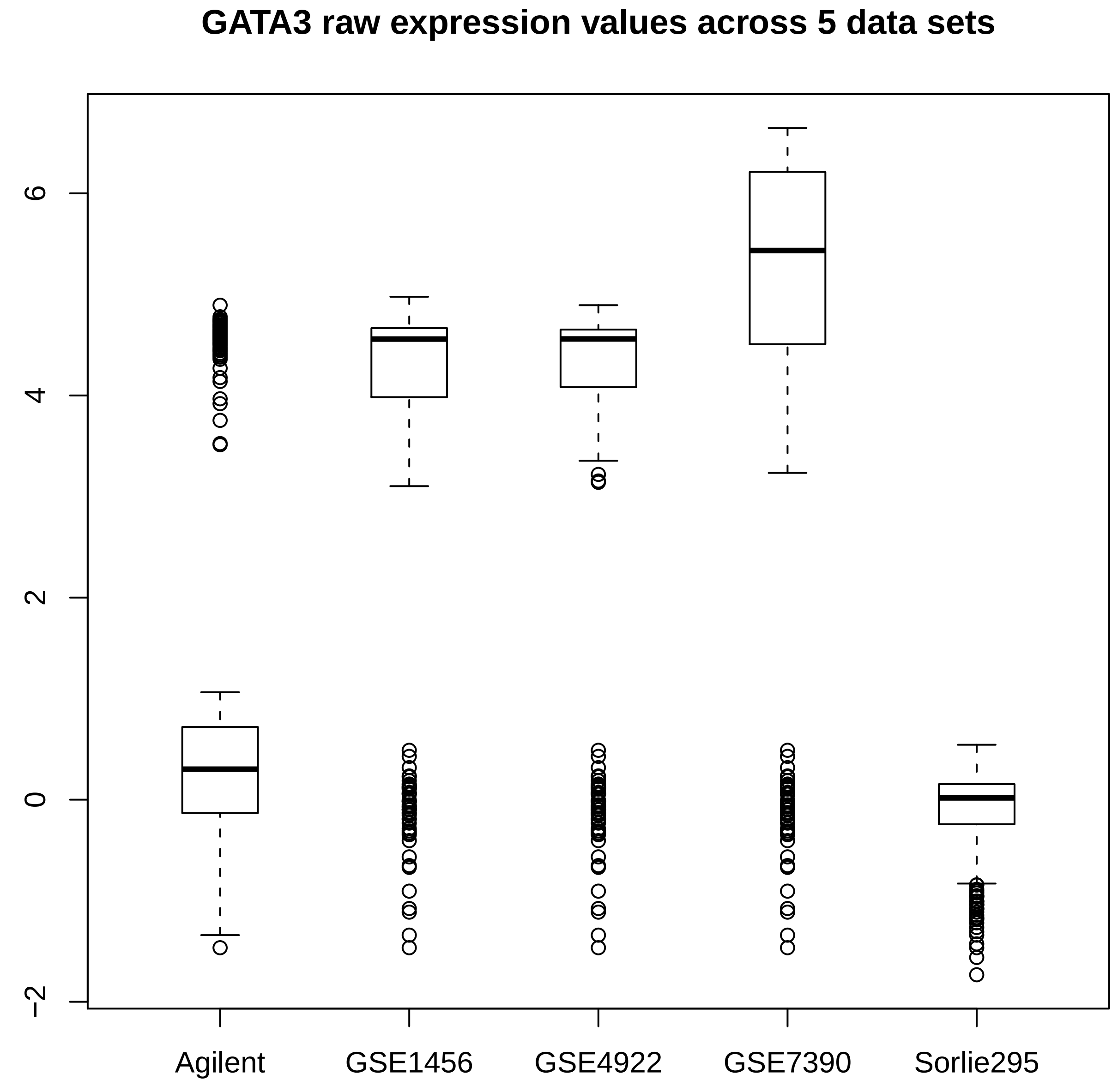
GATA3 raw expression values across 5 data sets
6
4
2
0
−2
Agilent
GSE1456
GSE4922
GSE7390
Sorlie295

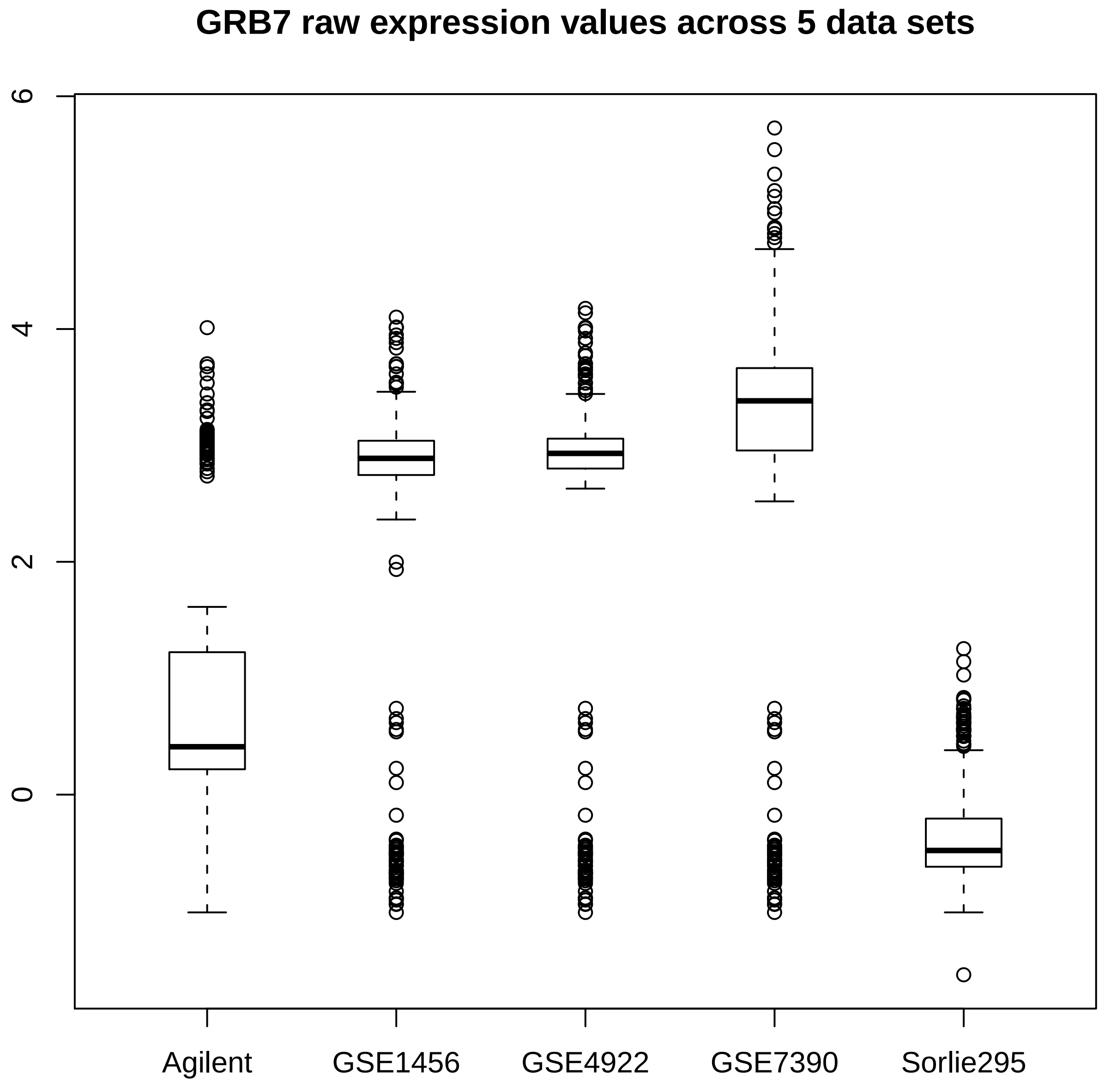

GRB7 raw expression values across 5 data sets
6
4
2
0
Agilent
GSE1456
GSE4922
GSE7390
Sorlie295

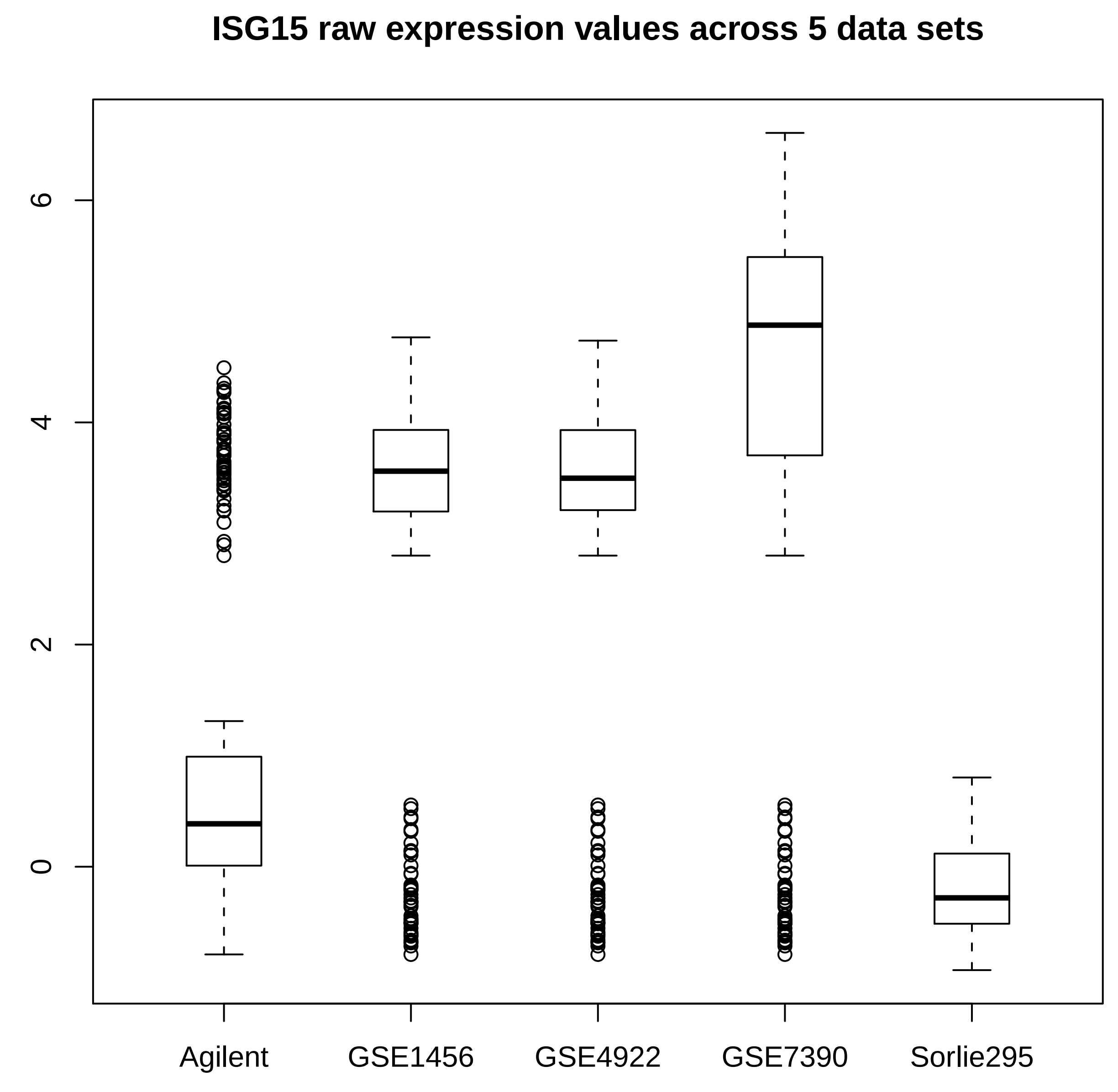
ISG15 raw expression values across 5 data sets
0
2
4
6
Agilent
GSE1456
GSE4922
GSE7390
Sorlie295

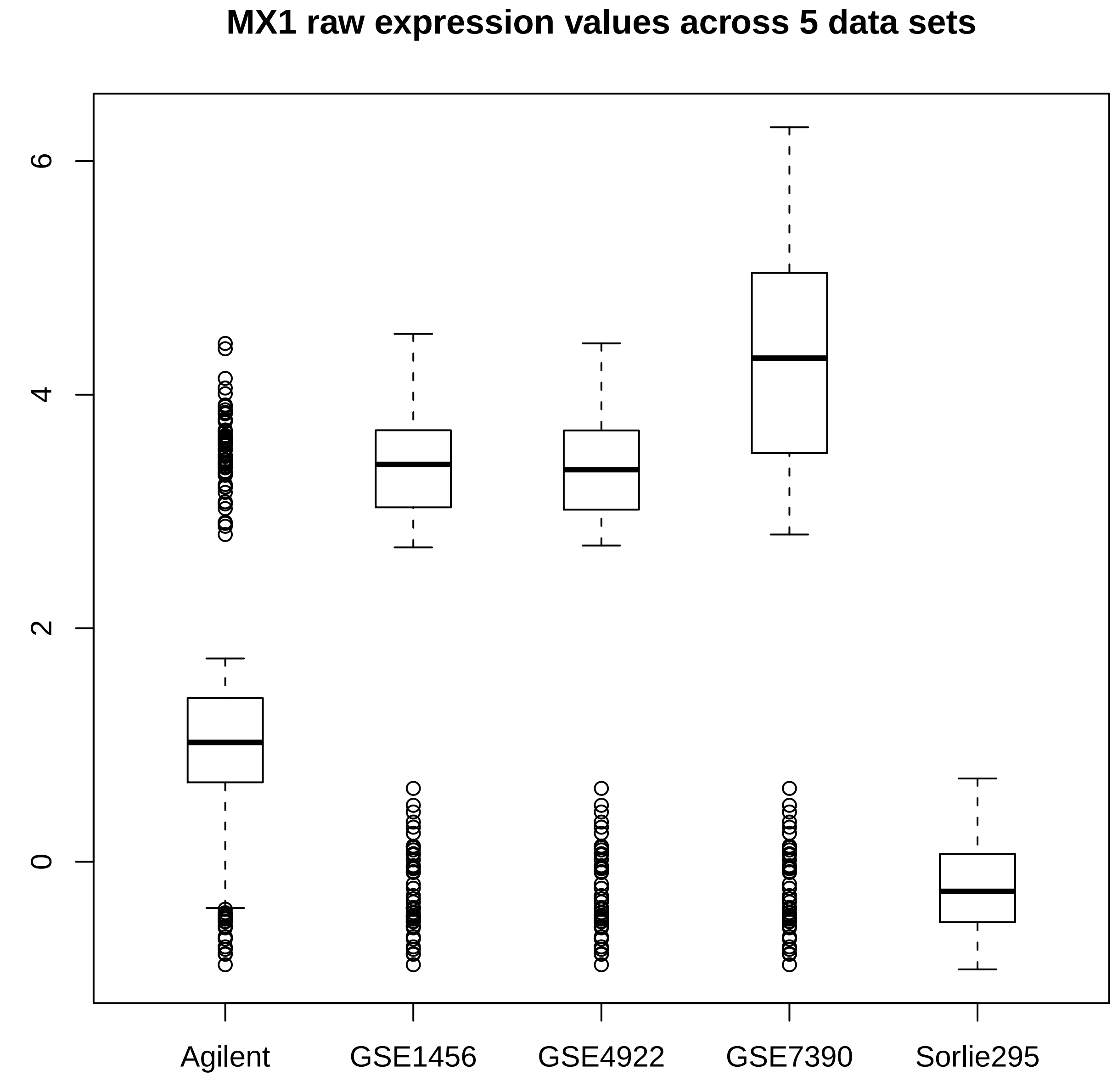
MX1 raw expression values across 5 data sets
0
2
4
6
Agilent
GSE1456
GSE4922
GSE7390
Sorlie295

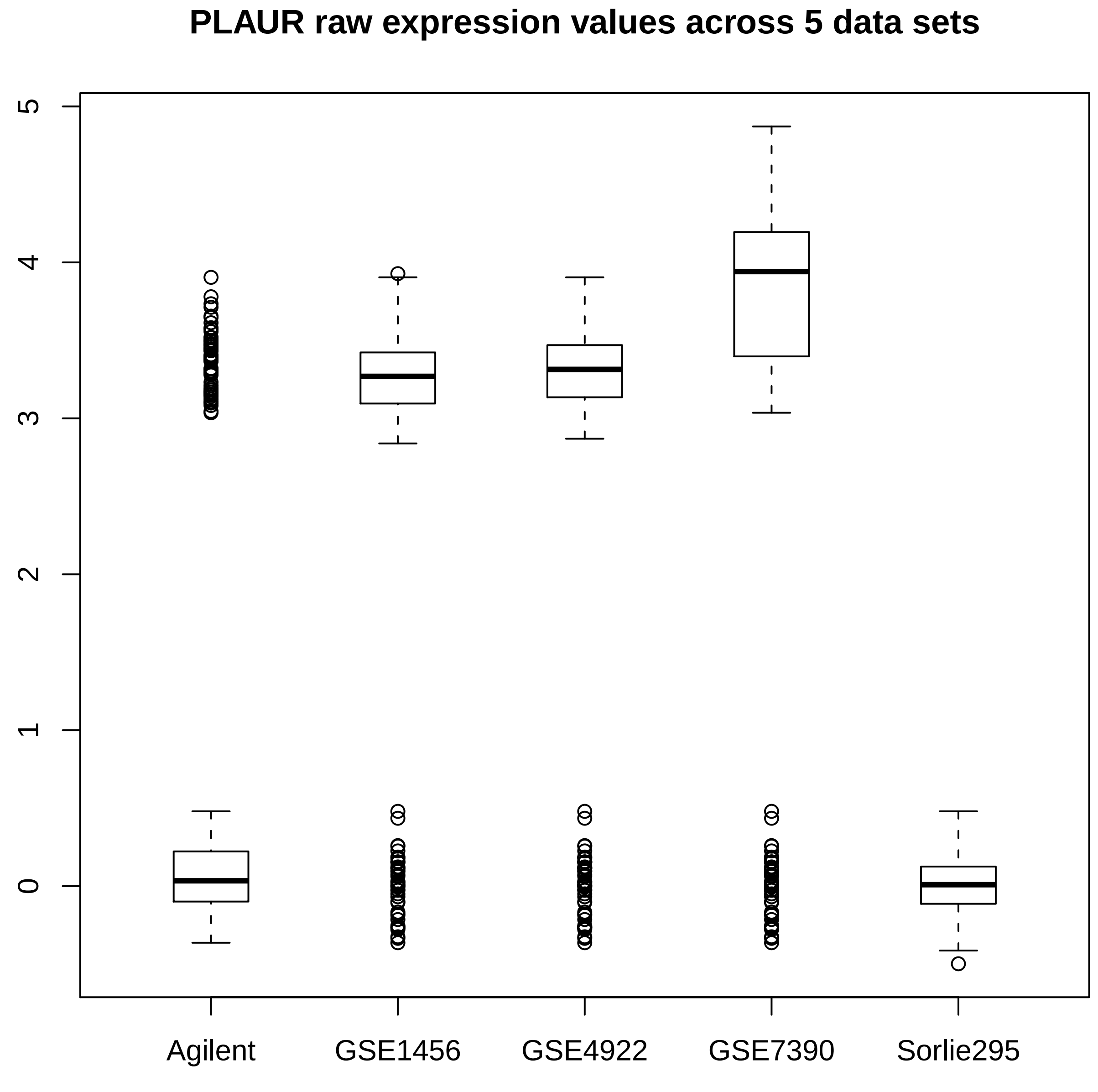

PLAUR raw expression values across 5 data sets
0
1
2
3
4
5
Agilent
GSE1456
GSE4922
GSE7390
Sorlie295

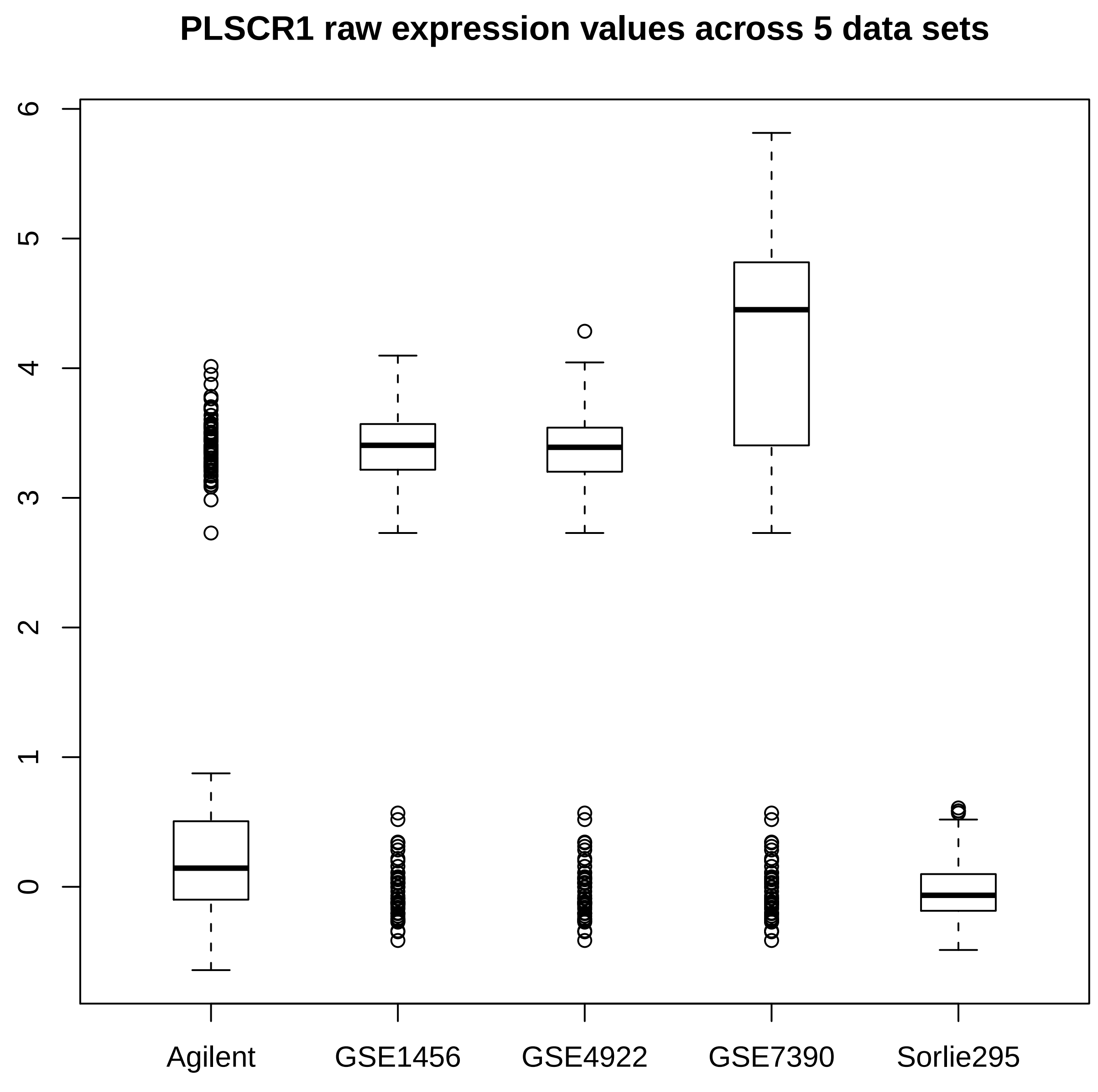
PLSCR1 raw expression values across 5 data sets
0
1
2
3
4
5
6
Agilent
GSE1456
GSE4922
GSE7390
Sorlie295

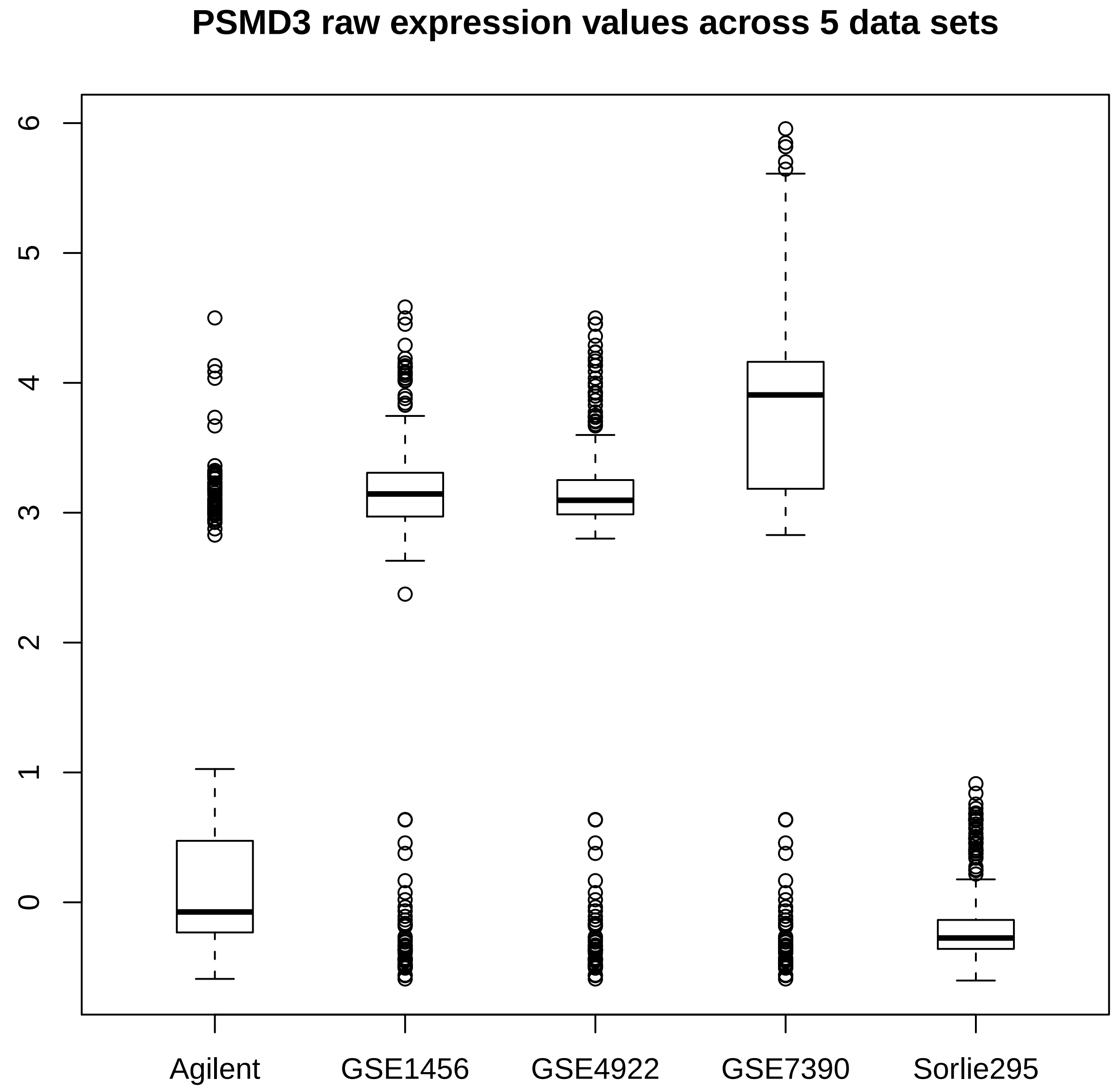

PSMD3 raw expression values across 5 data sets
0
1
2
3
4
5
6
Agilent
GSE1456
GSE4922
GSE7390
Sorlie295

STAT1 raw expression values across 5 data sets

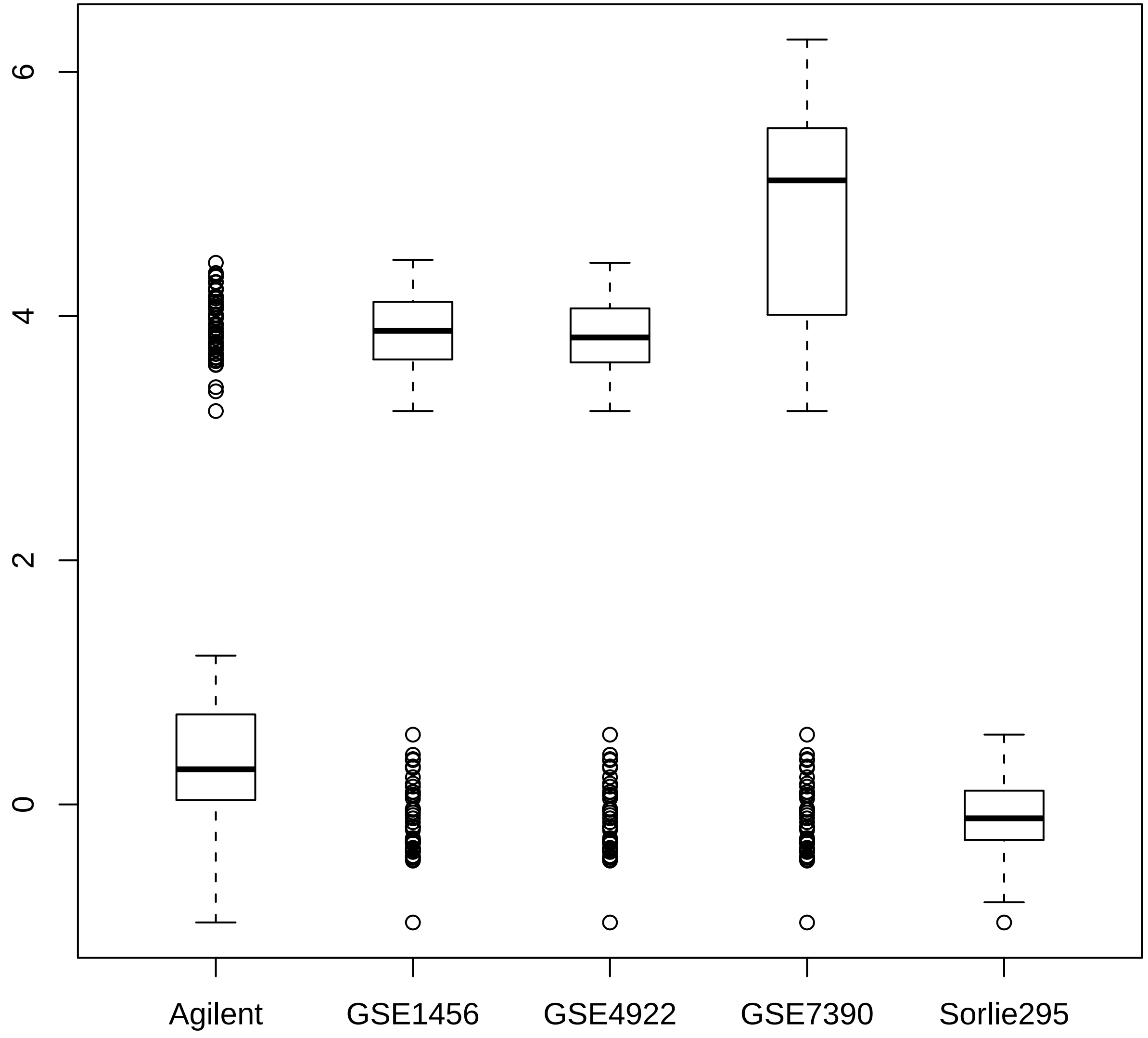

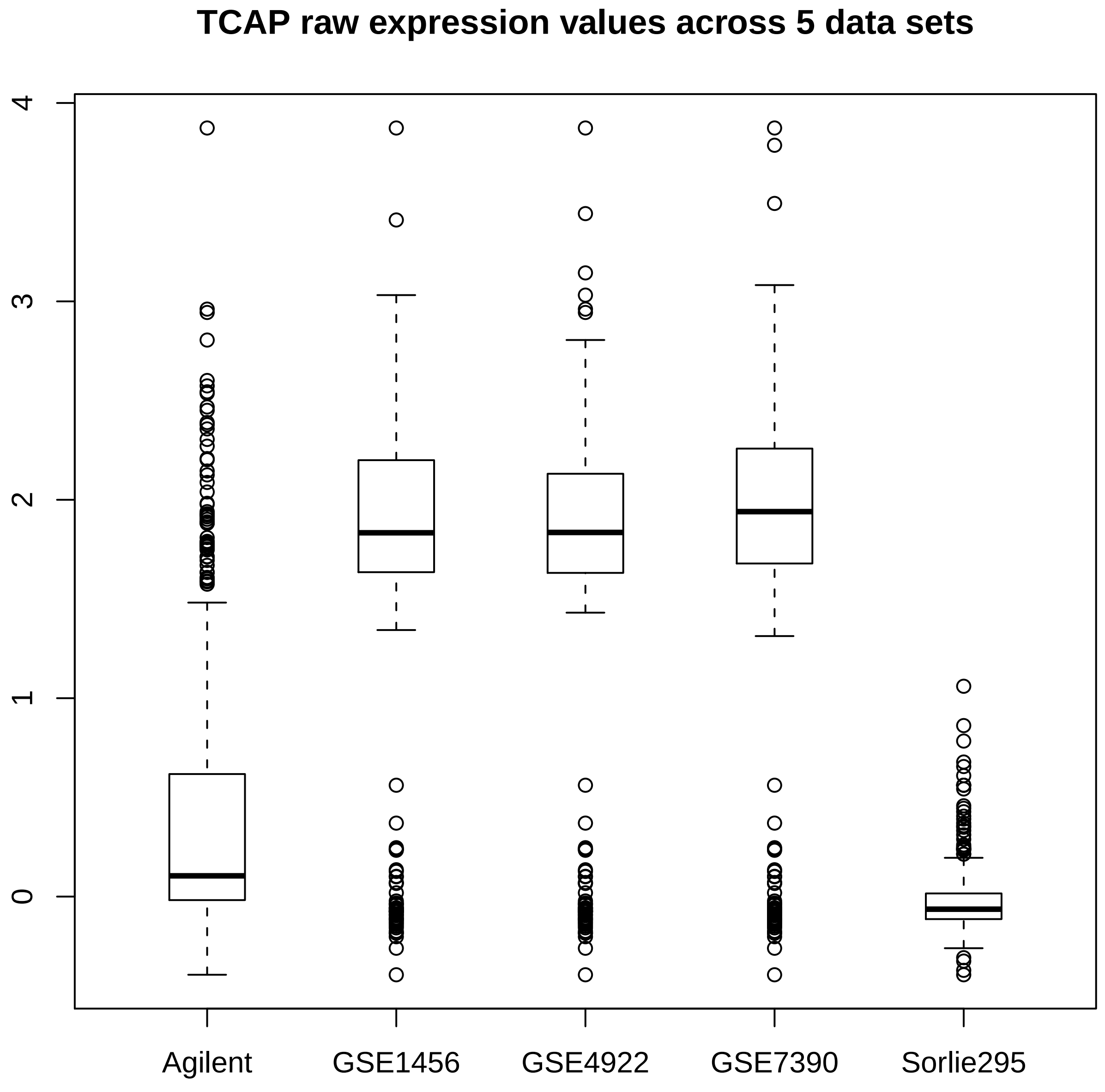
TCAP raw expression values across 5 data sets
0
1
2
3
4
Agilent
GSE1456
GSE4922
GSE7390
Sorlie295
