# Supplementary material for: Bimodal gene expression patterns in breast cancer
Source: BMC Genomics. 2010 Feb 10;11(Suppl 1):S8. doi: 10.1186/1471-2164-11-S1-S8 (PMC2822536; doi:10.1186/1471-2164-11-S1-S8)
Supplement: Additional file 5 — Normalized data box plots. Expression profiles for bimodal genes in 5 data sets after normalization. Box Plots [file 1471-2164-11-S1-S8-S5.pdf]

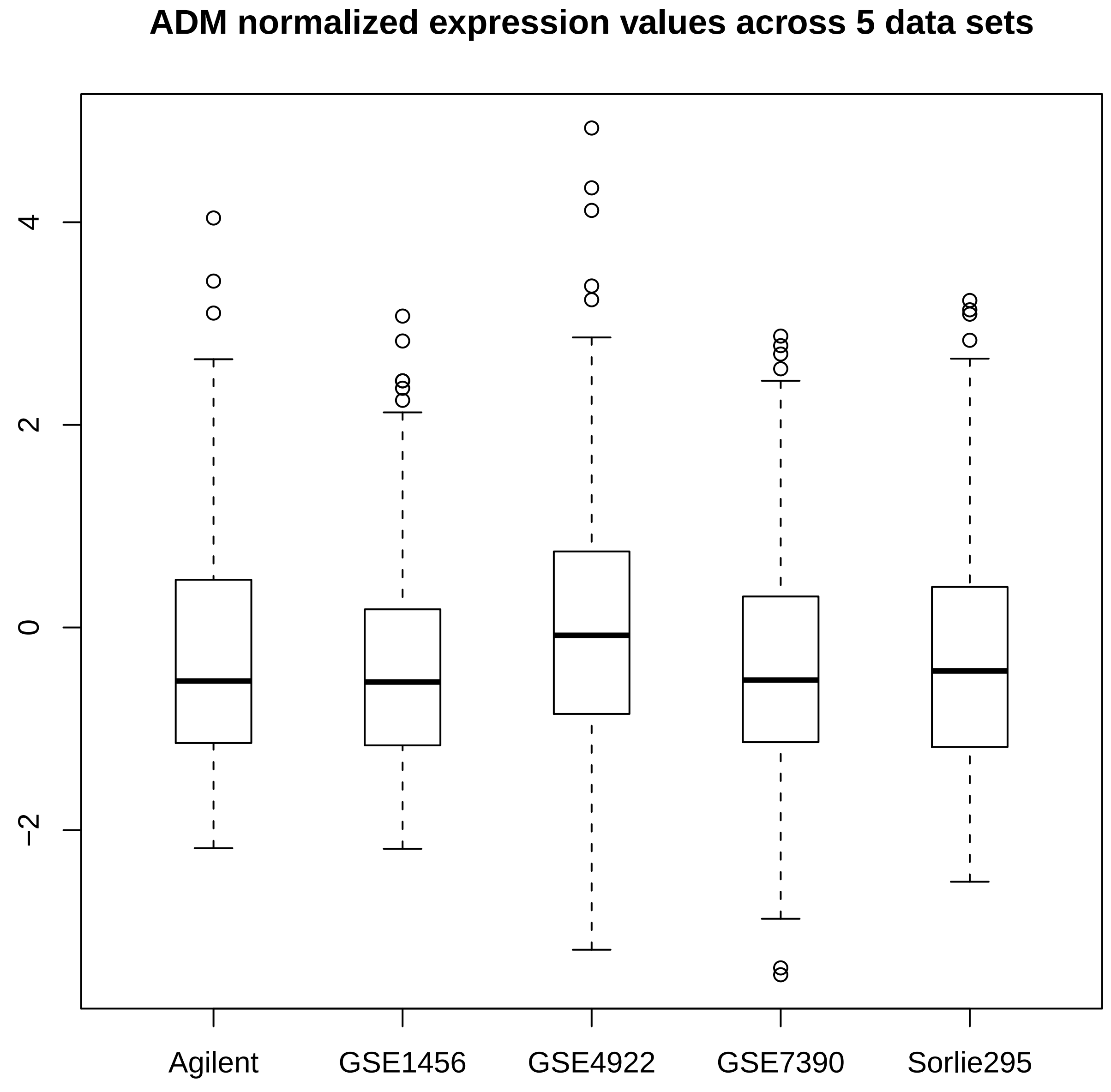
ADM normalized expression values across 5 data sets
4
2
0
−2
Agilent
GSE1456
GSE4922
GSE7390
Sorlie295

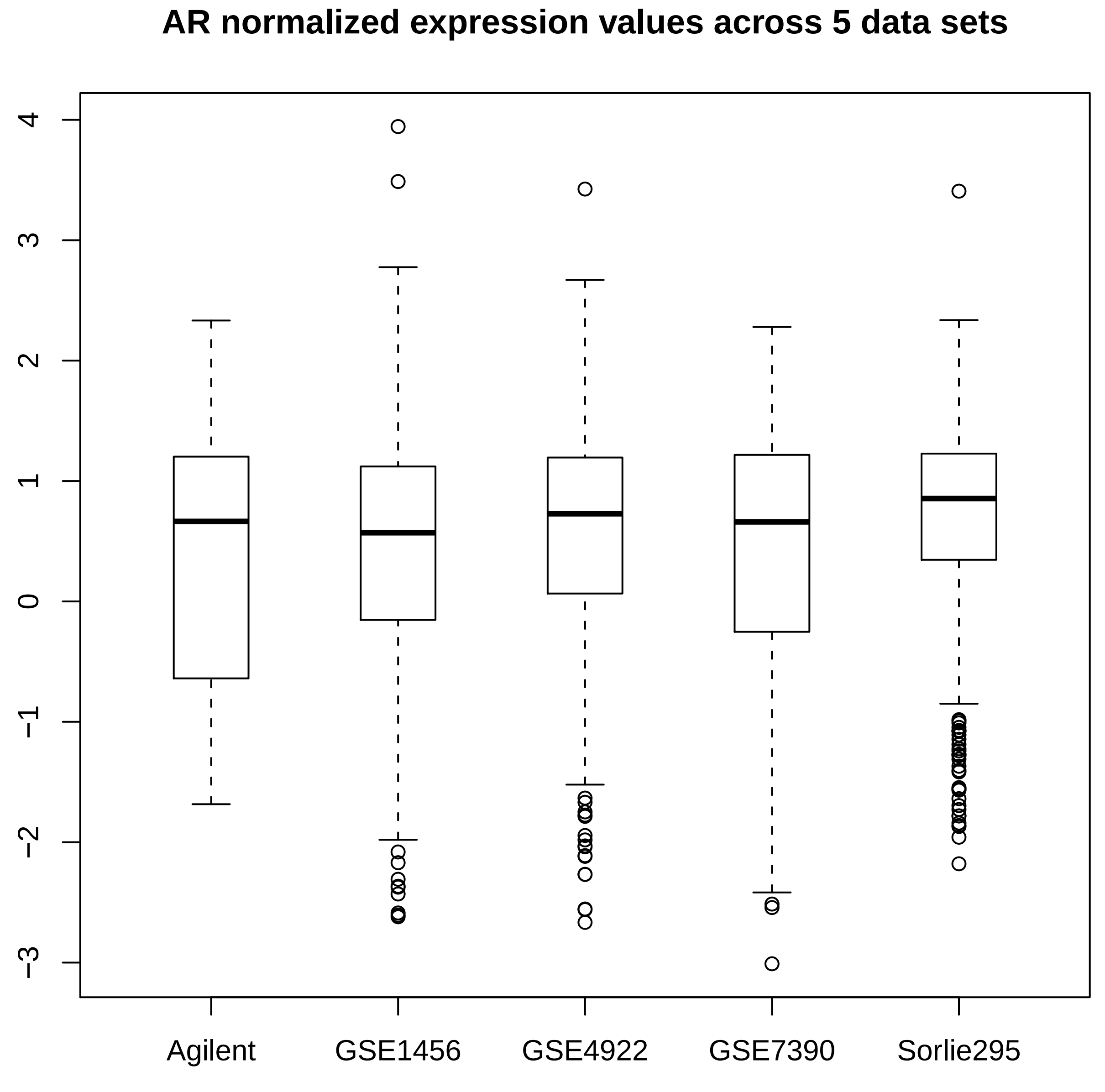
AR normalized expression values across 5 data sets
4
3
2
1
0
−1
−2
−3
Agilent
GSE1456
GSE4922
GSE7390
Sorlie295

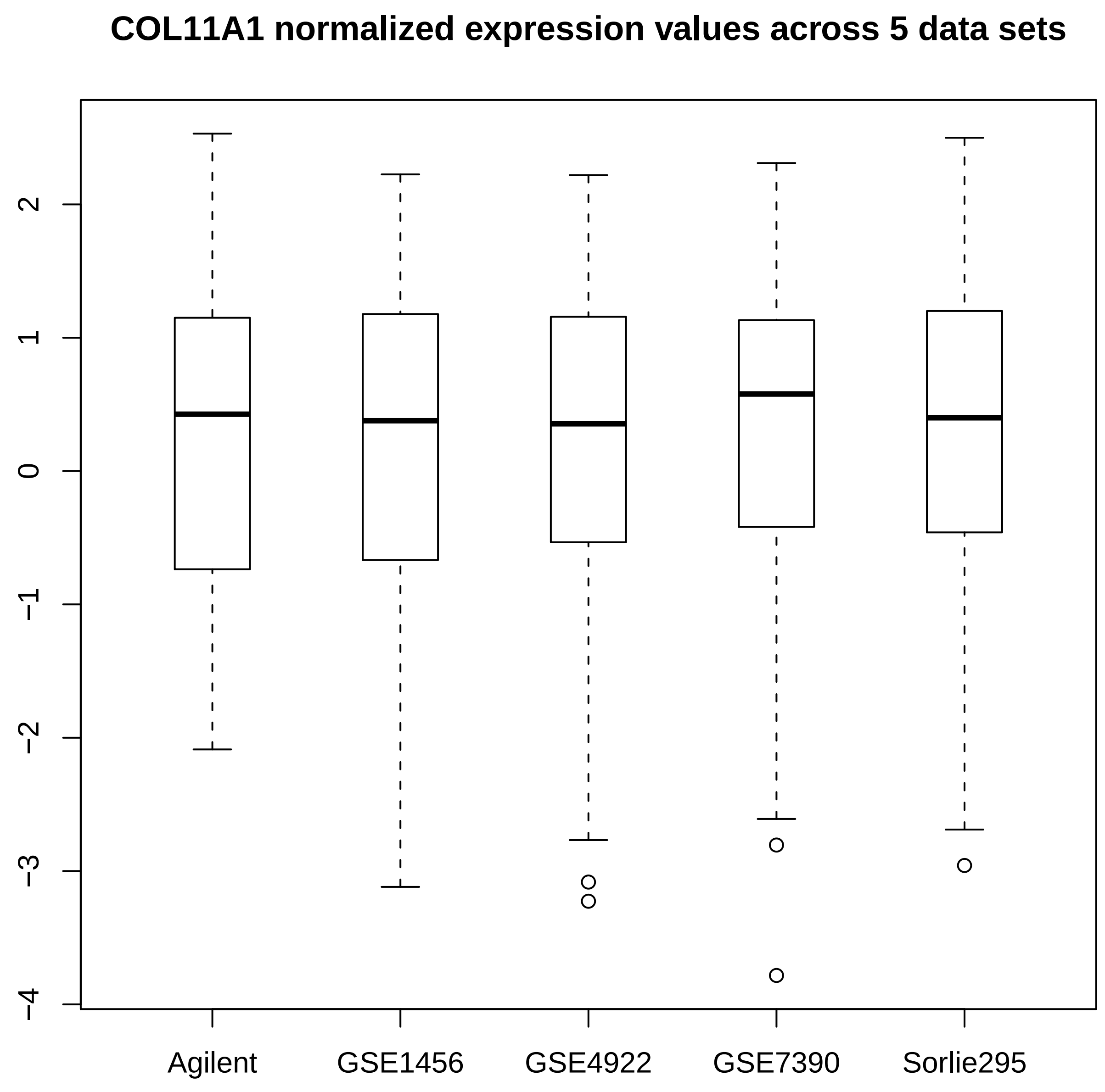

COL11A1 normalized expression values across 5 data sets
2
1
0
−1
−2
−3
−4
Agilent
GSE1456
GSE4922
GSE7390
Sorlie295

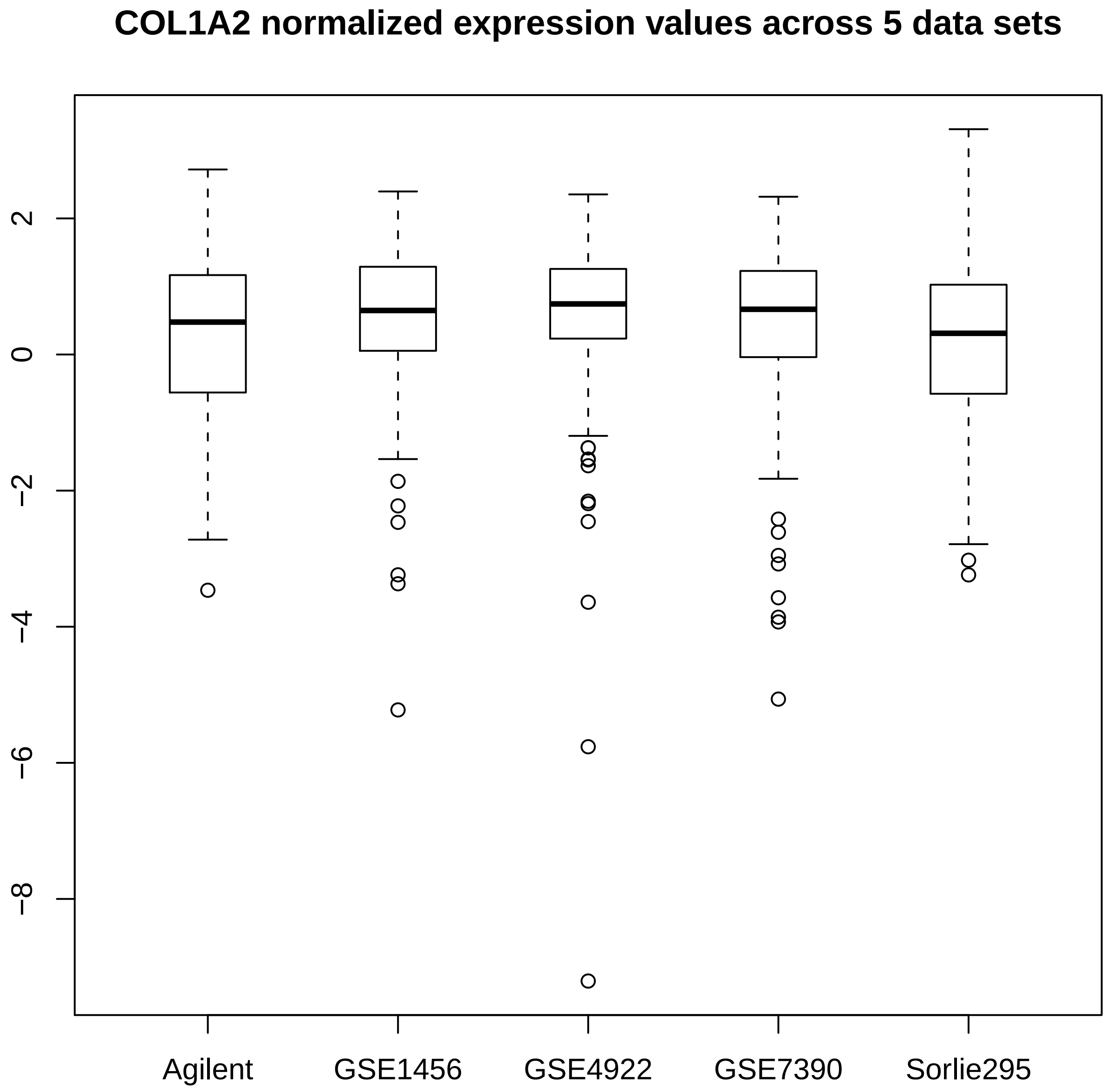
COL1A2 normalized expression values across 5 data sets
2
0
−2
−4
−6
−8
Agilent
GSE1456
GSE4922
GSE7390
Sorlie295

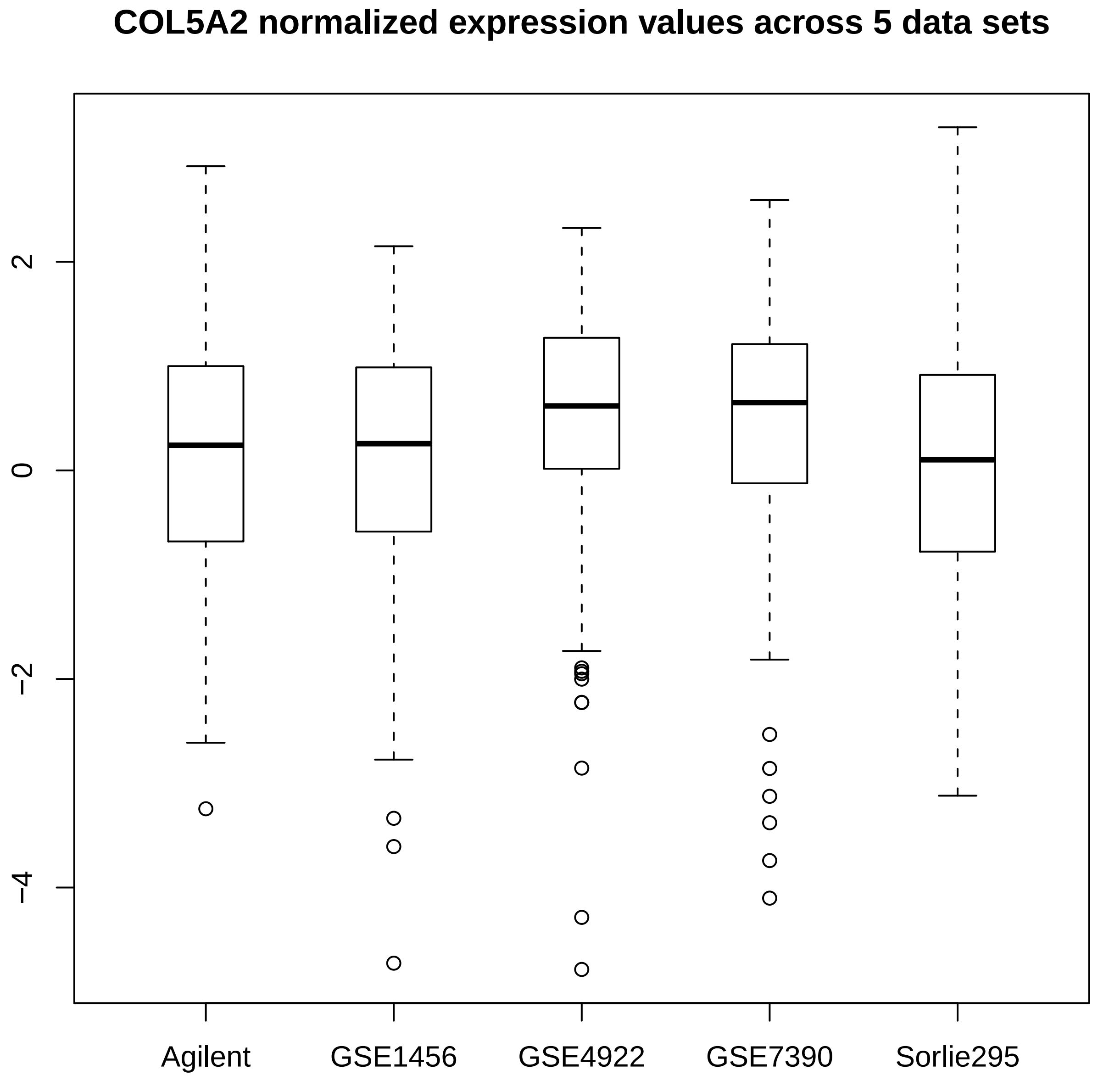
COL5A2 normalized expression values across 5 data sets
2
0
−2
−4
Agilent
GSE1456
GSE4922
GSE7390
Sorlie295

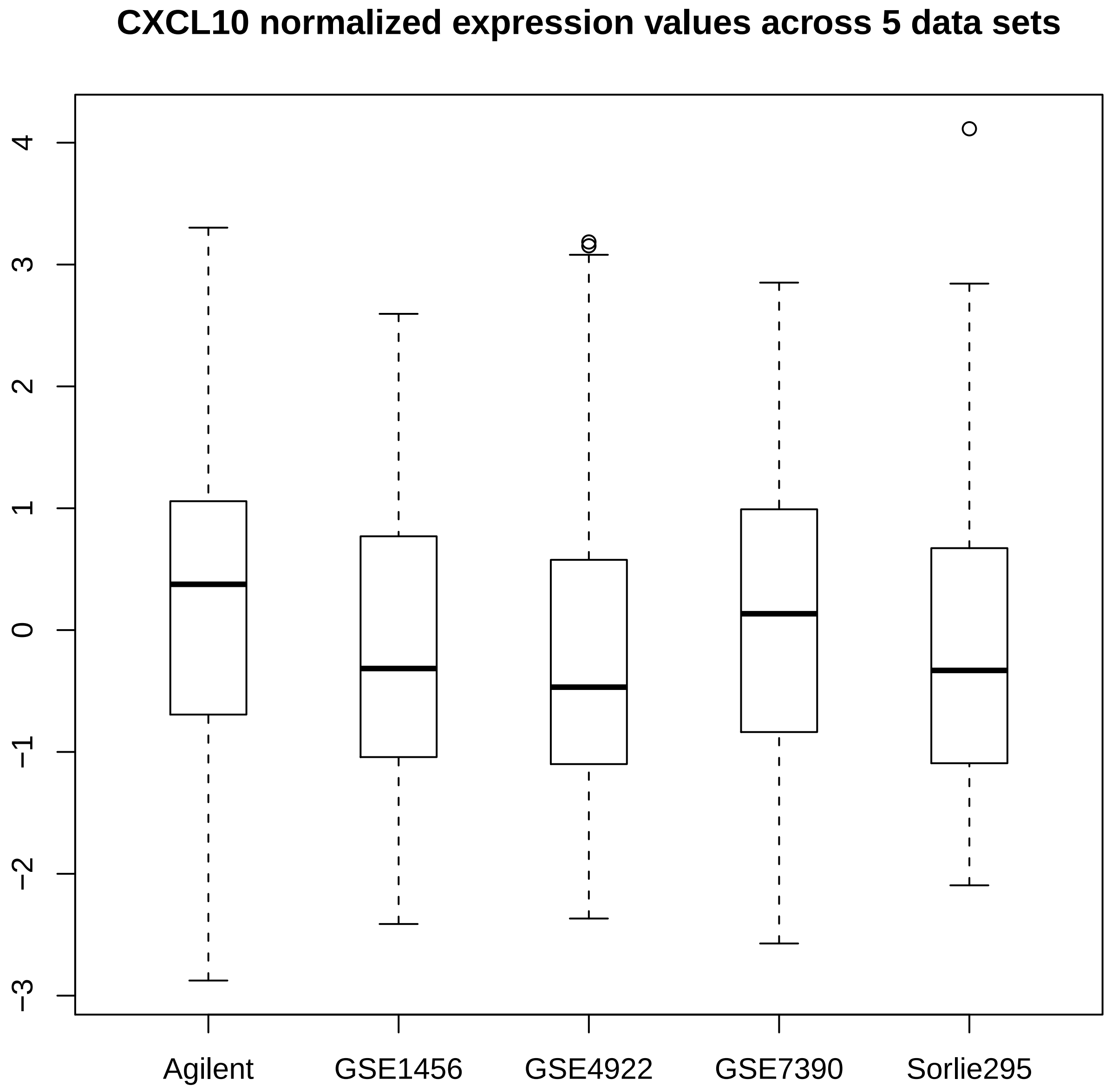
CXCL10 normalized expression values across 5 data sets
4
3
2
1
0
−1
−2
−3
Agilent
GSE1456
GSE4922
GSE7390
Sorlie295

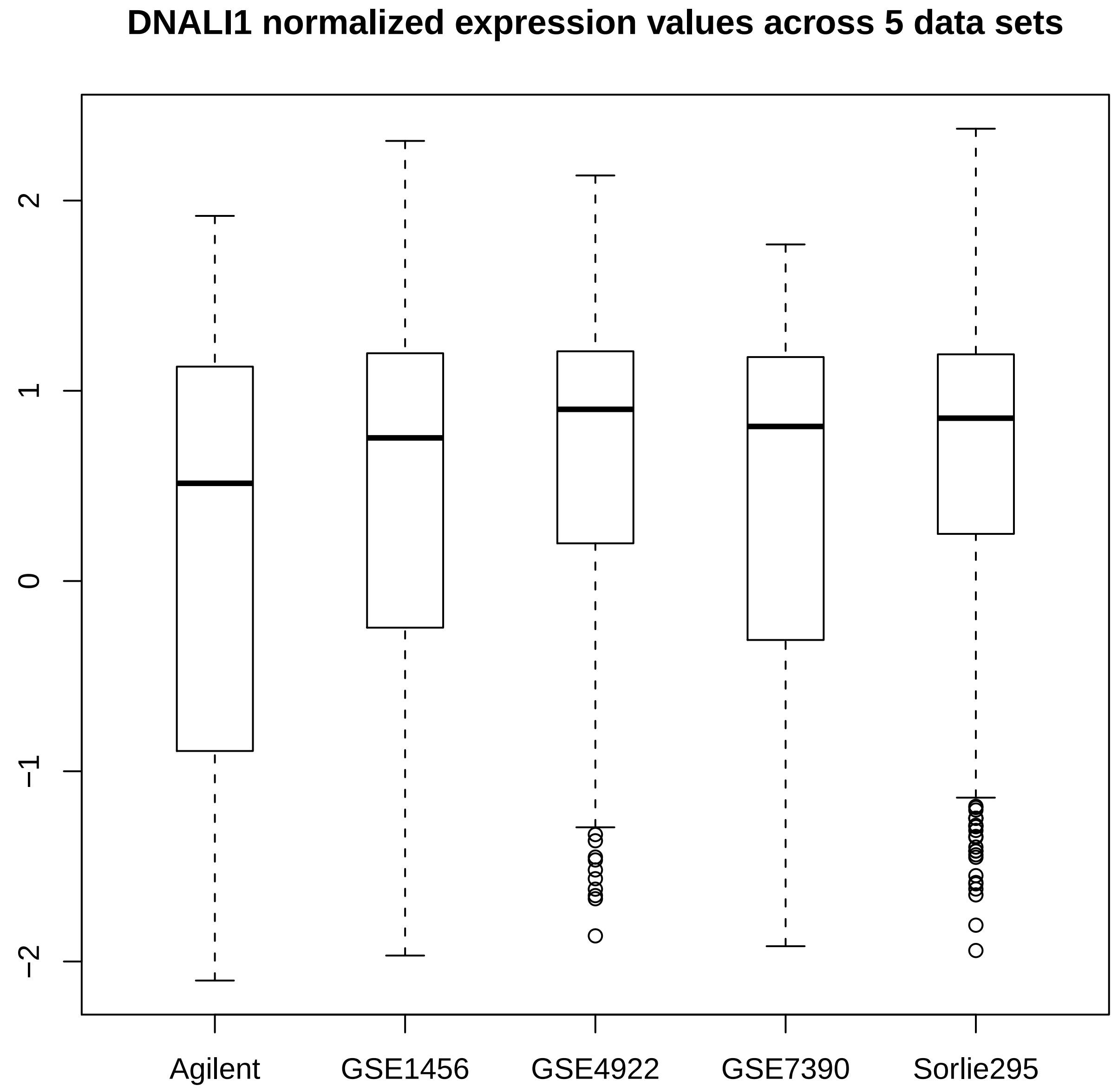

DNALI1 normalized expression values across 5 data sets
2
1
0
−1
−2
Agilent
GSE1456
GSE4922
GSE7390
Sorlie295

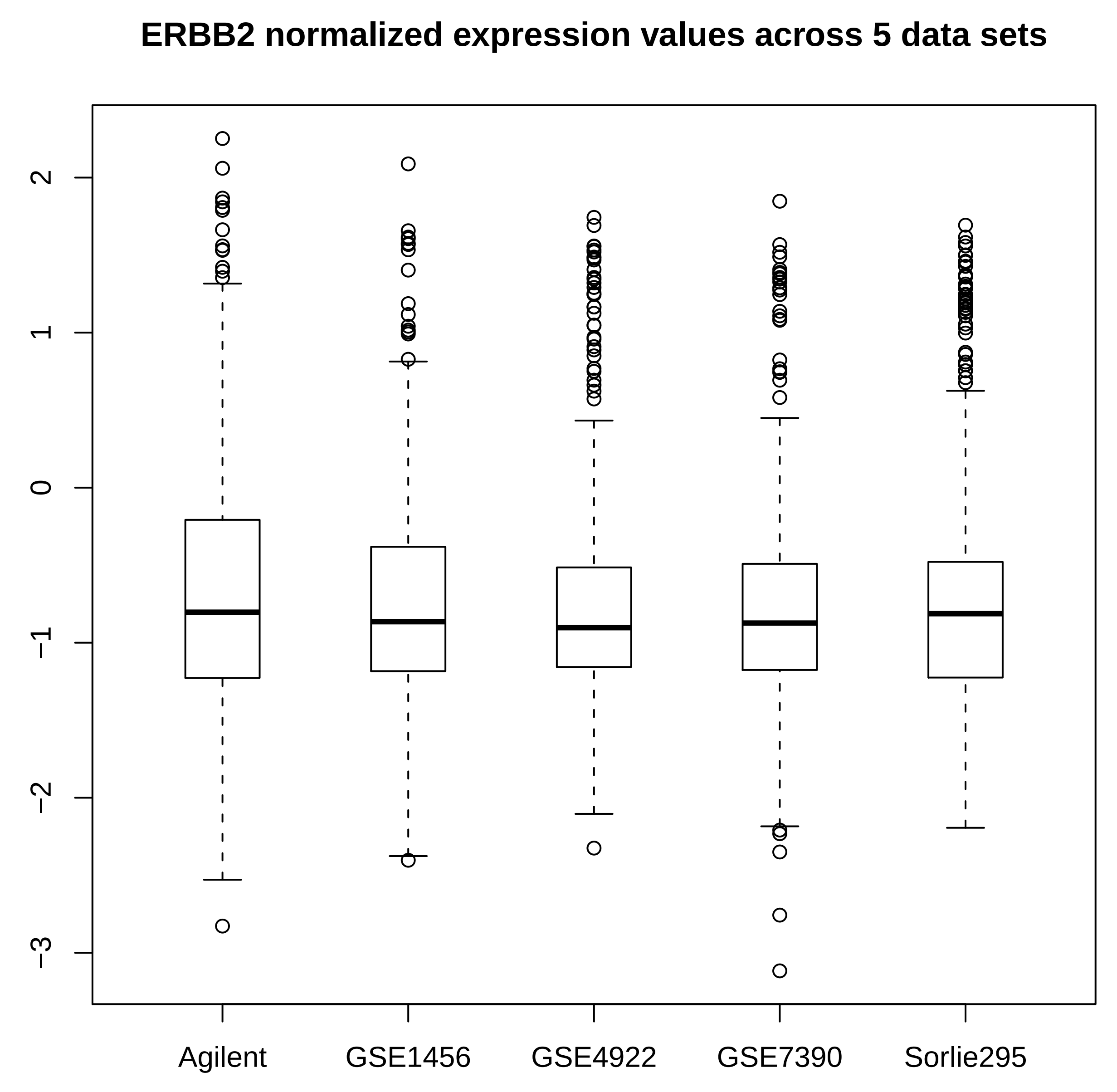
ERBB2 normalized expression values across 5 data sets
2
1
0
−1
−2
−3
Agilent
GSE1456
GSE4922
GSE7390
Sorlie295

ESR1 normalized expression values across 5 data sets

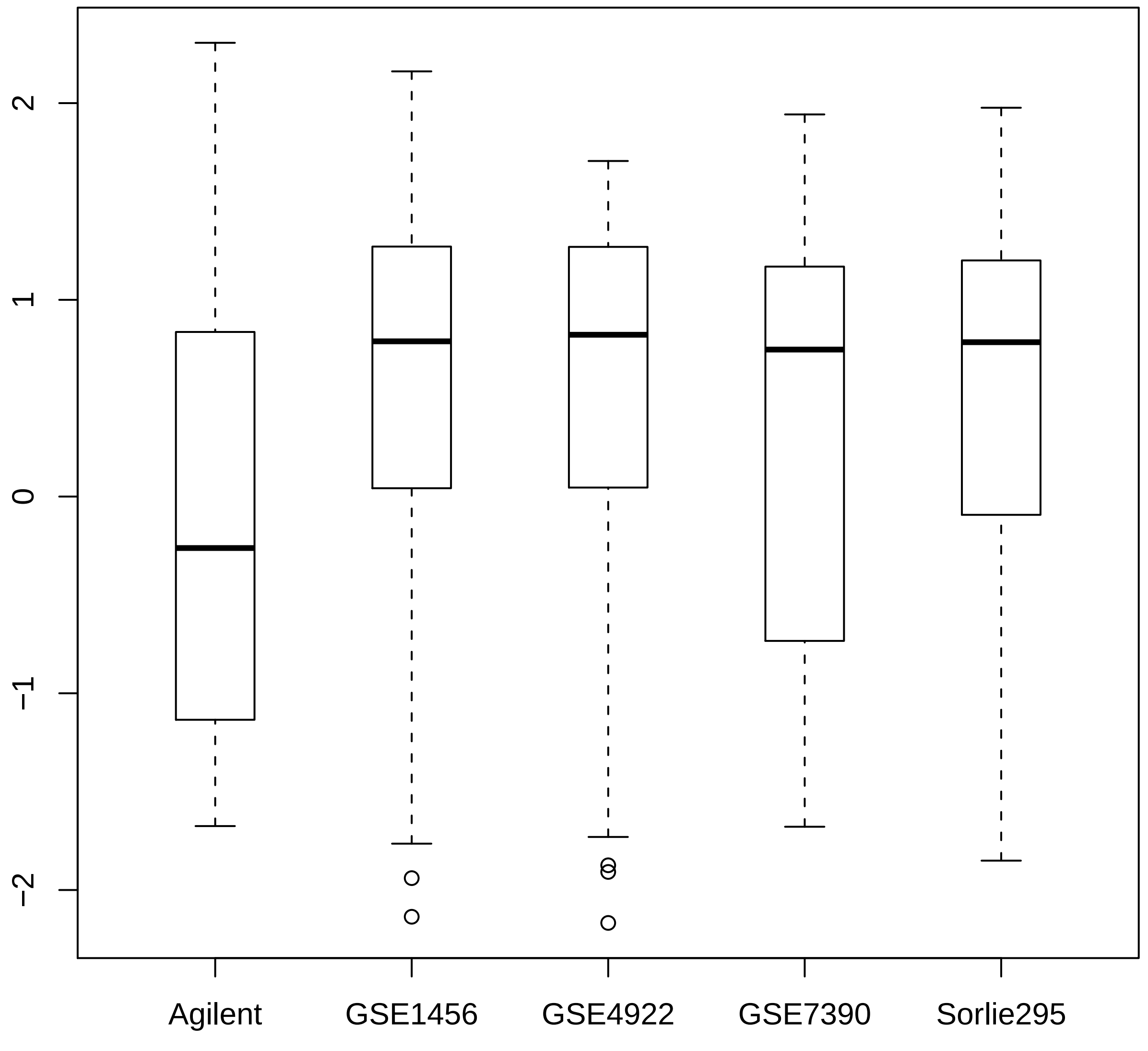

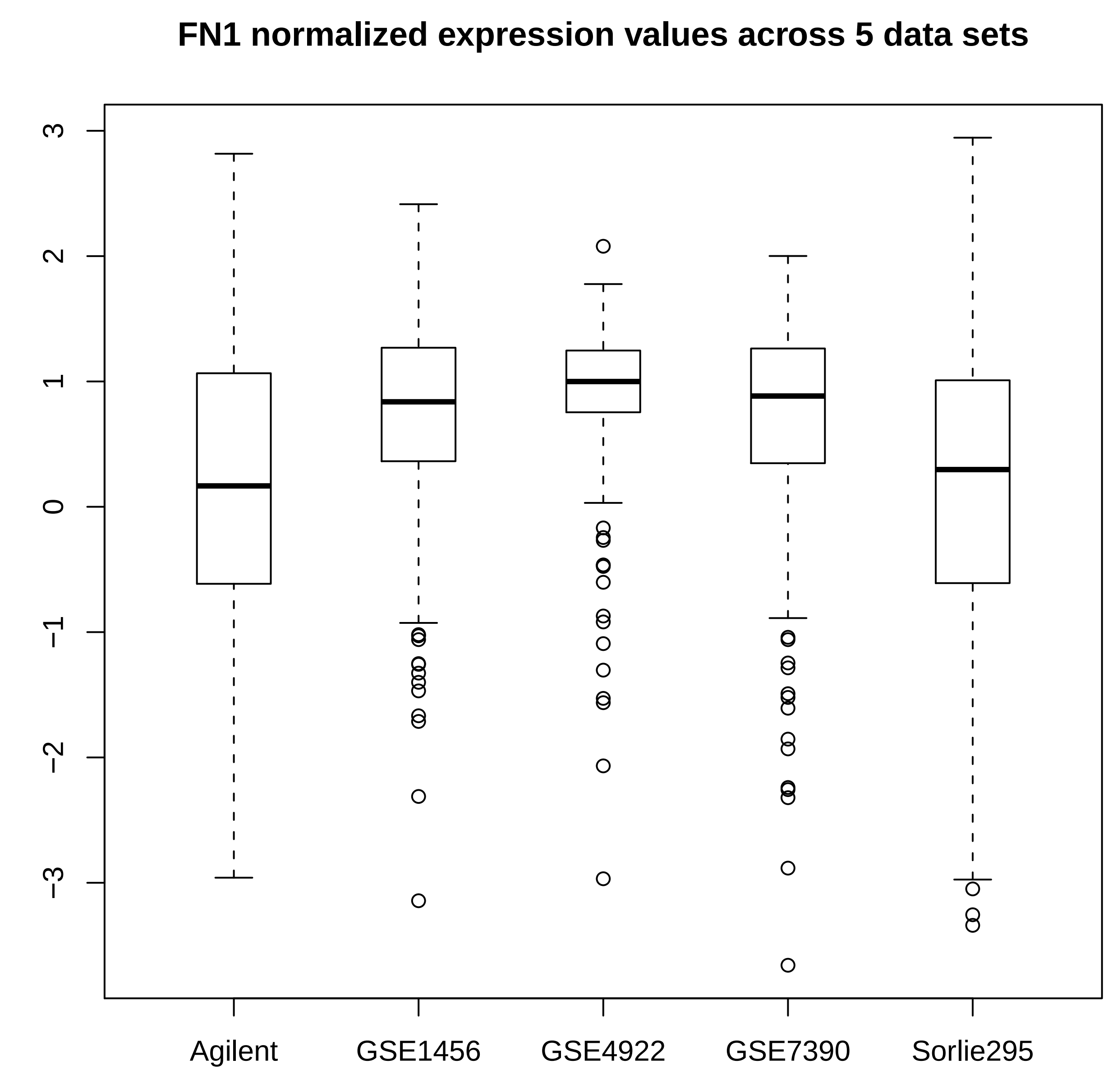
FN1 normalized expression values across 5 data sets
3
2
1
0
−1
−2
−3
Agilent
GSE1456
GSE4922
GSE7390
Sorlie295

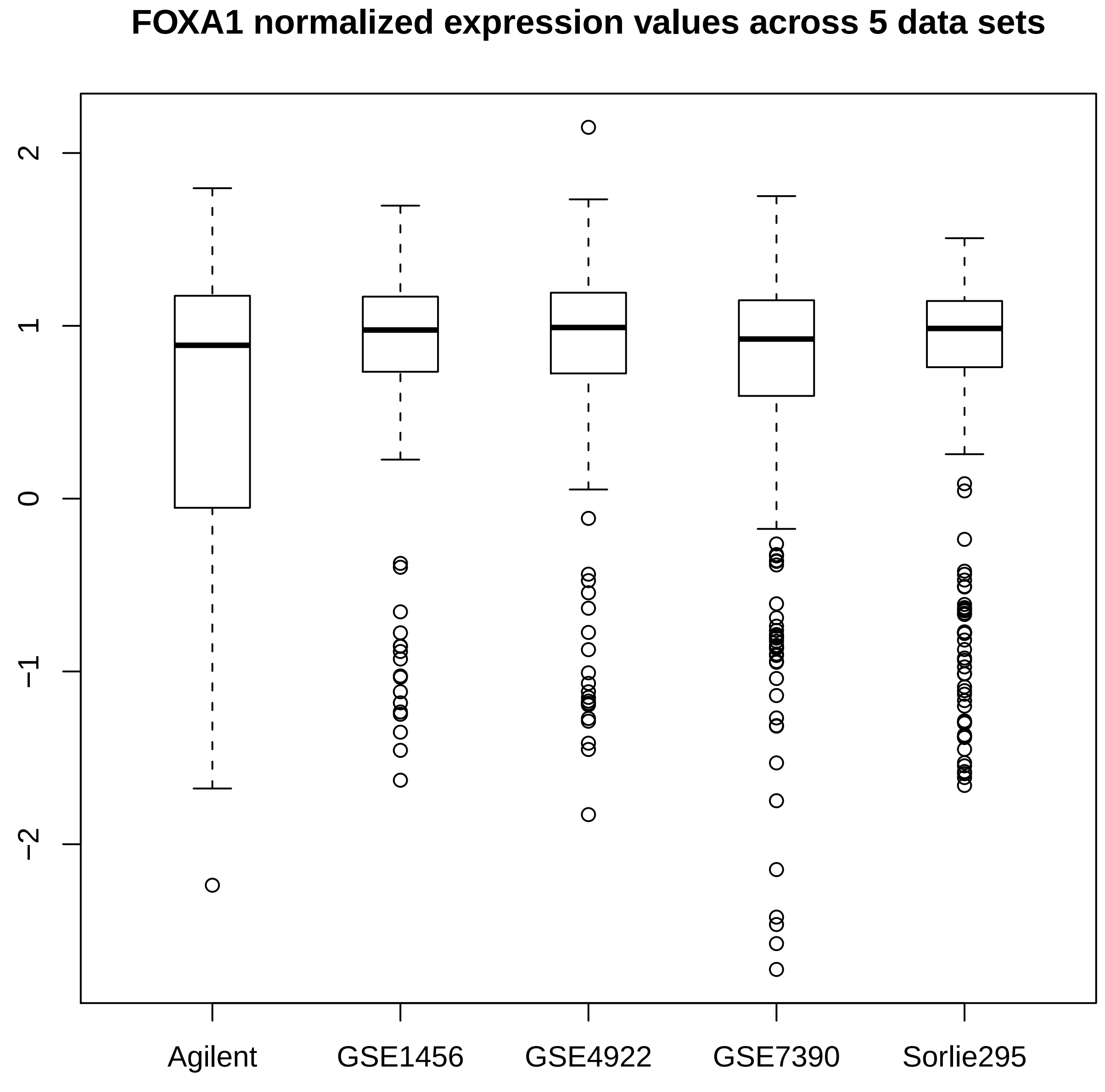

FOXA1 normalized expression values across 5 data sets
2
1
0
−1
−2
Agilent
GSE1456
GSE4922
GSE7390
Sorlie295

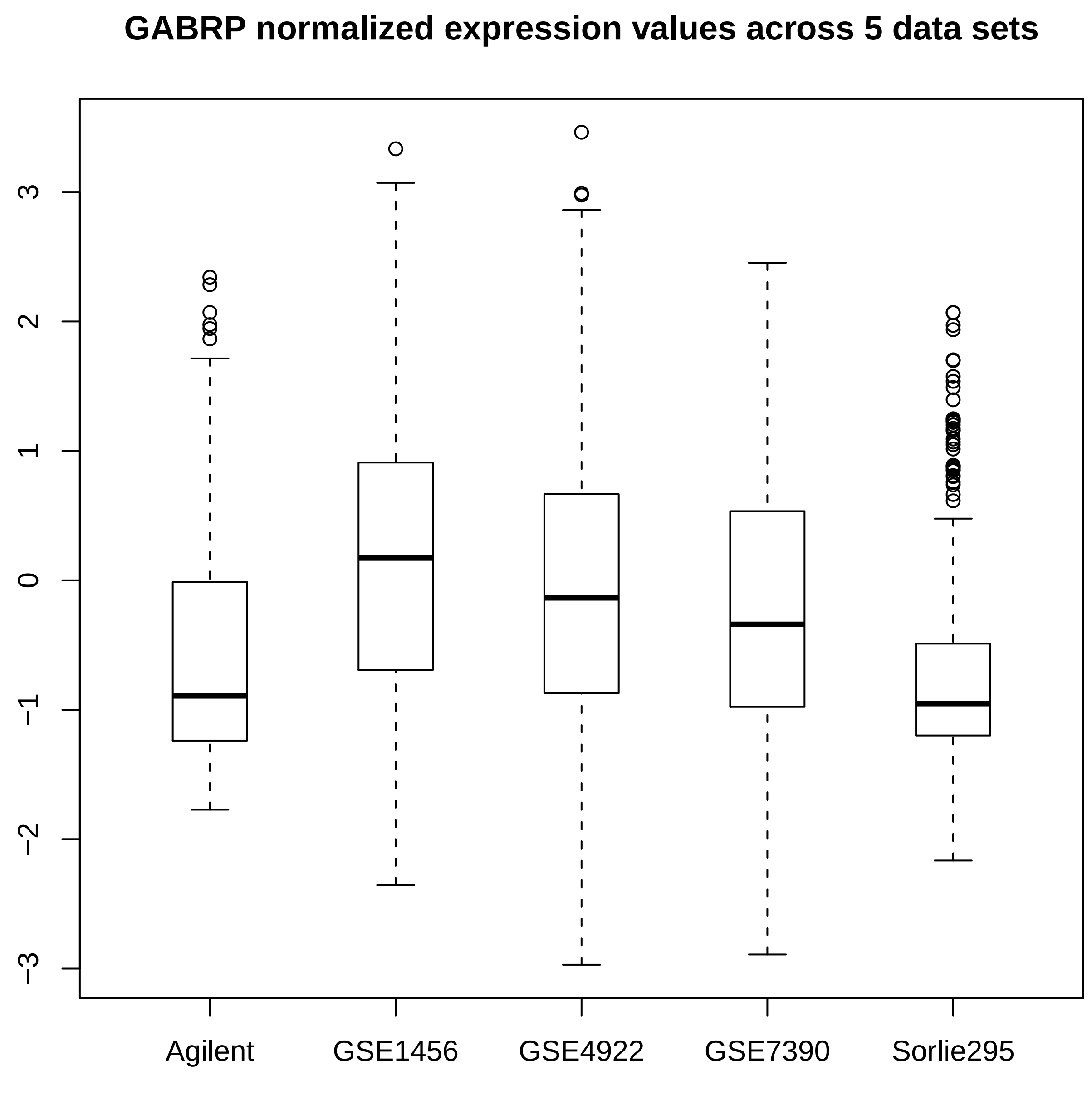
GABRP normalized expression values across 5 data sets
3
2
1
0
−1
−2
−3
Agilent
GSE1456
GSE4922
GSE7390
Sorlie295

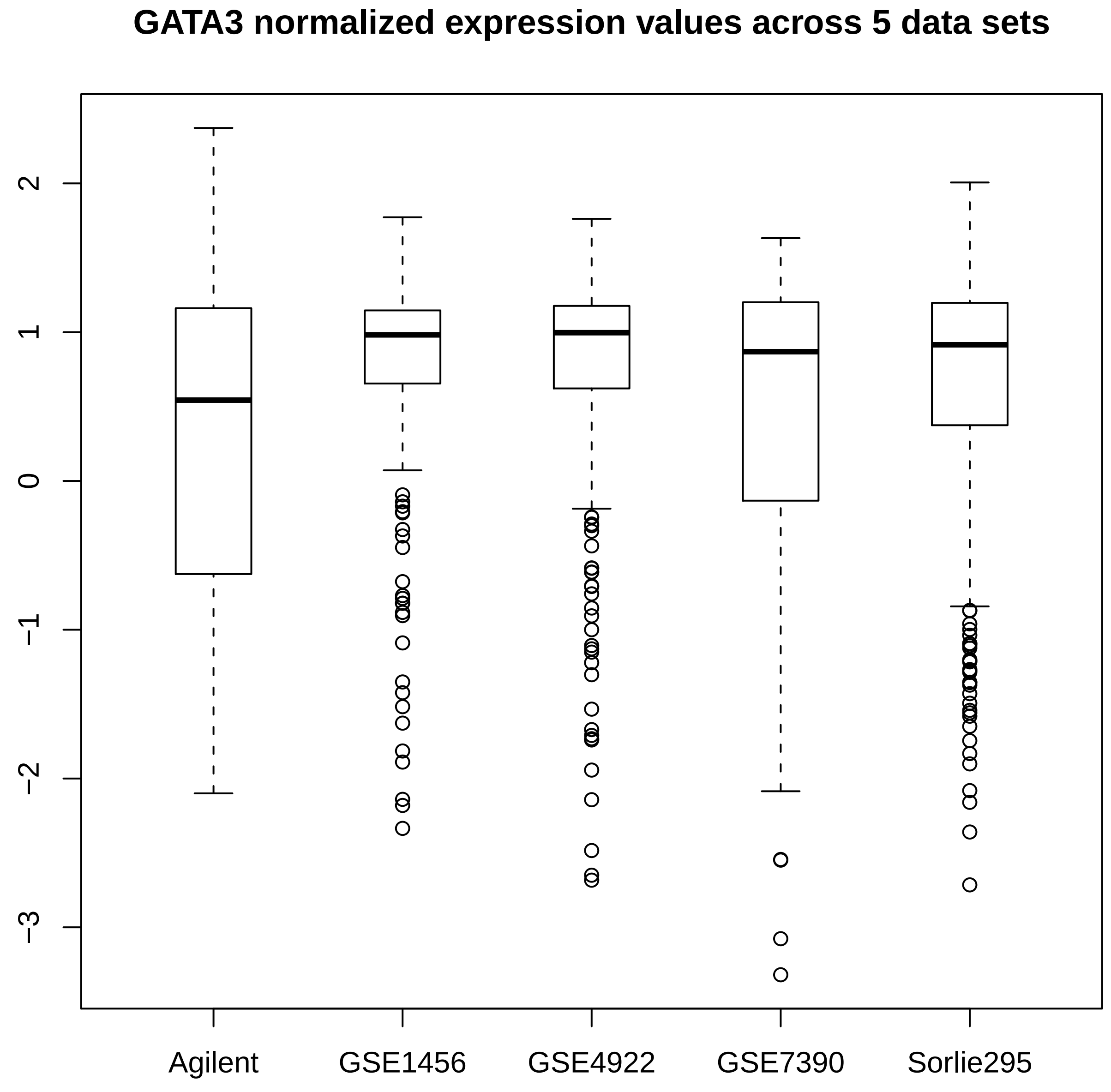
GATA3 normalized expression values across 5 data sets
2
1
0
−1
−2
−3
Agilent
GSE1456
GSE4922
GSE7390
Sorlie295

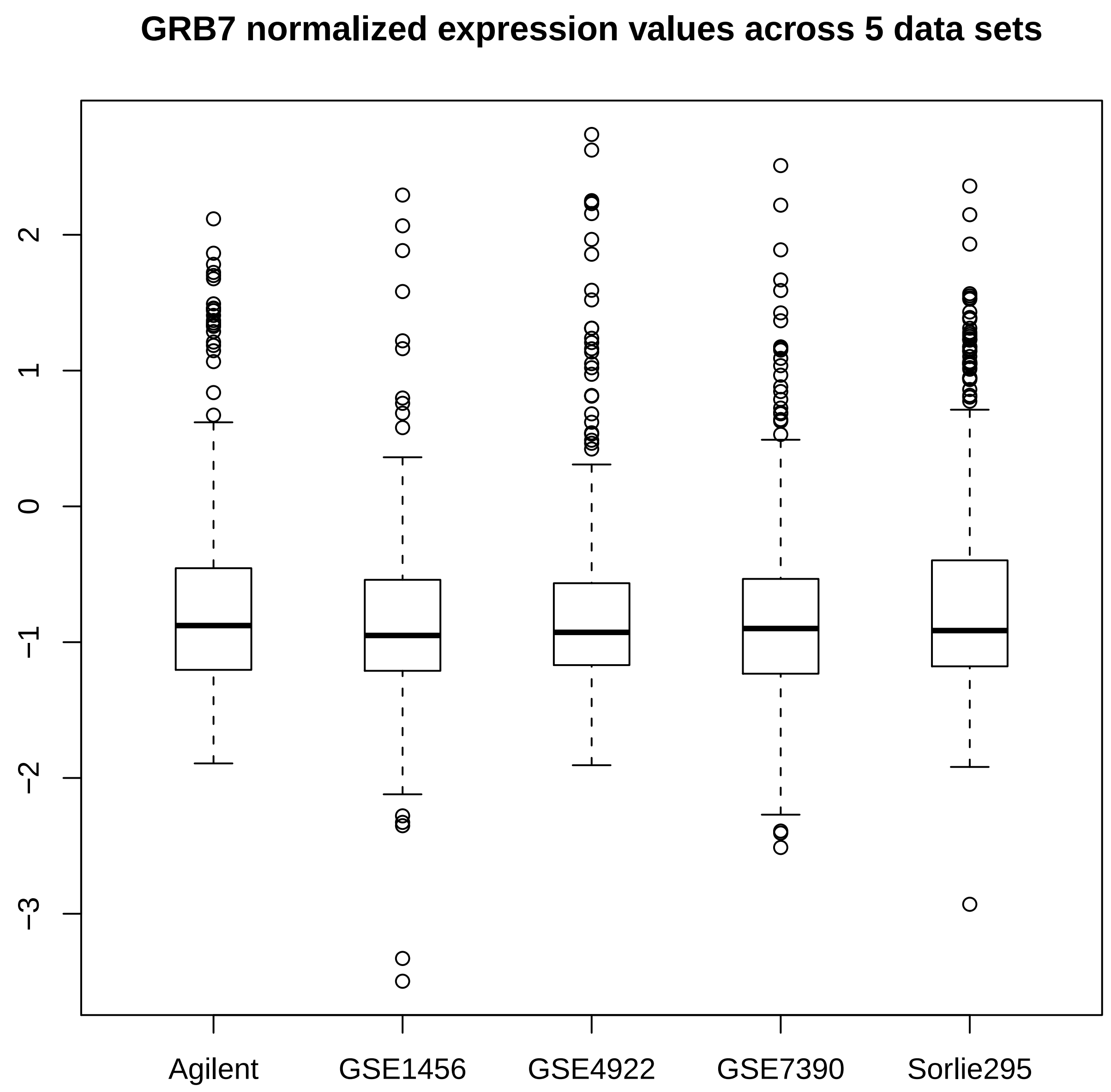

GRB7 normalized expression values across 5 data sets
2
1
0
−1
−2
−3
Agilent
GSE1456
GSE4922
GSE7390
Sorlie295

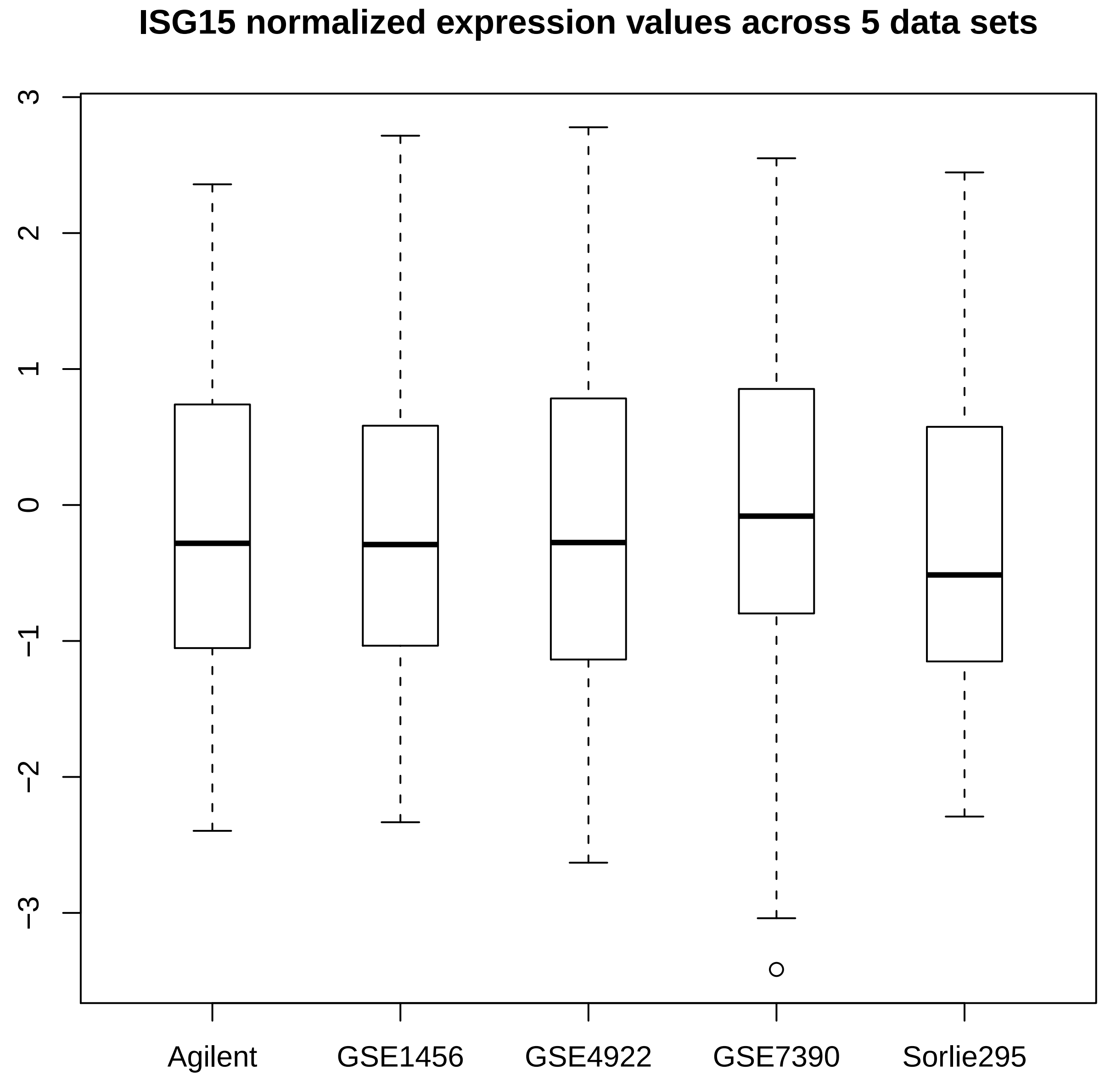
ISG15 normalized expression values across 5 data sets
3
2
1
0
−1
−2
−3
Agilent
GSE1456
GSE4922
GSE7390
Sorlie295

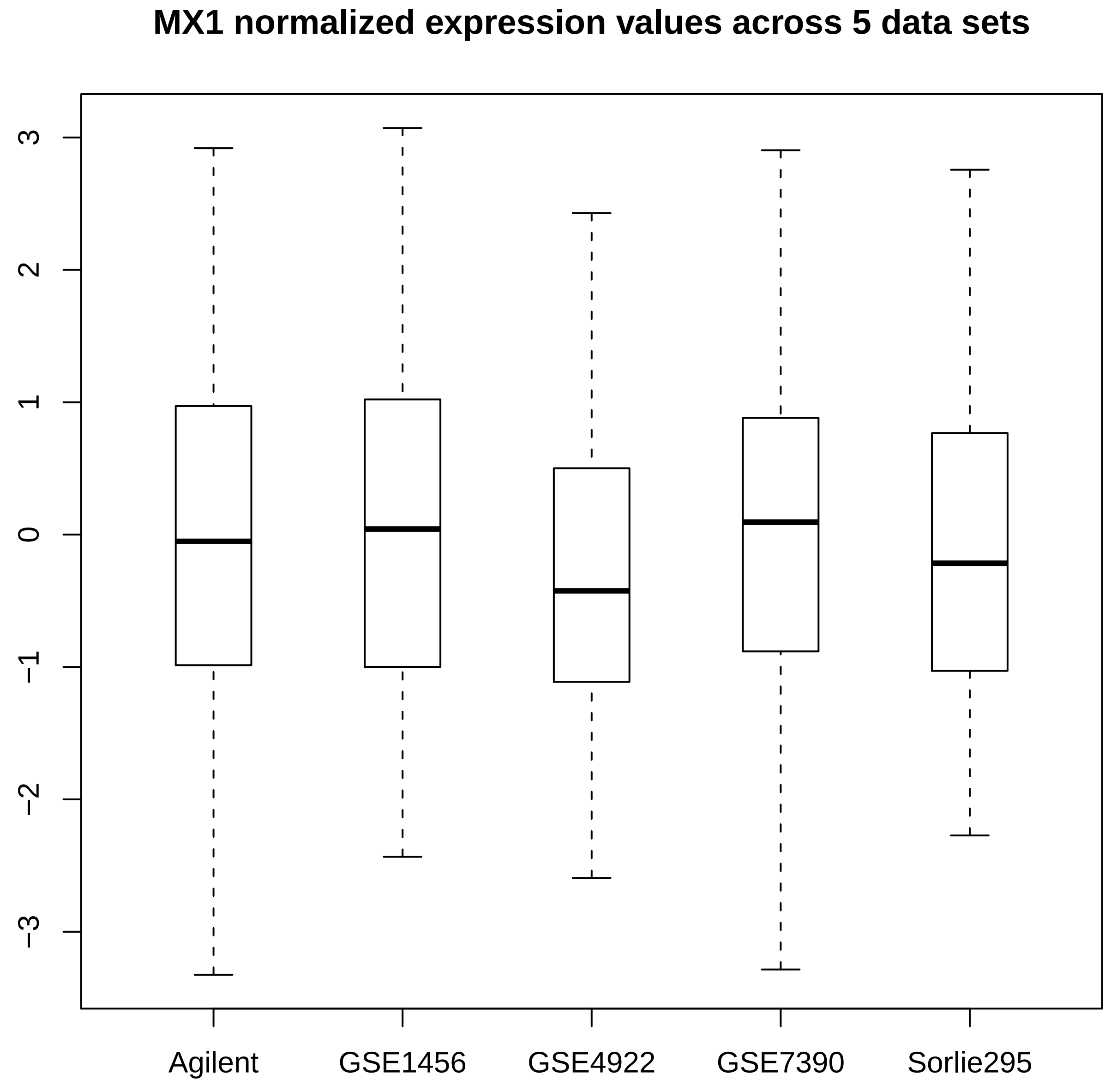
MX1 normalized expression values across 5 data sets
3
2
1
0
−1
−2
−3
Agilent
GSE1456
GSE4922
GSE7390
Sorlie295

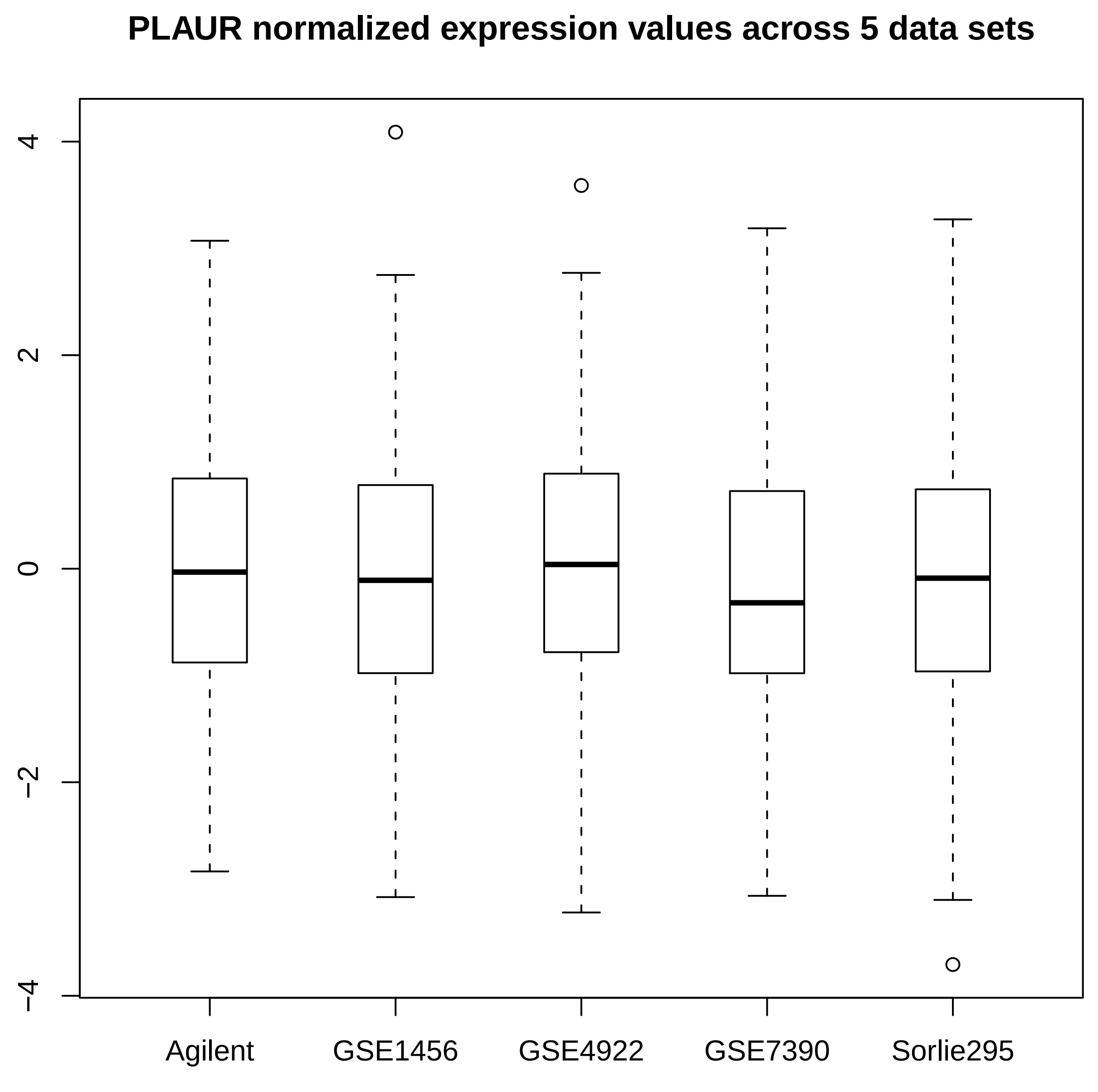
PLAUR normalized expression values across 5 data sets
4
2
0
−2
−4
Agilent
GSE1456
GSE4922
GSE7390
Sorlie295

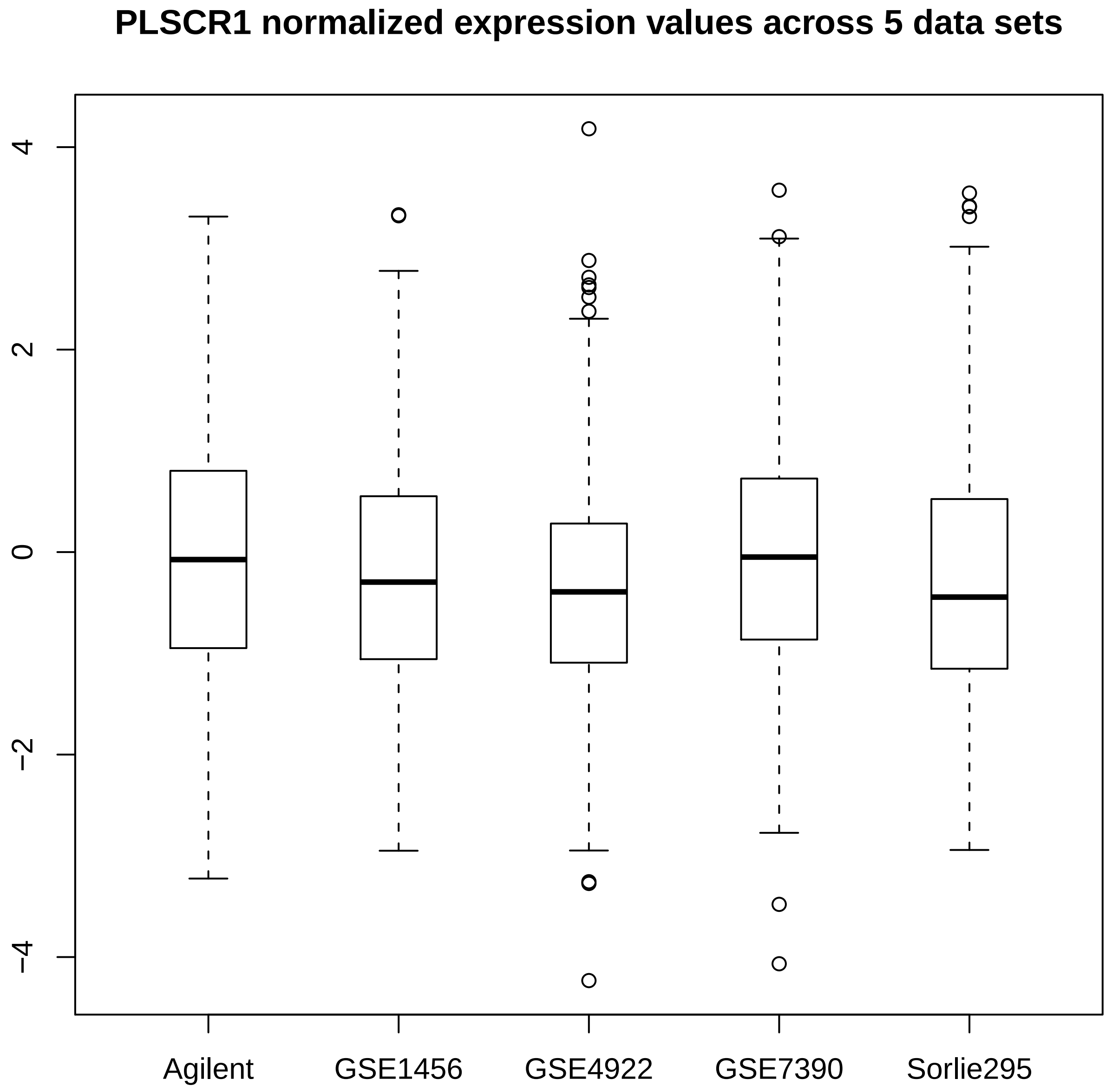

PLSCR1 normalized expression values across 5 data sets
4
2
0
−2
−4
Agilent
GSE1456
GSE4922
GSE7390
Sorlie295

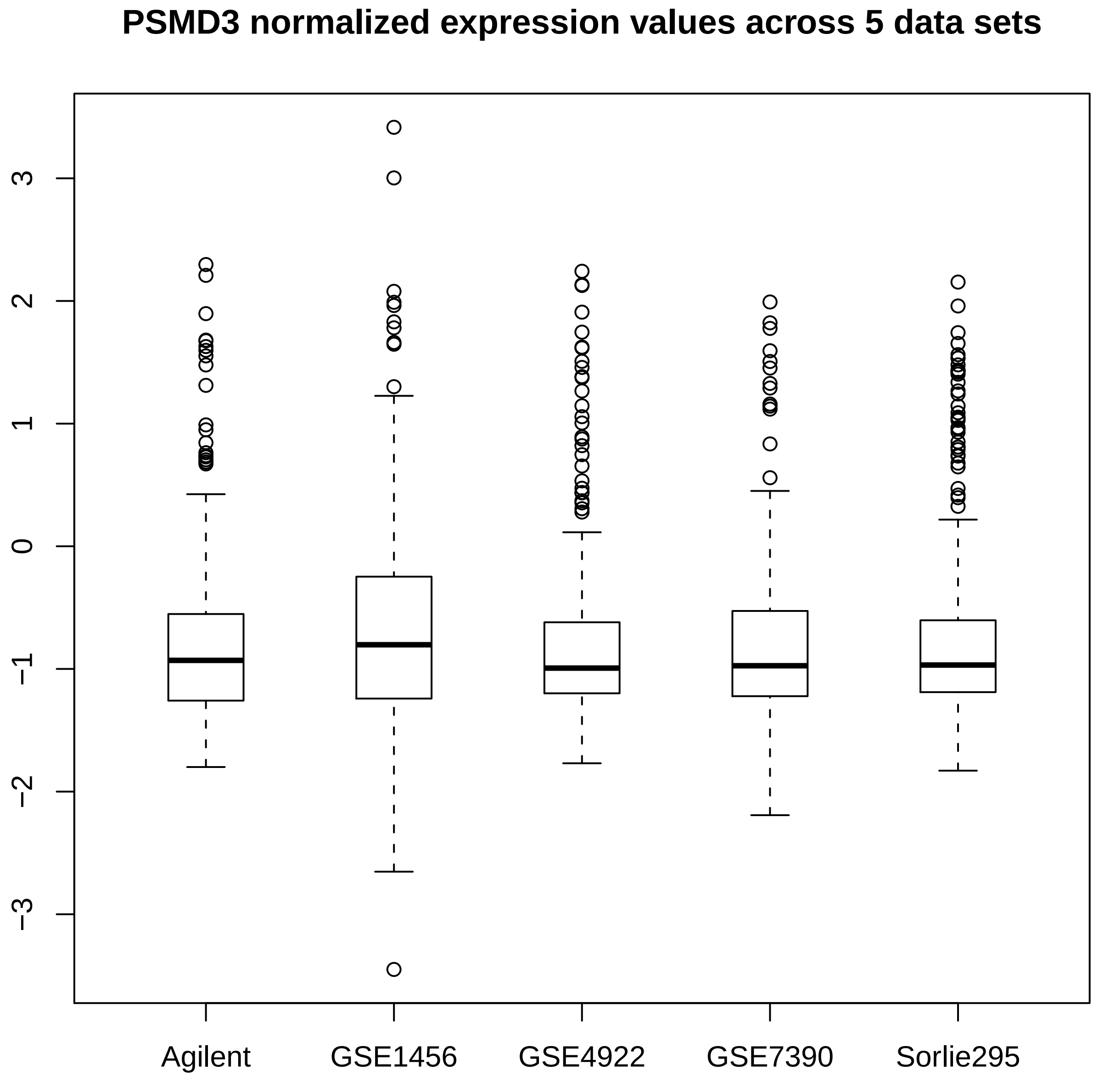
PSMD3 normalized expression values across 5 data sets
3
2
1
0
−1
−2
−3
Agilent
GSE1456
GSE4922
GSE7390
Sorlie295

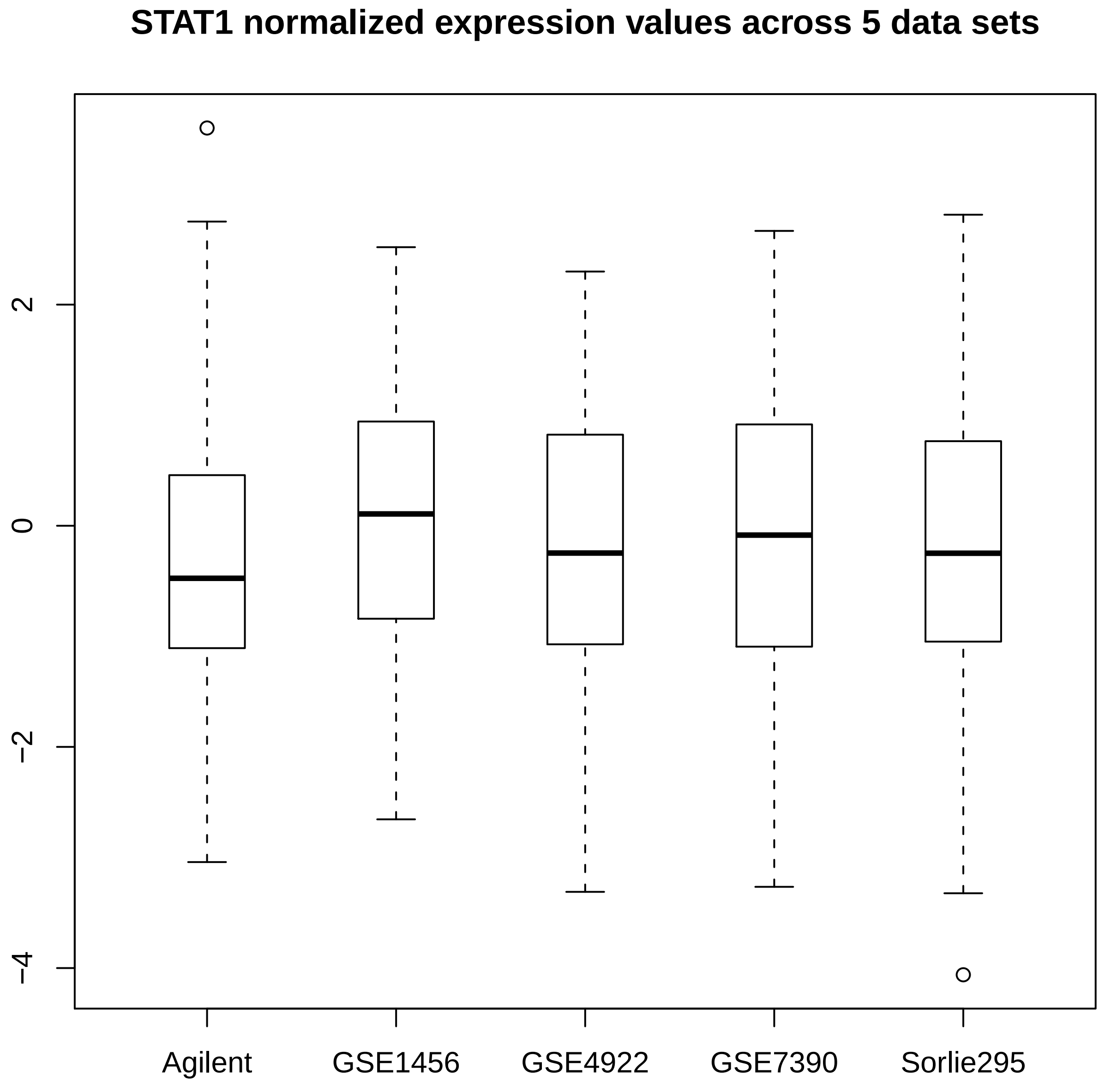
STAT1 normalized expression values across 5 data sets
2
0
−2
−4
Agilent
GSE1456
GSE4922
GSE7390
Sorlie295

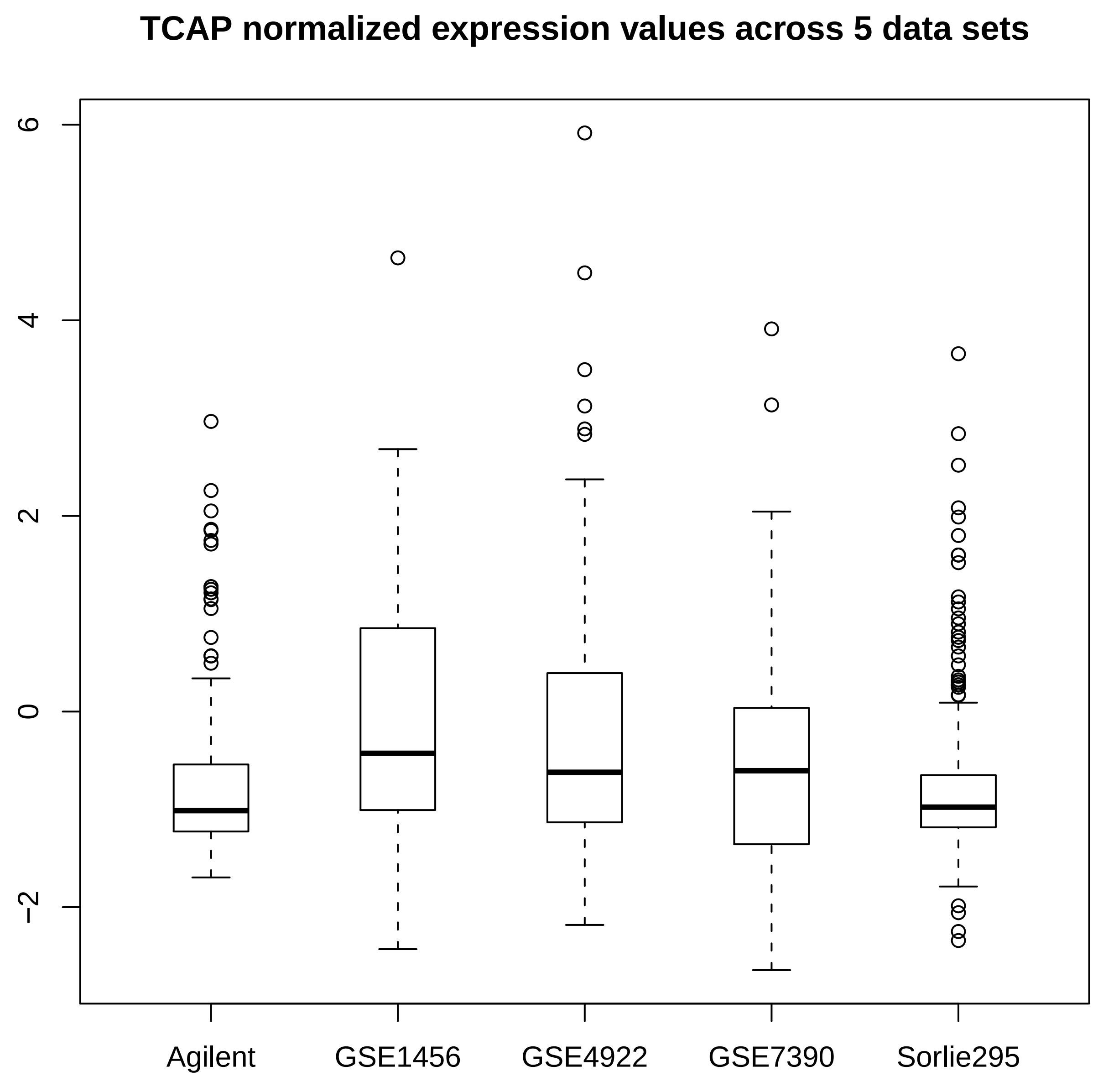
TCAP normalized expression values across 5 data sets
6
4
2
0
−2
Agilent
GSE1456
GSE4922
GSE7390
Sorlie295
